# Mental health service coverage and gaps among adults in Europe: a systematic review

**DOI:** 10.1016/j.lanepe.2025.101458

**Published:** 2025-10-06

**Authors:** Corrado Barbui, Jordi Alonso, Dan Chisholm, Sara Evans-Lacko, Roxanne C. Keynejad, Ledia Lazeri, Numan Miah, Zivile Valuckiene, Chiara Gastaldon

**Affiliations:** a WHO Collaborating Centre for Research and Training in Mental Health and Service Evaluation, Department of Neurosciences, Biomedicine and Movement Sciences, Section of Psychiatry, University of Verona, Verona, Italy; b Health Services Research Group, Hospital del Mar Research Institute, Barcelona, Spain; c Centro de Investigación Biomédica en Red de Epidemiología y Salud Pública, Instituto de Salud Carlos III (CIBERESP, ISCIII), Madrid, Spain; d Department of Medical and Life Sciences, Pompeu Fabra University, Barcelona, Spain; e Department of Mental Health, Brain Health and Substance Use, World Health Organization (WHO), Geneva, Switzerland; f Care Policy and Evaluation Centre, London School of Economics and Political Science, London, UK; g Health Service and Population Research Department, Institute of Psychiatry, Psychology & Neuroscience, King's College London, London, UK; h World Health Organization (WHO) Regional Office for Europe, Copenhagen, Denmark; i Global Mental Health Peer Network, Newcastle, UK; j Global Mental Health Peer Network (Kaunas), Institute of Hygiene (Vilnius), Lithuania

**Keywords:** Treatment gap, Coverage gap, Europe, Mental health

## Abstract

Ensuring the right to the highest attainable standard of mental healthcare requires a clear understanding of the current state of service coverage and gaps across Europe. Given the wide heterogeneity of health systems and resources, systematically assessing these gaps is crucial in order to identify inequities, inform policy and guide efforts to strengthen care at regional and national levels. In this Series paper, we systematically reviewed 45 studies reporting 198 national or sub-national estimates of adult mental health service coverage and treatment gaps in the World Health Organization (WHO) European Region. Data were scarce for many countries and conditions, heterogeneous in definitions, and rarely longitudinal, limiting comparability and trend analysis. Coverage for psychotic disorders was generally higher, often exceeding 90% in some countries but varied widely. For major depressive disorder, minimally adequate treatment ranged from below 10% in Bulgaria, Tajikistan and Turkmenistan to over 35% in Germany and Czechia. Anxiety disorder coverage ranged from 7% in Bulgaria to 47% in Sweden; most substance use disorder estimates were under 15%, and adult ADHD coverage was typically below 10%, based on outdated data. Trend analyses indicated minimal increases in depression coverage over two decades and mixed patterns for psychosis. Marginalised groups, including refugees, homeless populations and sexual minorities, faced the largest gaps, sometimes exceeding 80%. The lack of standardised, repeated measures hampers tracking of progress toward WHO's 2030 goal of a 50% increase in coverage. We advocate that harmonised monitoring systems, with attention to treatment adequacy and equity, are urgently needed to close persistent mental health care gaps across Europe.

## Introduction

Mental health is more than the absence of mental health illness; it is essential for a fulfilling life, enabling individuals to contribute to society, and manage daily challenges.^1^ Growing awareness of its importance has led to calls for comprehensive care, including early identification, treatment, and rehabilitation.^2^

**Key messages**

• There are large gaps in mental health care across Europe, with coverage for psychotic disorders often greater than for common conditions such as depression and anxiety disorders.
• Data on mental health service coverage in Europe are scarce and heterogeneous, limiting the scope for cross-country comparisons and trend analyses.
• The absence of longitudinal data impedes tracking of changes over time, compromising effective monitoring of progress in mental health service provision and the impact of policies.
• To address these gaps, European countries need consistent and harmonised data monitoring systems to track coverage, treatment adequacy, and progress towards WHO's 2030 mental health coverage targets.

**Search strategy and selection criteria**

References for this review were identified by one reviewer (CG) through searches of Medline and PsychINFO with the search terms (Mental Health” [Mesh] OR “Mental Disorders” [Mesh] OR “mental health” OR “mental disorder∗” OR “mental illness∗” OR “psychiatric disorder∗” OR (“psychotic” OR “mood” OR “affective” OR “obsessive-compulsive” OR “panic” OR “depression” OR “anxiety” OR “schizophrenia” OR “somatoform” OR “substance use” OR “suicidal”)) AND (“treatment gap” OR “coverage” OR “mental health service∗” OR “mental health care” OR “access to care” OR “healthcare access” OR “unmet need∗” OR “mental health program∗” OR “health care utilization”) AND “Albania” OR “Andorra” OR “Armenia” OR “Austria” OR “Azerbaijan” OR “Belarus” OR “Belgium” OR “Bosnia and Herzegovina” OR “Bulgaria” OR “Croatia” OR “Cyprus” OR “Czech Republic” OR “Denmark” OR “Estonia” OR “Finland” OR “France” OR “Georgia” OR “Germany” OR “Greece” OR “Hungary” OR “Iceland” OR “Ireland” OR “Israel” OR “Italy” OR “Kazakhstan” OR “Kyrgyzstan” OR “Latvia” OR “Lithuania” OR “Luxembourg” OR “Malta” OR “Moldova” OR “Monaco” OR “Montenegro” OR “Netherlands” OR “North Macedonia” OR “Norway” OR “Poland” OR “Portugal” OR “Romania” OR “Russia” OR “San Marino” OR “Serbia” OR “Slovakia” OR “Slovenia” OR “Spain” OR “Sweden” OR “Switzerland” OR “Tajikistan” OR “Turkey” OR “Turkmenistan” OR “Ukraine” OR “United Kingdom” OR “Uzbekistan”, from January 2000 until November 2024. Articles were also identified through searches of the authors’ own files. We also searched the World Mental Health Survey website for relevant publications (https://www.hcp.med.harvard.edu/wmh/publications.php).

The search strategy was generated by two reviewers (CG and CB). The search strategy was designed to identify studies reporting treatment coverage or treatment gaps among individuals with mental disorders, regardless of population subgroup. We did not apply filters or predefined criteria for specific subgroups, but we included studies focusing on any type of subpopulation if they met our inclusion criteria. The search strategy included both broad and specific terms to capture a wide range of mental health conditions. Inclusion and exclusion criteria are described in the method section. Duplicate records were identified and removed electronically using Mendeley before screening. One reviewer (CG) screened all titles and abstracts for eligible studies and assessed the full texts of these studies. A second reviewer (CB) independently screened 20% of the abstract and titles and 20% of the full texts to assess inter-rater agreement and verify consistency. Each disagreement was solved through discussion between the two authors (CG and CB). The final reference list was generated on the basis of originality and relevance to the scope of this Review.

A rights-based approach to mental health emphasises that everyone is entitled to the highest standard of mental health care.^3^ requiring equitable access for all, including marginalized and underserved populations who often face significant barriers.^4^^,^^5^ This perspective emphasizes holistic, person-centred care that integrates biological, psychological, social, and cultural factors,^3^ and values the active participation of individuals in their care.^3^^,^^4^

Despite widespread acceptance of these principles, health systems frequently struggle to deliver accessible and effective services, especially when demand outpaces capacity.^6^^,^^7^ The COVID-19 pandemic highlighted these challenges, with a global surge in mental health needs—driven by factors like bereavement, isolation, and economic uncertainty—overwhelming even well-resourced systems.^6^^,^^8^ Notably, the WHO reported a 25% global increase in anxiety and depression during the pandemic's first year.^9^ Such increase may reflect both a real increase in incidence and severity of conditions and, to a lesser extent, changes in disclosure and help-seeking behaviors.^10^

**Fig. 1:**
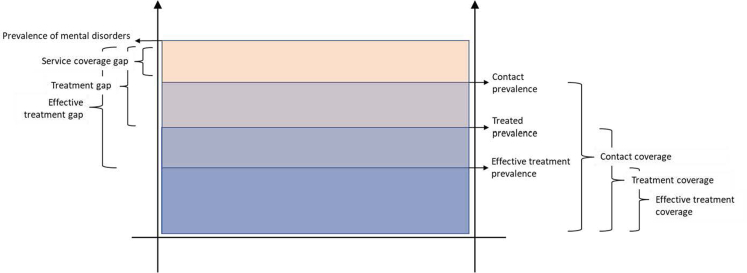
**Graphic representation of definitions of prevalence of mental disorders, coverage, effective coverage and treatment gap**.

Monitoring the balance between mental health service coverage and the prevalence of mental health conditions is a growing priority.^11^ In this context, contact coverage is defined as the proportion of persons with a diagnosable mental health condition in contact with a mental health service.^12^^,^^13^ As not all persons in contact with a mental health service need or receive treatment, “treatment coverage” is the proportion of people with a diagnosis who receive treatment. “Effective treatment coverage” is the proportion of people with a diagnosis who gain the intended health benefit from that treatment (Fig. 1). Some studies have also assessed the coverage of specific treatments or programmes.^14^ Any discrepancy between the prevalence of mental health conditions and their coverage by mental health services demonstrates a gap (Fig. 1).^13^ The “service coverage gap” is a measure of disparity between the number of people estimated to have a diagnosable mental condition and the proportion in contact with mental health services. Similarly, the “treatment gap” is a measure of the disparity between the number of people estimated to have a mental health condition and the proportion of those who receive treatment.^13^ Finally, the “effective treatment gap measures” the disparity between the number of people estimated to suffer from a mental health condition and the proportion who receive treatment resulting in health benefits (Fig. 1).^13^

Twenty years ago, a WHO report analysing service use data from over 30 studies found that the treatment gap for mental disorders was large, but varied by region.^15^ In European countries, the average treatment gap was 45% for major depression, 40% for bipolar disorder, and 18% for schizophrenia. Similar findings were obtained by WHO Mental Health Surveys, which addressed the treatment gap in relation to different levels of severity.^16^ Although severity of the disorder was associated with the greater likelihood of treatment in almost all countries, 35.5%–50.3% of severe cases in high-income countries and 76.3%–85.4% in low and middle-income countries received no treatment in the preceding 12 months. Across European countries, these estimates ranged from 35.5% of severe cases in Spain not receiving treatment in the preceding 12 months to 80.3% in Ukraine.^16^

To monitor and evaluate efforts to expand mental health services within and across countries, it is necessary to measure mental health service coverage and whether service, treatment and effective treatment gaps have reduced over time.^6^ Service coverage is a key target of the WHO Comprehensive Mental Health Action Plan 2013–2030, with a goal of increasing coverage by 50%. These data are therefore essential for tracking progress toward this target.^17^ Against this background, we aimed to systematically review and narratively synthesize quantitative studies on mental health service coverage and treatment gaps among adults with any mental health conditions across countries in the WHO-defined European Region. Secondary aims were to describe and compare coverage or gap estimates for different mental health conditions across European countries, to highlight gaps in the available evidence and evaluate trends in coverage and gap estimates over time if available. We did not restrict the review to a predefined list of diagnoses, in order to comprehensively map the available estimates across countries and conditions.

## Methods

We followed Preferred Reporting Items for Systematic Reviews and Meta-Analyses (PRISMA) extension for Synthesis WIthout Meta-analysis (SWIM).^18^ The SWIM checklist is reported in the supplement. The protocol was prospectively registered and is available via Open Science Framework (https://osf.io/usvb6/?view_only=a7cd852f7142448098fe880b6837db0b).

We conducted a systematic literature search to identify studies examining the treatment gap and coverage of adult mental health care in Europe (Search strategy and selection criteria). We restricted our search to studies reporting data from European countries, as defined by the WHO European Region. Only studies that quantitatively estimated the mental health treatment gap or coverage among adults with any mental health condition at the national or sub-national level (i.e., specific regions within countries) were included. Studies focused on children and adolescents were excluded. Only studies published in the last 25 years (1999–2024) were included, to capture recent developments. Any study design reporting quantitative outcomes was eligible for inclusion, provided that it reported empirical evidence. We excluded systematic reviews and meta-analyses as we sought primary data rather than aggregated findings. We searched for the lists of included studies in otherwise eligible systematic reviews for potentially eligible studies.

For each included study one review author (CG) extracted the following data: study ID, type of study, time period covered by the study, country or region, population's characteristics (mental disorders, mean age, inclusion criteria, if applicable type of subgroup), type of measure (treatment coverage versus gap), estimates of treatment gap and/or coverage. A second reviewer (CB) checked the correctness of data extraction.

Coverage was defined as the proportion of individuals with a mental health condition who received any form of mental health treatment, based on service utilisation data matched to population prevalence estimates (e.g., Global Burden of Disease data). The treatment gap was defined as the proportion of individuals with a mental disorder who were neither diagnosed nor treated. We included studies conducted at any level of care (e.g., primary care, secondary care).

We considered studies reporting coverage or treatment gaps in mental health care using any measure (e.g., crude numbers, ratios, percentages) at the national or sub-national level. Only coverage data were considered when the same study's coverage and gap estimates were available. If only gap estimates were available, the corresponding coverage was calculated as reported in the Supplementary Methods.^14^ The risk of bias was assessed using the tool for studies measuring the prevalence of mental health disorders (RoB-PrevMH), which assesses the representativeness of the sample and responders and risk of bias of outcome measurement.^19^

We presented results as estimated treatment coverage for each country (or region, if available) and each mental health condition in a box plot, a table with estimates and uncertainty intervals when available and a table with characteristics of all included studies. In cases where multiple years of data were available for the same country and diagnosis, the most recent data were prioritised as the primary result. In contrast, earlier data were presented as secondary results.

Considering the expected heterogeneity of study designs, populations, and outcome measures due to the inclusion of several countries over a period of 25 years, we conducted a narrative synthesis of the findings, reporting coverage estimates separately for each country and condition, giving priority to the most recent estimates. Narrative synthesis is an established method for systematically summarizing and explaining evidence from multiple studies when statistical meta-analysis is not feasible or appropriate. Because no quantitative pooling of effect estimates or direct comparison of interventions was performed, we did not apply the GRADE (Grading of Recommendations Assessment, Development and Evaluation) framework, which is primarily intended for rating the certainty of evidence in quantitative syntheses.

## Results

### Study selection

**Fig. 2:**
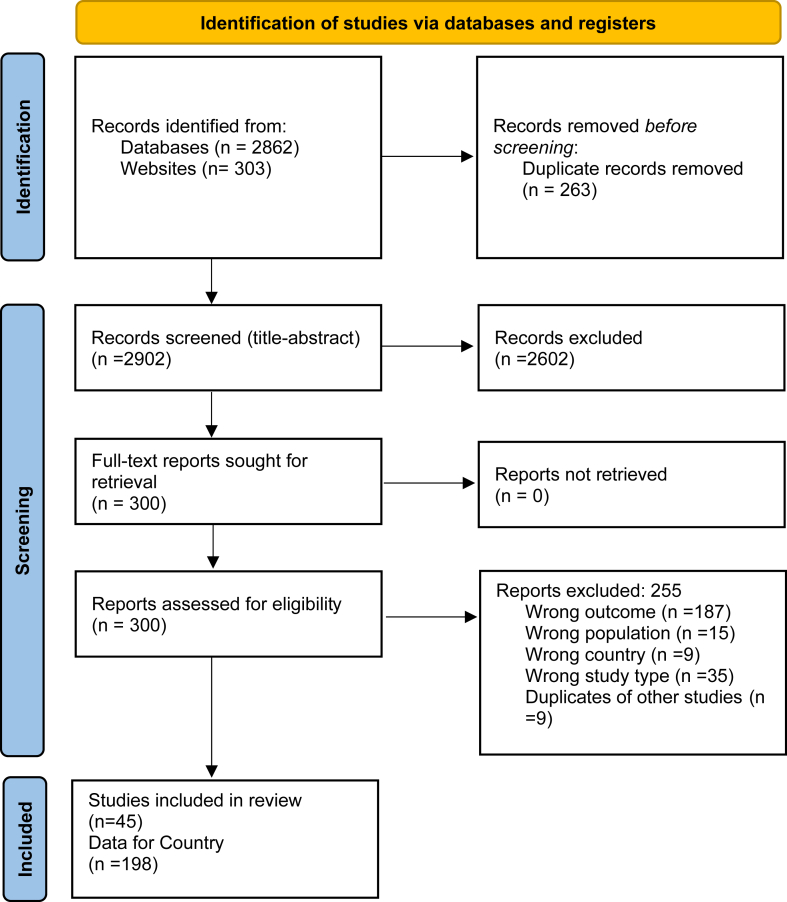
**PRISMA flow-chart of study screening and selection**.

The systematic search identified a total of 3165 records from databases and websites. After removing 263 duplicates, we screened 2902 records based on titles and abstracts. Of these, we excluded 2602 for not meeting the inclusion criteria. We reviewed 300 full-text reports after which 255 studies were excluded (a list of excluded studies with reasons is provided in the supplement). Agreement between the first (CG) and second reviewer (CB) with the first reviewer was high (93.3%), with a Cohen's kappa of approximately 0.89, indicating strong agreement. Discrepancies were resolved through discussion. A total of 45 studies met inclusion criteria, reporting 198 estimates of treatment coverage at country or sub-national level (Fig. 2, Tables S1 and S2).^20^, ^21^, ^22^, ^23^, ^24^, ^25^, ^26^, ^27^, ^28^, ^29^, ^30^, ^31^, ^32^, ^33^, ^34^, ^35^, ^36^, ^37^, ^38^, ^39^, ^40^, ^41^, ^42^, ^43^, ^44^, ^45^,^46^, ^47^, ^48^, ^49^, ^50^, ^51^, ^52^, ^53^, ^54^, ^55^, ^56^, ^57^, ^58^, ^59^, ^60^, ^61^, ^62^, ^63^, ^64^ A detailed table of the number of included studies per country and their corresponding income classification is reported in Table S3.

Table 1 provides an overview of the characteristics of included studies, while Table S1 reports the characteristics of each included study with estimates for each country and diagnosis. Most studies provided nationally representative population-level data on the general population, while 10 focused on specific sub-groups of people with specific vulnerabilities, such as sexual minorities, refugees, internally displaced and homeless people or low-income groups, women with specific vulnerabilities, and occupational and student populations (Table 1). Studies assessed the coverage of major depressive disorder (MDD), anxiety disorders (i.e., generalized anxiety disorder, specific phobia and panic disorder), post-traumatic stress disorder (PTSD), psychotic disorders, substance and alcohol use disorders (SUD and AUD), and attention-deficit/hyperactivity disorder (ADHD). Data sources varied and included national health surveys (e.g., The World Mental Health Survey), administrative records (e.g., WHO Mental Health Atlas Project), and modelling studies.^16^^,^^22^^,^^25^ Most of the data came from World Mental Health Survey reports.^16^ Additional information about the WHO Mental Health Atlas Project and World Mental Health Survey is reported in the Supplement (“Additional information on the main sources”). The majority of included studies focused on treatment coverage (Table 1). On average the risk of bias was low, as shown in Table S4 and Figure S1 of the Supplemental Material.

**Table 1:** Characteristics of included studies.

Category	Count	Percentage
Total number of estimates per country	198	100.0
Type of design	–	–
Survey/cross-sectional	169	85.4
Cohort longitudinal study	3	1.5
Others	26	13.1
Representativeness	–	–
Nationally representative	139	70.2
Regionally representative	6	3.0
Type of population	–	–
General population	161	14.6
Subgroup	37	58.1
Vulnerable groups	–	–
Yes	188	94.94
No	10	5.05
Income	–	–
LMIC	4	2.0
UMIC	21	10.1
HIC	171	86.4
Geographical location	–	–
Western Europe	130	65.7
Central Europe	16	8.1
Eastern Europe	19	9.6
South-Eastern Europe	32	16.2
Estimate type	–	–
Treatment coverage	183	92.4
Treatment gap	15	7.6

Legend: LMIC, Low- and middle-income countries; UMIC, Upper-middle income countries; HIC, High-income countries.

### Treatment coverage by country or sub-country and disorder

**Fig. 3:**
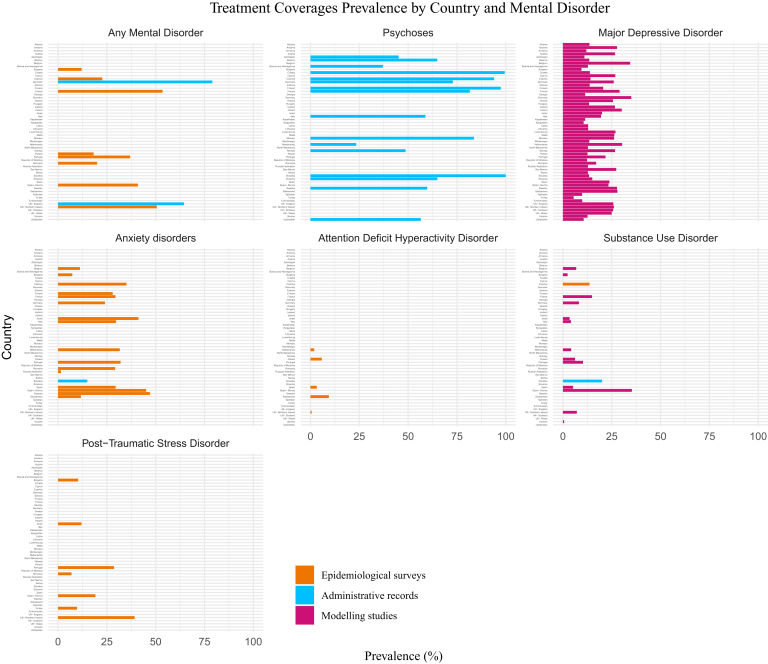
**Treatment coverage prevalence by country and mental disorder**.

Across the European region, there was substantial variation in treatment coverage across both countries and mental health conditions (Fig. 3). Table S2 shows coverage estimates by country (or sub-country/region) and by diagnosis.

For psychotic disorders, all data were estimated through administrative records (as shown in blue in Fig. 3 and Table S2). Most data came from the WHO Mental Health Atlas Project, which assessed service coverage in several European countries in 2017 and 2020. It generally reported high levels of coverage (Fig. 2).^22^ Croatia reported coverage of 99% in 2017, followed by Finland (97%) and Czechia (94%). Conversely, low coverage rates were observed in the Netherlands (23%) and Bosnia and Herzegovina (37%). Another study, which also estimated unmet needs in Slovakia using administrative data from the National Health Information Center, reported very high coverage for psychosis (100%, range 56–100). Of note, coverage estimates were not available for 21 countries.^51^

For MDD, data came from a modelling study based on the data available from the World Mental Health Survey, which primarily estimated minimally adequate treatment (MAT), including either pharmacotherapy (≥1 month of a medication, plus ≥4 visits to any type of medical doctor) or psychotherapy (≥8 visits with any professional including religious or spiritual advisor, social worker or counsellor) (reported in purple in Fig. 3 and Table S2).^25^ As Fig. 3 shows, MAT rates ranged from 9.5% in Bulgaria, 9.8% in Tajikistan and 9.9% in Turkmenistan to 30% in the Netherlands, 34% in Belgium, 35% in Germany, and 36.1% in Czechia (Fig. 2, Tables S1 and S2). Estimates were available for all European countries.^25^

For anxiety disorders, treatment coverage estimates ranged from 7.3% in Bulgaria to 47.1% in Sweden, but there were no published estimates for 40 countries (Fig. 3, Tables S1 and S2). For PTSD, Northern Ireland reported one of the highest coverage estimates at 39.2% (Fig. 3, Tables S1 and S2).

For SUDs, data on coverage were scarce. Of note, 42 European countries lacked a coverage estimate. Those estimates which were available came from a modelling study of the World Mental Health Survey, which estimated MAT in 13 European countries (in purple in Fig. 3),^47^ from an epidemiological survey estimating coverage in Czechia,^29^ and from a study based on administrative records (20% coverage) in Slovakia.^51^ Available MAT estimates were low, ranging from 0.6% in Ukraine, 2.4% in Bulgaria and 3.4% in Israel to 10.3% in Portugal, 14.9% in France, and 35.3% in Spain Murcia (Fig. 3).

Similarly, for adult ADHD, estimates were available for only 10 countries (Fig. 3, Tables S1 and S2). In these countries, treatment coverage was estimated to be 0% in France (2002), Germany (2002), Italy (2002), Portugal (2009) and Belgium (2002), 0.6% in Northern Ireland (2007), 2% in the Netherlands (2003), 3% in Spain (2002), 6% in Poland (2011) and 9.4% in Switzerland (2018). Coverage was estimated only in the early 2000s, and more recent estimates—particularly after ADHD began to be recognized—were not available.

### Adequacy of treatment

Different studies estimated different types of coverage. In particular, Santomauro and colleagues, who provided most estimates for MDD,^25^ measured MAT, whereas the Mental Health Atlas Project, which provided most estimates for psychosis, focused on service coverage. Data on anxiety disorders focused on both any treatment coverage and ‘possibly adequate’ treatment.

Studies that distinguished between any mental health treatment and minimally or possibly adequate treatment showed gaps. For example, for anxiety disorders, 35.7% of people received any treatment in Belgium, but only 11.2% received adequate treatment, 29.4% versus 13.7% in France and 32% versus 11% in Portugal (Table S1).

### Trends over time

For some countries, data were available from several time points, making it possible to assess trends (Supplement S5–S7). For MDD, trends showed small increases in coverage from 2000 to 2021; for psychosis, data on trends were limited and heterogeneous. There were no available data regarding trends in coverage over time for any other diagnoses.

When estimating trends in MAT for MDD from 2001 to 2021 for all European countries, Santomauro et al. found relatively stable coverage (Supplement S7). The most notable increase was observed in Cyprus, where MAT increased by 4.8%. Nine countries (Finland, Malta, Germany, Portugal, Israel, Belgium, UK–Scotland, Russian Federation and Slovenia) experienced more modest increases ranging from 1.8% to 2.8%. The remaining countries had an increase ≤1.7% in MAT for MDD (Supplement S7).

For psychotic disorders, trend data were available from a limited number of studies, with some evidence of a decline in estimated coverage over time. For instance, in Italy, estimated coverage declined from 81% in 2017 to 59% in 2020, in Monaco from 97% to 84%, and in Slovenia from 72% to 65%. It was, however, unclear whether these differences reflected true reductions in coverage or differences in how countries estimated coverage in the two time points. Conversely, in some countries, estimated service coverage for psychosis increased by between 10% (Belarus and Slovenia) and 20% (France and Finland) between 2017 and 2020. The primary data source for these estimates was the WHO Mental Health Atlas (Supplement S6).^22^

For SUDs, AUDs, anxiety disorders and ADHD, no studies provided more than one estimate per country, limiting the ability to analyse trends over time.

### Sub-groups

Although subgroup-specific studies were not actively targeted in our search, several included studies focused on specific populations. These were included as they fulfilled the inclusion criteria and contributed to a broader understanding of disparities in mental health service coverage. Of the 10 studies that assessed mental health service coverage and treatment gaps among vulnerable sub-groups, most highlighted significant disparities in mental health service use. Studies focused on gender and sexual minorities,^28^ ethnic minorities and refugees,^27^^,^^36^ internally displaced and homeless people,^26^^,^^33^^,^^48^ women with specific vulnerabilities,^24^^,^^60^ and professional and student populations.^31^^,^^54^

A cross-sectional study in Czechia focusing on gender and sexual minorities showed a substantial treatment gap among people meeting the criteria for any mental health condition, with 81.13% of sexual minorities and 82.91% of heterosexual individuals remaining untreated. Among those with MDD, the gap was notably wider among sexual minorities (82.35%) compared to heterosexual individuals (60.87%).^28^

Severe treatment gaps were observed among marginalised and displaced populations. In the Paris area, only 3% of French-speaking homeless people with psychotic disorders received treatment, suggesting significant barriers to sustained care.^48^ Similarly, a cross-sectional study among 420 Syrian refugees in Ankara (Turkey) found that just 9.7% of those who felt they needed help accessed mental health services.^36^ In Ukraine, a study assessing internally displaced individuals reported a treatment gap of 74% for PTSD, 69% for MDD, and 68% for anxiety disorders, with an overall treatment gap of 74% (95% UI: 70.99–77.55).^26^ In Georgia, a study assessing internally displaced individuals with anxiety disorders in a conflict-affected zone found a treatment gap of 21.6%.^33^ In contrast, a study in Belgium of ethnic minority and low-income people with MDD, anxiety disorders and AUDs found that 47.6% had their mental health needs met clinically, leaving over half of the population untreated.^27^

Access to mental health care varied among women with specific vulnerabilities. In Portugal, women who survived intimate partner violence between 2008 and 2010 had a low treatment gap, with 46% receiving specialised mental health care, 42% consulting psychiatrists, 61% receiving care from general practitioners and 71% consulting other health professionals.^60^ In contrast, women on maternity or parental leave in Czechia with general mental health difficulties, anxiety symptoms, and alcohol use had a high treatment gap of 76% (95% UI: 55–89), with specific gaps of 78% for AUD, 64% for agoraphobia, and 60% for those experiencing suicidal thoughts.^24^

Findings from professional and student populations also showed gaps in service use. A web-based survey among first-year Spanish university students found that only 12.6% of those with possible major depressive episode, mania/hypomania, or generalised anxiety disorder received treatment.^54^ In Spain, Mortier and colleagues conducted a prospective cohort study on healthcare workers with suicidal thoughts during the COVID-19 pandemic (May 2020–September 2021), showing an increase in service use from 18.2% to 29.6% over 16 months for those with MDD, anxiety disorders, PTSD, or SUD.^31^ Women and those working longer hours were less likely to seek help, while those with a history of mental health treatment or medication use before the pandemic were more likely to use services.^31^

## Discussion

This systematic review demonstrates the scarcity of comprehensive and systematically collected data on mental health treatment coverage and gaps in the WHO European Region. Many countries lack national-level estimates, and where data do exist, they are often fragmented, inconsistent, or infrequently collected, making it very difficult to track changes over time. This lack of systematic monitoring severely limits the ability to assess progress, compare coverage across countries, and evaluate the impact of mental health policies. Despite these caveats, the available evidence highlights substantial variability in mental health treatment coverage across European countries and diagnoses. While some nations have achieved relatively high coverage for severe conditions such as psychotic disorders, significant gaps persist for common mental disorders and SUDs. In terms of temporal trends, this review is the first attempt to explore treatment coverage over time in a comparable way. However, very few studies have tracked trends systematically. Limited longitudinal data suggest that treatment coverage for MDD has remained relatively stable, with only minor fluctuations. For psychosis, there was greater variation, although differences in how countries estimated coverage may have affected the reliability of temporal comparisons. Moreover, marginalised populations continue to face disproportionately low access to mental health services, exacerbating existing health inequities.

We observed large variations in psychosis service coverage, ranging from 20% to 99% in 2017 and 2020. Some findings, such as the low coverage for psychosis in the Netherlands, warrant careful interpretation. These variations in psychotic disorder coverage likely reflect differences in healthcare infrastructure, funding, and service organisation.^65^ In the European Region, mental health systems have historically prioritised severe mental illnesses, such as psychotic disorders, over common mental disorders, which may explain the relatively better coverage for psychosis.^66^^,^^67^ However, the WHO Mental Health Atlas—the primary data source for psychosis coverage—estimates service availability rather than treatment adequacy, meaning that high coverage rates do not necessarily translate into high quality care.^22^ Conversely, for MDD, MAT estimates were lower, with some countries, such as Bulgaria and Turkmenistan, reporting MAT levels below 10% despite the higher prevalence of MDD. These disparities likely stem from differences in healthcare accessibility, cultural attitudes, and the availability of specialised care.^68^ Similar factors may also influence treatment provision for anxiety disorders. For other mental health conditions, coverage data were available for only a few countries.

Coverage data appear to suggest that marginalised populations, including ethnic minorities, refugees, individuals experiencing homelessness, and people with low socioeconomic status, face additional barriers to accessing mental health care. Structural inequalities, discrimination, stigma, and language barriers can further limit their ability to seek and receive appropriate treatment.^69^ Additionally, mental health services in many European countries may not be adequately designed to meet the specific needs of these groups, leading to persistent disparities in mental health care.

Several methodological limitations should be considered when interpreting our findings. First, differences in data sources and study designs introduced heterogeneity, limiting direct comparability across countries and diagnoses. Additionally, variations in sampling methods, diagnostic criteria, and health care system structures across studies may have influenced the reported estimates. In particular, countries with established psychiatric epidemiology and regular national surveys, such as the Netherlands, can provide more accurate and up-to-date estimates of mental health needs, while many others lack reliable prevalence data or systematic monitoring. These regional differences in diagnostic practices and data infrastructure hinder the accurate assessment and comparison of treatment needs and service coverage across Europe.

In some studies, reliance on administrative data and self-reported surveys introduced potential biases, such as under-reporting due to stigma or limited health care access. Furthermore, the lack of data for certain countries and diagnoses restricts the generalizability of the findings, particularly for SUDs and ADHD, where coverage estimates were sparse.

For ADHD, most available estimates were outdated and collected before the condition gained wider clinical recognition in adults, which likely contributed to the very low coverage levels reported. There is a disproportionate representation of studies from high-income countries (HICs), at 85%, compared to the actual distribution of income classifications within the WHO European Region. While this partially reflects the regional economic structure, where approximately 68% of countries are classified as HICs, the underrepresentation of upper-middle-income and lower-middle-income countries (which constitute 26% and 6% of the region, respectively) may limit the generalizability of findings to lower-resource settings. This imbalance likely stems from differences in research infrastructure, funding availability, and publication output across income levels. As a result, the applicability of the evidence to middle-income contexts should be interpreted with caution. Additionally, the cross-sectional nature of most studies also limits conclusions about trends over time, as fluctuations in coverage may reflect changes in data collection rather than actual shifts in service access. Additionally, while some studies distinguished between receiving any mental health treatment and receiving adequate care, assessments of care quality were generally lacking, and digital interventions were rarely specified. This limits the ability to determine whether high coverage rates reflect effective or contemporary models of treatment. Moreover, the definition of “any mental health treatment” was not standardized across studies, and we did not extract detailed information on the specific types or thresholds of care (e.g., counselling, psychotherapy, inpatient treatment). As a result, direct comparisons across countries or conditions should be interpreted with caution. Furthermore, few studies explicitly referred to common international guidelines, such as those from WHO, making it difficult to assess consistency in the standards of treatment reported.

Finally, due to these limitations, this review employed a narrative synthesis approach. Narrative synthesis allowed us to systematically summarize and interpret the diverse evidence without performing a quantitative meta-analysis. As such, we did not apply the GRADE framework to assess the certainty of evidence, since GRADE is primarily designed for reviews synthesizing effect estimates to support recommendations. Instead, we critically appraised the risk of bias of individual studies using RoB-PrevMH tool, and considered study quality and methodological limitations when interpreting findings. Future research with more homogeneous data may enable formal certainty assessments and stronger evidence grading.

In terms of the scope of our search, although we did not restrict results to English-language reports, we acknowledge that some eligible reports may have been published on institutional government websites, which we did not search for mental health coverage data. However, we deliberately searched for published scientific studies to ensure methodological transparency and replicability. National and governmental data sources are often heterogeneous in terms of reporting standards, scope, and methodological rigor, making it difficult to ensure comparability across countries. Despite these limitations, existing studies indicate that mental health service coverage in the European Region remains sub-optimal, with large service gaps. Countries should implement recording systems to monitor service coverage and minimally adequate treatment over time. While ad hoc surveys can provide detailed insights into coverage for specific areas, settings, populations, and years, establishing permanent monitoring infrastructures is essential for tracking trends consistently using standardised methodological approaches. To support better monitoring of mental health service coverage across countries, a core set of standardised definitions and indicators is needed. “Treatment coverage” should be clearly defined to include both contact coverage (i.e., any contact with a provider) and adequate coverage (i.e., care meeting minimum adequacy standards based on frequency, duration, and provider type). Variables such as diagnosis, service type, and intensity should be harmonised across studies, ideally following international guidelines (e.g., WHO Mental Health Atlas or World Mental Health Surveys). Establishing consensus on these dimensions would help improve comparability, guide resource allocation, and facilitate regional monitoring over time. This would address the current lack of data in many countries, enabling cross-country comparisons and long-term trend analysis. Additionally, systematic monitoring would allow countries to record progress toward the WHO Comprehensive Mental Health Action Plan 2013–2030 target of a 50% increase in service coverage for psychosis and MDD.^17^

The results of monitoring exercises would enable countries to plan concrete and specific actions to enhance access to mental health services. Considering the supply side (provision of services) and the demand side (uptake of services by individuals)^11^^,^^70^, ^71^, ^72^ of mental health care, access can be broken down into availability, acceptability, affordability, and the appropriateness and effectiveness of delivery.^71^^,^^72^ To achieve meaningful improvements in coverage, we recommend interventions that address both sides of the access equation across these dimensions, as summarised in Panel 1.

Panel 1 **Recommendations for action on the supply side (provision of services) and the demand side (uptake of services by individuals).**

**Table:** 

	Supply-Side actions	Demand-Side actions
Availability	1 Workforce expansion (train more mental health professionals). 2 Decentralize services to rural/remote areas. 3 Integrate mental health care into primary health care systems.	1 Conduct public awareness campaigns on available services. 2 Improve health literacy through educational programs.
Acceptability	1 Develop culturally adapted interventions. 2 Launch stigma reduction initiatives. 3 Ensure user-centred service design through community feedback.	1 Engage communities in designing and implementing mental health services. 2 Normalize mental health discussions through public campaigns and peer support.
Affordability	1 Reform health financing to increase mental health budgets. 2 Integrate mental health services into Universal Health Coverage (UHC).	1 Provide subsidised or low-cost mental health services. 2 Include mental health coverage in public and private insurance schemes.
Appropriate and effective delivery	1 Implement evidence-based interventions. 2 Build ongoing capacity of mental health workers. 3 Expand digital health platforms like telemedicine. 4 Adopt a multisectoral and integrated approach	1 Ensure person-centred care with multiple treatment options. 2 Empower users to make choices 3 Promote participation, community inclusion, and legal capacity 4 Establish feedback mechanisms to improve care

On the supply side, expanding access to mental health services requires addressing shortages in professionals, facilities, and resources. Governments should implement policies to increase the number of trained providers by expanding education, offering incentives, and training general practitioners and community health workers in basic mental health care. Integrating mental health into primary care can also enhance accessibility, particularly in underserved areas, through task-shifting initiatives that enable non-specialists to deliver interventions.^17^

Decentralisation is another crucial supply-side action to increase service availability, as many services remain concentrated in urban areas. Expanding community mental health centres, deploying mobile health units, and leveraging telemedicine can improve reach.^69^ Additionally, services must be culturally sensitive to ensure acceptability. Digital innovations like telemedicine and mobile applications can extend mental health care to remote populations. Collaborating with local communities and minoritised groups can help tailor interventions to diverse cultural and social contexts.^69^

Regarding affordability, financial barriers must be tackled through health financing reforms, including increased budget allocations, integration into Universal Health Coverage, and alternative funding strategies.^7^ Countries should also adopt evidence-based interventions, invest in training, and establish monitoring systems to assess service effectiveness and increase effective delivery.^73^

On the demand side, availability refers to public awareness and understanding of accessible mental health services. Many individuals, especially those from vulnerable groups, are unaware of these services or how to access them. Governments should invest in large-scale awareness campaigns and mental health literacy programs in schools, workplaces, and communities to help people recognise symptoms and seek help.^74^, ^75^, ^76^ In addition, acceptability depends on reducing stigma and cultural barriers to care. Engaging community leaders, fostering open discussions, and promoting initiatives like awareness days, peer support, and mental health first aid training can normalise seeking mental health support.^77^

Affordability remains a key challenge. Governments should subsidise services for low-income and vulnerable groups, ensure public facilities offer low-cost care, and mandate mental health coverage in insurance plans under mental health parity laws. To ensure effective service delivery, mental health professionals should engage users in treatment decisions based on recovery-orientated principles.^69^ WHO-recommended evidence-based interventions should promote participation, inclusion, and legal capacity. Feedback mechanisms must be in place to improve service quality and responsiveness to users’ needs.

## Conclusions

Addressing persistent gaps in mental health treatment coverage across Europe requires a coordinated approach that improves both the availability of services and the conditions that enable individuals to access them, in particular allocating resources to populations with the greatest unmet need. While some progress has been made—particularly in coverage for psychotic disorders—significant disparities remain for common mental disorders. To bridge these gaps, countries should implement comprehensive recording systems to monitor service coverage and track progress toward the WHO's mental health coverage targets. Expanding and improving mental health services through policy reforms, workforce expansion, decentralised care, and financial interventions will be crucial for achieving equitable access and improving mental health outcomes across Europe.

## Additional statements

The authors alone are responsible for the views expressed in this publication and they do not necessarily represent the decisions, policy, or views of the World Health Organization. The designations employed and the presentation of the material in this publication do not imply the expression of any opinion whatsoever on the part of WHO concerning the legal status of any country, territory, city, or area or of its authorities.

## Contributors

Concept and design of the Series paper: all authors.

Methods, search, data curation and drafting of the Series paper: Gastaldon and Barbui.

Interpretation of data: all authors.

Critical revision of the manuscript for important intellectual content: all authors.

## Declaration of interests

CG, DC, LL, ZV, SEL, RK and CB have no conflict of interest. DC and LL are staff members of the World Health Organization.
