# Supplementary material for: Mental health service coverage and gaps among adults in Europe: a systematic review
Source: Lancet Reg Health Eur. 2025 Oct 6;57:101458. doi: 10.1016/j.lanepe.2025.101458 (PMC12541639; doi:10.1016/j.lanepe.2025.101458)
Supplement: Supplementary Material [file mmc1.docx]

SUPPLEMENTARY MATERIAL

Contents

[**Supplementary methods** 2](#_Toc198913347)

[**List of excluded studies with reasons** 2](#_Toc198913348)

[**Table S1. Characteristics of included studies** 17](#_Toc198913349)

[**References of included studies** 57](#_Toc198913350)

[**Table S2. Percentage of coverage for each country and each mental disorder** 61](#_Toc198913351)

[**Table S3. Number of included studies per country and corresponding World Bank 2025 income classification and geographical region** 64](#_Toc198913352)

[**Additional information on the main sources** 65](#_Toc198913353)

[**Table S4. Risk of bias of included studies according to RoB-PrevMH** 66](#_Toc198913354)

[**Figure S1. Summary of RoB-PrevMH** 75](#_Toc198913355)

[**Table S5. Trends of mental health coverage for any mental disorder over the years for countries with more than one estimate.** 76](#_Toc198913356)

[**Table S6. Trends of service coverage for psychosis over the years for countries with more than one estimate.** 76](#_Toc198913357)

[**Table S7. Trends of mental health coverage for major depressive disorder over the years for countries with more than one estimate.** 76](#_Toc198913358)

# **Supplementary methods**

If only gap estimates were available, the corresponding coverage was calculated as the inverse of the treatment gap, i.e. by subtracting treatment gap from 100 (100-gap=coverage), according to de Silva et al.

de silva, M. J., Lee, L., Fuhr, D. C., Rathod, S., Chisholm, D., Schellenberg, J., & Patel, V. (2014). Estimating the coverage of mental health programmes: A systematic review. *International Journal of Epidemiology*, *43*(2), 341–353. <https://doi.org/10.1093/ije/dyt191>

List of countries in the World Health Organization European Region:

Albania, Andorra, Armenia, Austria, Azerbaijan, Belarus, Belgium, Bosnia and Herzegovina, Bulgaria, Croatia, Cyprus, Czech Republic, Denmark, Estonia, Finland, France, Georgia, Germany, Greece, Hungary, Iceland, Ireland, Israel, Italy, Kazakhstan, Kyrgyzstan, Latvia, Lithuania, Luxembourg, Malta, Moldova, Monaco, Montenegro, Netherlands, North Macedonia, Norway, Poland, Portugal, Romania, Russia, San Marino, Serbia, Slovakia, Slovenia, Spain, Sweden, Switzerland, Tajikistan, Turkey, Turkmenistan, Ukraine, United Kingdom, Uzbekistan

# **List of excluded studies with reasons**

WRONG COUNTRY

1. Cole, F., Benjet, C., Ghimire, D., Axinn, W.G. (2021). Predictors of transitions across stages of alcohol use and disorders in an adult population with heterogeneous ethnic restrictions regarding drinking. *Addiction*, 116(4):809-818
2. Hanna Chaim, C., Santana, G.L., de Vries Albertin, P., Silveira, C.M., Siu, E.R., Viana, M.C., Pang, W.Y., Andrade, L.H. (2021). Alcohol use patterns and disorders among individuals with personality disorders in the Sao Paulo Metropolitan Area. *PLoS One*, 16(3):e0248403.
3. Yasuma, N., Nishi, D., Watanabe, K., Ishikawa, H., Tachimori, H., Takeshima, T., Umeda, M., Kawakami, N. (2021). Association between Urban Upbringing and Compulsive Internet Use in Japan: A Cross-Sectional, Multilevel Study with Retrospective Recall*International Journal of Environmental Research and Public Health*, 18(18): 9890.
4. Axinn, W.G., Zhang, Y., Ghimire, D.J., Chardoul, S.A., Scott, K.M., Bruffaerts, R. (2020). The association between marital transitions and the onset of major depressive disorder in a South Asian general population. *Journal of Affective Disorders*, 266:165-172.
5. Stagnaro, J.C., Cia, A.H., Aguilar-Gaxiola, S., Váquez, N., Sustas, S., Benjet, C., Kessler, R.C. (2018) Twelve-month prevalence rates of mental disorders and service use in the Argentinean Study of Mental Health Epidemiology. *Social Psychiatry and Psychiatric Epidemiology*, 53(2):121-129.
6. Wardenaar, K.J., Huang, Y., Wojtyniak, B., de Jonge, P. (2018). Comorbidity: The Cross-national Structure of Mental Disorders. In K.M Scott, P. de Jonge, D.J. Stein, R.C Kessler (Eds.), Mental Disorders Around the World: Facts and Figures from the WHO World Mental Health Surveys (pp. 297-313). New York: Cambridge University Press.
7. Wardenaar, K.J., Benjet, C., Viana, M.C., de Jonge, P. (2018). Specific Phobia. In K.M Scott, P. de Jonge, D.J. Stein, R.C Kessler (Eds.), Mental Disorders Around the World: Facts and Figures from the WHO World Mental Health Surveys (pp. 134-152). New York: Cambridge University Press.
8. Chiavegatto Filho, A.D., Wang, Y.P., Campino, A.C., Malik, A.M., Viana, M.C., Andrade, L.H. (2015). Incremental health expenditure and lost days of normal activity for individuals with mental disorders: Results from the São Paulo Megacity Study. *BMC Public Health*, 15, 745.
9. Aradati, M., Bilal, L., Naseem, M.T., Hyder, S., Al‐Habeeb A., AlSubaie, A., Shahab, M., Sohail, B., Baig, M., Binmuammar, A., & Altwaijri, Y. (2019). Using knowledge management tools in the Saudi National Mental Health Survey helpdesk: pre and post study. *International Journal of Mental Health Systems*, 13:33.

WRONG POPULATION

1. Ebert, D.D., Mortier, P., Kaehlke, F., Bruffaerts, R., Baumeister, H., Auerbach, R.P., Alonso, J., Vilagut, G., Martinez, K.U., Lochner, C., Cuijpers, P., Kuechler, A.M., Green, J.G., Hasking, P., Lapsley, C., Sampson, N.A., Kessler, R.C., and on behalf of WHO WMH-ICS collaborators. (2019). Barriers of mental health treatment utilization among first-year college students: First cross-national results from the WHO World Mental Health International College Student Initiative. *International Journal of Methods in Psychiatric Research*, 28(2): e1782. [PubMed Abstract](https://www.ncbi.nlm.nih.gov/pubmed/31069905) WRONG POPULATION
2. Borges, G., Orozco, R., Benjet, C., Martínez Martínez, K.I., Contreras, E.V., Jiménez Pérez, A.L., Pelaez Cedrés, A.J., Hernández Uribe, P.C., Covarrubias Díaz-Couder, M.A., Gutierrez-Garcia, R.A, Quevedo Chávez, G.E., Albor, Y., Mendez, E., Medina-Mora, M.E., Mortier, P., Ayuso-Mateos, J.L. (2021). (Internet) Gaming Disorder in DSM-5 and ICD-11: A Case of the Glass Half Empty or Half Full: (Internet) Le trouble du jeu dans le DSM-5 et la CIM-11: Un cas de verre à moitié vide et à moitié plein. *Canadian Journal of Psychiatry*, 66(5):477-484. [PubMed Abstract](https://pubmed.ncbi.nlm.nih.gov/32806957/)
3. Germanaud, E., Callahan, S., Revranche, M., Biscond, M., Pic, O., & Husky, M. (2024). Mental Disorders and Suicidality by Sexual Orientation Status Among First-Year College Students in France. *Journal of Homosexuality*, [Epub ahead of print].
4. Amanvermez, Y., Karyotaki, E., Cuijpers, P., Ciharova, M., Bruffaerts, R., Kessler, R. C., Klein, A. M., Wiers, R. W., & de Wit, L. M. (2024). Sources of stress among domestic and international students: a cross-sectional study of university students in Amsterdam, The Netherlands. Anxiety, Stress, and Coping, 1-18, 37(4), 428-445. PubMed Abstract
5. Bantjes, J., Hunt, X., Cuijpers, P., Kazdin, A.E., Kennedy, C.J., Luedtke, A., Malenica, I., Petukhova, M., Sampson, N., Zainal, N.H., Davids, C., Dunn-Coetzee, M., Gerber, R., Stein, D.J., Kessler, R.C. (2024). Comparative effectiveness of remote digital gamified and group CBT skills training interventions for anxiety and depression among college students: Results of a three-arm randomised controlled trial. *Behaviour Research and Therapy*, 178, 104554.
6. Janota, M., Pic, O., Callahan, S., & Husky, M. M. (2024) Risky Alcohol Use and Romantic Relationships Among College Students: A Longitudinal Study. *Substance Use and Misuse*, 59 (12), 1683-1694.
7. Langer, Á. I., Schmidt, C., Martínez, V., Mac-Ginty, S., Fuentes, R., & Núñez, D. (2024). Suicidal ideation, non-suicidal self-injury and psychopathology in university students: Examining the moderating role of experiential avoidance. Journal of Affective Disorders Reports, 16, 100734
8. Miranda-Mendizabal, A., Castellví, P., Vilagut, G., Alayo, I., Almenara, J., Ballester, L., Echeburúa, E., Gabilondo, A., Gili, M., Mortier, P., Piqueras, J.A., Roca, M., Auerbach, R.P., Bruffaerts, R., Kessler, R.C., Alonso, J., on behalf of the UNIVERSAL study group. (2024). Suicidal Ideation Risk among LGB Spanish University Students: The Role of Childhood and Adolescence Adversities and Mental Disorders. *Journal of Affective Disorders*, 353: 52-59
9. Benjet, C., Orozco, R., Albor, Y. C., Contreras, E. V., Monroy-Velasco, I. R., Hernández Uribe, P. C., Báez Mansur, P. M., Covarrubias Díaz Couder, M. A., Quevedo Ch´vez, G. E., Gutierrez-García, R. A., Machado, N., Andersson, C., & Borges, G. (2023). A longitudinal study on the impact of Internet gaming disorder on self-perceived health, academic performance, and social life of first-year college students. *The American Journal on Addictions*, 32(4):343-351.
10. Karam, E. G., Saab, D., Jabbour, S., Karam, G. E., Hantouche, E., & Angst, J. (2023). The role of affective temperaments in bipolar disorder: The solid role of the cyclothymic, the contentious role of the hyperthymic, and the neglected role of the irritable temperaments. *European Psychiatry: the journal of the Association of European Psychiatrists*, 66(1), e37.
11. Kiekens, G., Hasking, P., Bruffaerts, R., Alonso, J., Auerbach. R.P., Bantjes, J., Benjet, C., Boyes, M., Chiu, W.T., Claes, L., Cuijpers, P., Ebert, D.D., Mak, A., Mortier, P., O'Neill, S., Sampson, N.A., Stein, D.J., Vilagut, G., Nock, M.K., Kessler. R.C., and on behalf of the WHO World Mental Health International college student (WMH-ICS) collaborators. (2023). Non-suicidal self-injury among first-year college students and its association with mental disorders: Results from the World Mental Health International College Student (WMH-ICS) Initiative. *Psychological Medicine*,53(3), 875-886.
12. Revranche, M., Biscond, M., Navarro-Mateu, F., Kovess-Masfety, V., & Husky, M. M. (2023). The contribution of childhood adversities to the persistence of severe role impairment among college students: a follow-up study. *Social Psychiatry and Psychiatric Epidemiology*
13. Bond, N., McLafferty, M., Lapsley, C., Ennis, E., Murray, E., Heenan, D., & O'Neill, S. (2022). Familial and Bullying Victimisation: The Impact of Early Adversity Within the Home and Peer Settings on Late Adolescence and Adult Psychopathology. *Journal of Child and Adolescent Trauma*.
14. Bootsma, E., Jansen, L., Van Houte, K., Kessler, R. C., Tack, J., Bruffaerts, R., & Gut-Brain Interactions - WHO - World Mental Health International College Student Initiative Working Group (2024). Beyond the Norm: Epidemiology of Irritable Bowel Syndrome and Mental Health Among Sexual and Gender-Diverse Young Adults in a Large Representative Cohort. *Gastroenterology*, S0016-5085(24)05121-7, [Epub ahead of print]. [PubMed Abstract](https://pubmed.ncbi.nlm.nih.gov/38944208/)
15. Bond, N., McLafferty, M., Lapsley, C., Ennis, E., Murray, E., Heenan, D., & O'Neill, S. (2022). Familial and Bullying Victimisation: The Impact of Early Adversity Within the Home and Peer Settings on Late Adolescence and Adult Psychopathology. *Journal of Child and Adolescent Trauma*

WRONG STUDY TYPE

1. Slade, T., Degenhardt, L., Wang, P. S., Tohen, M., Bromet, E. J., Angst, J. and Kessler, R. C. (2015) Psychiatric Epidemiology. In Tasman, A., Kay, J., Lieberman, A., First, M.B., Riba, M.B. (Eds.)*Psychiatry*, Fourth Edition. Chichester, UK: John Wiley & Sons, Ltd.
2. Ustun, B., Adler, L.A., Rudin, C., Faraone, S.V., Spencer, T.J., Berglund, P., Gruber, M.J., Kessler, R.C. (2017). The World Health Organization Adult Attention-Deficit/Hyperactivity Disorder Self-Report Screening Scale for DSM-5. *JAMA Psychiatry*, 74(5), 520-526
3. Adler, L.A., Faraone, S.V., Spencer, T.J., Berglund, P., Alperin, S., Kessler, R.C. (2017). The structure of adult ADHD. *International Journal of Methods in Psychiatric Research*, 26(1), e1555.
4. Roest, A.M., de Jonge, P., Lim, C.C.W., Stein, D.J., Medina-Mora, M.E., Scott, K.M. (2018). Agoraphobia. In K.M Scott, P. de Jonge, D.J. Stein, R.C Kessler (Eds.), Mental Disorders Around the World: Facts and Figures from the WHO World Mental Health Surveys (pp. 106-119). New York: Cambridge University Press.
5. Scott, K.M., de Jonge, P., Stein, D.J., Kessler, R.C. (2018). Introduction. In K.M Scott, P. de Jonge, D.J. Stein, R.C Kessler (Eds.), Mental Disorders Around the World: Facts and Figures from the WHO World Mental Health Surveys (pp. 324-336). New York: Cambridge University Press.
6. Scott, K.M., Stein, D.J., de Jonge, P., Kessler, R.C. (2018). Discussion. In K.M Scott, P. de Jonge, D.J. Stein, R.C Kessler (Eds.), Mental Disorders Around the World: Facts and Figures from the WHO World Mental Health Surveys (pp. 324-336). New York: Cambridge University Press.
7. Degenhardt, L., Torres, Y., Hinkov, H., ten Have, M., Glantz, M.D. (2018). Drug-Use Disorders. In K.M Scott, P. de Jonge, D.J. Stein, R.C Kessler (Eds.),. Mental Disorders Around the World: Facts and Figures from the WHO World Mental Health Surveys (pp. 243-262). New York: Cambridge University Press.
8. Evans-Lacko, S., Tachimori, H., Kovess-Masfety, V., Chatterji, S., Thornicroft, G. (2018). Service Use. In K.M Scott, P. de Jonge, D.J. Stein, R.C Kessler (Eds.), Mental Disorders Around the World: Facts and Figures from the WHO World Mental Health Surveys (pp. 314-323). New York: Cambridge University Press.
9. Evans-Lacko, S., Aguilar-Gaxiola, S., Al-Hamzawi, A., Alonso, J., Benjet, C. , Bruffaerts, R., Chiu, W.T., Florescu, S., de Girolamo, G., Gureje, O., Haro, J. M., He, Y., Hu, C., Karam , E. G., Kawakami, N., Lee, S., Lund, C., Kovess-Masfety, V., Levinson, D., Navarro-Mateu, F., Pennell, B.E., Sampson, N.A., Scott, K.M., Tachimori, H., ten Have, M., Viana, M.C., Williams, D.R., Wojtyniak, B.J., Zarkov, Z. , Kessler, R.C., Chatterji, S., Thornicroft, G. (2018). Socio-economic variations in the mental health treatment gap for people with anxiety, mood, and substance use disorders: Results from the WHO World Mental Health (WMH) Surveys.*Psychological Medicine*, 48(9):1560-1571.
10. Evans-Lacko, S., Thornicroft, G. (2019). Viewpoint: WHO World Mental Health Surveys International College Student initiative: implementation issues in low- and middle-income countries. *International Journal of Methods in Psychiatric Research*, 28(2):e1756.
11. Ebert, D.D., Franke, M., Kählke, F., Küchler, A.M., Bruffaerts, R., Mortier, P., Karyotaki, E., Alonso, J., Cuijpers, P., Berking, M., Auerbach, R.P., Kessler, R.C., Baumeister, H. (2019). Increasing intentions to use mental health services among university students. Results of a pilot randomized controlled trial within the WHO World Mental Health International College Student Initiative. *International Journal of Methods in Psychiatric Research*, 28(2):e1754.
12. Ebert, D.D., Buntrock, C., Mortier, P., Auerbach, R., Weisel, K.K., Kessler, R.C., Cuijpers, P., Green, J.G., Kiekens, G., Nock, M.K, Demyttenaere, K., Bruffaerts, R. (2019). Prediction of major depressive disorder onset in college students. *Depression and Anxiety*, 36(4), 294-304. [P](https://www.ncbi.nlm.nih.gov/pubmed/30521136)
13. Silva, D. A., Coutinho, E. D. S. F., Ferriani, L. O., & Viana, M. C. (2020). Depression subtypes and obesity in adults: A systematic review and meta-analysis. *Obesity Reviews: An official journal of the International Association for the Study of Obesity*, 21(3), e12966.
14. Mortier, P., Vilagut, G., Puértolas Gracia, B., De Inés Trujillo, A., Alayo Bueno, I., Ballester Coma, L., Blasco Cubedo, M.J., Cardoner, N., Colls, C., Elices, M., García Altés, A., GenéBadia, M., Gómez Sánchez, J., Martín Sánchez, M., Morros Pedrós, R., Prat Pubill, B., Qin, P., Kessler, R.C., Palao, D., Pérez, V., Alonso, J., on behalf of the CODIRISC Epidemiology Study Group. (2020). The Catalonia Suicide Risk Code Epidemiology (CSRC-Epi) Study: protocol for a population-representative nested case-control study of suicide attempts in Catalonia, Spain. *BMJ Open*, 10(7):e037365
15. Scott, K.M., de Vries, Y.A., Aguilar-Gaxiola, S., Al-Hamzawi, A., Alonso, J., Bromet, E.B., Bunting, B. Caldas-de-Almeida, J.M., Cia, A., Florescu, S., Gureje, O., Hu, C-Y., Karam, E.G., Karam, A., Kawakami, N., Kessler, R.C., Lee, S., McGrath, J., Oladeji, B., Posada-Villa, J., Stein, D.J., Zarkov, Z., de Jonge, P. (2020). Intermittent Explosive Disorder subtypes in the general population: association with comorbidity, impairment and suicidality. *Epidemiology and Psychiatric Sciences*, 29:e138.
16. Harrer, M., Adam, S.H., Rathner, E.M., Baumeister, H., Cuijpers, P., Bruffaerts, R., Auerbach, R.P., Kessler, R.C., Jacobi, C., Taylor, C.B., Ebert, D.D. (2020). Prevention of Eating Disorders at Universities: A Systematic Review and Meta-Analysis. *International Journal of Eating Disorders*, 53(6):813-833.
17. Amanvermez, Y., Rahmadiana, M., Karyotaki, E., de Wit, L., Ebert, D.D., Kessler, R.C., Cuijpers, P. (2020). Stress management interventions for college students: A systematic review and meta-analysis. *Clinical Psychology: Science and Practice*, 00: e12342
18. Coêlho, B.M., Santana, G.L., Viana, M.C., Wang, Y.P., Andrade, L.H. (2021). "I don't need any treatment" - barriers to mental health treatment in the general population of a megacity. *Brazilian Journal of Psychiatry*, 43(6):590-598
19. Cuijpers, P., Miguel, C., Ciharova, M., Aalten, P., Batelaan, N., Salemink, E., Spinhoven, P., Struijs, S., de Wit, L., Gentili, C., Ebert, D., Harrer, M., Bruffaerts, R., Kessler, R.C., Karyotaki, E. (2021). Prevention and treatment of mental health and psychosocial problems in college students: An umbrella review of meta-analyses. *Clinical Psychology: Science and Practice*, 28(3), 229-244.
20. Karyotaki, E., Klein, A.M., Ciharova, M., Bolinski, F., Krijnen, L., de Koning, L., de Wit, L., van der Heijde, C.M., Ebert, D.D., Riper, H., Batelaan, N., Vonk, P., Auerbach, R.P., Kessler, R.C., Bruffaerts, R., Struijs, S., Wiers, R. W., & Cuijpers, P. (2022). Guided internet-based transdiagnostic individually tailored Cognitive Behavioral Therapy for symptoms of depression and/or anxiety in college students: A randomized controlled trial. *Behaviour Research and Therapy*, 150, 104028
21. Saruhanjan, K., Zarksi, A.C., Bauer, T., Baumeister, H., Cuijpers, P., Spiegelhalder, K., Auerbach, R.P., Kessler, R.C., Bruffaerts, R., Karyotaki, E., Berking, M., Ebert, D.D. (2021). Psychological interventions to improve sleep in college students: A meta-analysis of randomized controlled trials. *Journal of Sleep Research*, 30(1):e13097.
22. Navarro-Mateu, F., Husky, M., Cayuela-Fuentes, P., Álvarez, F.J., Roca-Vega, A., Rubio-Aparicio, M., Chirlaque, M.D., Cayuela, M.L., Martínez, S., Sánchez-Meca, J. (2021). The association of telomere length with substance use disorders: a systematic review and meta-analysis of observational studies. *Addiction*, 116(8):1954-1972
23. Bharat, C., Glantz, M. D., Aguilar-Gaxiola, S., Alonso, J., Bruffaerts, R., Bunting, B., Caldas-de-Almeida, J. M., Cardoso, G., Chardoul, S., de Jonge, P., Gureje, O., Haro, J. M., Harris, M. G., Karam, E. G., Kawakami, N., Kiejna, A., Kovess-Masfety, V., Lee, S., McGrath, J. J., Moskalewicz, J., Navarro-Mateu, F., Rapsey, C., Sampson, N.A., Scott, K.M., Tachimori, H., Have, M.T., Vilagut, G., Wojtyniak, B., Xavier, M., Kessler, R.C., Degenhardt, L. (2023). Development and evaluation of a risk algorithm predicting alcohol dependence after early onset of regular alcohol use. *Addiction*, 118(5), 954-966
24. Berman, A.H., Topooco, N., Lindfors, P., Bendtsen, M., Lindner, P., Molander, O., Kraepelien, M., Sundström, C., Talebizadeh, N. Engström, K., Vlaescu, G., Andersson, G., Andersson, C. (2024). Transdiagnostic and tailored internet intervention to improve mental health among university students: Research protocol for a randomized controlled trial. *Trials*, 25, 158.
25. Vereschagin, M., Wang, A. Y., Richardson, C. G., Xie, H., Munthali, R. J., Hudec, K. L., Leung, C., Wojcik, K. D., Munro, L., Halli, P., Kessler, R. C., & Vigo, D. V. (2024). Effectiveness of the Minder Mobile Mental Health and Substance Use Intervention for University Students: Randomized Controlled Trial. *Journal of Medical Internet Research*, 26, e54287.
26. Benjet, C., Zainal, N. H., Albor, Y., Alvis-Barranco, L., Carrasco-Tapias, N., Contreras-Ibáñez, C. C., Cudris-Torres, L., de la Peña, F. R., González, N., Guerrero-López, J. B., Gutierrez-Garcia, R. A., Jiménez-Peréz, A. L., Medina-Mora, M. E., Patiño, P., Cuijpers, P., Gildea, S. M., Kazdin, A. E., Kennedy, C. J., Luedtke, A., Sampson, N. A., Petukhova, M. V., Kessler, R. C. (2023). A Precision Treatment Model for Internet-Delivered Cognitive Behavioral Therapy for Anxiety and Depression Among University Students: A Secondary Analysis of a Randomized Clinical Trial. *JAMA Psychiatry*, 80(8), 768-777.
27. Wang, A. Y., Vereschagin, M., Richardson, C. G., Xie, H., Hudec, K. L., Munthali, R. J., Munro, L., Leung, C., Kessler, R. C., & Vigo, D. V. (2023). Evaluating the Effectiveness of a Codeveloped e-Mental Health Intervention for University Students: Protocol for a study.
28. Leung, C., Pei, J., Hudec, K., Shams, F., Munthali, R., Vigo, D. (2022). The Effects of Nonclinician Guidance on Effectiveness and Process Outcomes in Digital Mental Health Interventions: Systematic Review and Meta-analysis. *Journal of Medical Internet Research*, 24(6):e36004.
29. Silove, D., He, Y., Piazza Ferrand, M., Scott, K.M. (2018). Separation Anxiety Disorder. In K.M Scott, P. de Jonge, D.J. Stein, R.C Kessler (Eds.), Mental Disorders Around the World: Facts and Figures from the WHO World Mental Health Surveys (pp. 167-181). New York: Cambridge University Press
30. Turner, B.J., Hu, C., Posada-Villa, J., Nock, M.K. (2018). Oppositional Defiant Disorder and Conduct Disorder. In K.M Scott, P. de Jonge, D.J. Stein, R.C Kessler (Eds.), Mental Disorders Around the World: Facts and Figures from the WHO World Mental Health Surveys (pp. 209-222). New York: Cambridge University Press.
31. Stein, D.J., de Jonge, P., Kessler, R.C., Scott, K.M. (2018). The Cross-National Epidemiology of Mental Disorders. In K.M Scott, P. de Jonge, D.J. Stein, R.C Kessler (Eds.), Mental Disorders Around the World: Facts and Figures from the WHO World Mental Health Surveys (pp. 3-8). New York: Cambridge University Press.
32. Axinn, W.G., Scott, K.M., Chardoul, S.A. (2015). The Demography of Mental Health. In: Howard Friedman, (Ed.) *Encyclopedia of Mental Health, Second edition* (18-25) New York: Academic Press.
33. Bantjes, J., Kazdin, A.E., Cuijpers, P., Breet, E., Dunn-Coetzee, M., Davids, C., Stein, D.J., Kessler, R.C. (2021). A Web-Based Group Cognitive Behavioral Therapy Intervention for Symptoms of Anxiety and Depression Among University Students: Open-Label, Pragmatic Trial. *JMIR Mental Health*, 8(5), e27400.
34. Bruffaerts, R., Voorspoels, W., Jansen, L., Kessler, R.C., Mortier, P., Vilagut, G., de Vocht, J., Alonso, J, for the RECOVID consortium. (2021). Suicidality among healthcare professionals during the first COVID19 wave. *Journal of Affective Disorders*, 283: 66-70.
35. Bharat, C., Glantz, M. D., Aguilar-Gaxiola, S., Alonso, J., Bruffaerts, R., Bunting, B., Caldas-de-Almeida, J. M., Cardoso, G., Chardoul, S., de Jonge, P., Gureje, O., Haro, J. M., Harris, M. G., Karam, E. G., Kawakami, N., Kiejna, A., Kovess-Masfety, V., Lee, S., McGrath, J. J., Moskalewicz, J., Navarro-Mateu, F., Rapsey, C., Sampson, N.A., Scott, K.M., Tachimori, H., Have, M.T., Vilagut, G., Wojtyniak, B., Xavier, M., Kessler, R.C., Degenhardt, L. (2023). Development and evaluation of a risk algorithm predicting alcohol dependence after early onset of regular alcohol use. Addiction, 118(5), 954-966. PubMed Abstract

WRONG OUTCOME

1. Bunting, B., Bharat, C. (2019). Alcohol use, regular use, disorder and remission from use disorders in Northern Ireland: a prevalence study. *Addiction Research & Theory*, 27(4): 347-353.
2. Cuijpers, P., Auerbach, R.P., Benjet, C., Bruffaerts, R., Ebert, D.D., Karyotaki, E., Kessler, R.C. (2019). Introduction to the Special Issue: The WHO World Mental Health International College Student (WMH-ICS) initiative. *International Journal of Methods in Psychiatric Research*, 28(2):e1762.
3. Cuijpers, P., Auerbach, R.P., Benjet, C., Bruffaerts, R., Ebert, D.D., Karyotaki, E., Kessler, R.C. (2019). The WHO World Mental Health International College Student (WMH-ICS) initiative: An overview. *International Journal of Methods in Psychiatric Research*, 28(2): e1761
4. Degenhardt, L., Bharat, C., Glantz, M.D., Sampson, N.A., Al-Hamzawi, A., Alonso, J., Andrade, L.H., Bunting, B., Cia, A., de Girolamo, G., De Jonge, P., Demyttenaere, K., Gureje, O., Haro, J.M., Harris, M.G., He, Y., Hinkov, H., Karam, A.N., Karam, E.G., Kiejna, A., Kovess-Masfety, V., Lasebikan,V., Lee, S., Levinson, D., Medina-Mora, M.E., Mneimneh, Z., Navarro-Mateu, F., Piazza, M., Posada-Villa, J., Scott, K., Stein, D.J., Tachimori, H., Tintle, N., Torres, Y., Kessler, R.C., and on behalf of the WHO World Mental Health Survey Collaborators (2019). Association of Cohort and Individual Substance Use with Risk of Transitioning to Drug Use, Drug Use Disorder, and Remission from Disorder: Findings From the World Mental Health Surveys. *JAMA Psychiatry*, 76(7): 708-720.
5. Degenhardt, L., Bharat, C., Bruno, R., Glantz, M.D., Sampson, N.A., Aguilar‐Gaxiola, S., Alonso, J., Andrade, L.H., Bunting, B., Caldas‐de‐Almeida, J.M., Cia, A.H., Gureje, O., Karam, E.G., Khalaf, M., McGrath, J., Moskalewicz, J., Lee, S., Mneimneh, Z., Navarro‐Mateu, F., Sasu, C.C., Scott, K., Torres, Y., Poznyak, V., Chatterji, S., Kessler, R.C., and on behalf of the WHO World Mental Health Survey Collaborators. (2019). Concordance between the diagnostic guidelines for alcohol and cannabis use disorders in the draft ICD‐11 and other classification systems: Analysis of data from the WHO's World Mental Health Surveys. *Addiction*, 114(3), 534-552.
6. Degenhardt, L., Bharat, C., Glantz, M.D., Sampson, N.A., Scott, K., Lim, C.C.W., Aguilar-Gaxiola, S., Al-Hamzawi, A., Alonso, J., Andrade, L.A., Bromet, E.J., Bruffaerts, R., Bunting, B., de Girolamo, G., Gureje, O., Haro, J.M., Harris, M., He, Y., de Jonge P., Karam, E.G., Karam, G.E., Kiejna, A., Lee, S., Lepine, J.P., Levinson, D., Makanjuola, V., Medina-Mora, M.E., Mneimneh, Z., Navarro-Mateu, F., Posada-Villa, J., Stein, D.J., Tachimori, H., Torres, Y., Zarkov, Z., Chatterji, S., Kessler R.C., and on behalf of the WHO World Mental Health Survey Collaborators. (2019). The epidemiology of drug use disorders cross-nationally: Findings from the WHO’s World Mental Health Surveys. *International Journal of Drug Policy*, 71:103-112.
7. Del Valle Tena, O., Benjet, C, Medina-Mora, ME, Borges, G, Wagner, F.A. (2019) Chronic childhood adversity and speed of transition through stages of alcohol involvement. *Drug and Alcohol Dependence*, 205:107669.
8. de Vries, Y.A., Al-Hamzawi, A., Alonso, J., Borges, G., Bruffaerts, R., Bunting, B., Caldas-de-Almeida, J.M., Cia, A.H., de Girolamo, G., Dinolova, R., Esan, O., Florescu, S., Gureje, O., Haro, J.M., Hu, C., Karam, E.G., Karam, A., Kawakami, N., Kiejna, A., Kovess-Masfety, V., Lee, S., Mneimneh, Z., Navarro-Mateu, F., Piazza, M., Scott, K., Ten Have, M., Torres, Y., Viana, M.C., Kessler, R.C., de Jonge, P., and on behalf of WHO World Mental Health Survey collaborators. (2019). Childhood generalized specific phobia as an early marker of internalizing psychopathology across the lifespan: results from the World Mental Health Surveys. *BMC Medicine*, 17(1):101.
9. Stokes, C.M., Alonso, J., Andrade, L.H., Atwoli, L., Cardoso, G., Chiu, W.T., Dinolova, R.M., Dinolova, R., Gureje, O., Karam, A.N., Karam, E.G., Kessler, R.C., Chatterji. S., King, A., Lee, S., Mneimneh, Z., Oladeji, B., Petukhova, M., Rapsey, C., Sampson, N.A., Scott, K., Street, A., Viana, M.C., Williams, M.A., Bossarte, R., on behalf of the WHO World Mental Health Survey Collaborators. (2020). Pre-marital predictors of marital violence in the WHO World Mental Health (WMH) Surveys. *Social Psychiatry and Psychiatric Epidemiology*, 55 (3), 393-405.
10. Wilks, C.R., Auerbach, R.P., Alonso, J., Benjet, C., Bruffaerts, R., Cuijpers, P., Ebert D.D., Green, J.G., Mellins, C.A., Mortier, P., Sadikova E., Sampson, N.A., Kessler, R.C. (2020). The importance of physical and mental health in explaining health-related academic role impairment among college students. *Journal of Psychiatric Research*, 123, 54-61.
11. Alonso, J., Vilagut, G., Mortier, P., Auerbach, R.P., Bruffaerts, R., Cujipers, P., Demyttenaere, K., Ebert, D.D., Ennis, E., Gutiérrez-García, R.A., Greif Green, J., Hasking, P., Lee, S., Bantjes, J., Nock, M.K., Pinder-Amaker, S., Sampson, N.A., Zaslavsky, A.M., Kessler, R.C., on behalf of the WHO WMH-ICS Collaborators (2019). The role impairment associated with mental disorder risk profiles in the WHO World Mental Health International College Student Initiative. *International Journal of Methods in Psychiatric Research*, 28(2):e1750.
12. Angst, J., Rössler, W., Ajdacic-Gross, V., Angst, F., Wittchen, H.U., Lieb, R., Beesdo-Baum, K., Asselmann, E., Merikangas, K.R., Cui, L., Andrade, L.H., Viana, M.C., Lamers, F., Penninx, B.W., de Azevedo Cardoso, T., Jansen, K., Dias de Mattos Souza, L., Azevedo da Silva, R., Kapczinski, F., Grobler, C., Gholam-Rezaee, M., Preisig, M., Vandeleur, C.L. (2019). Differences between unipolar mania and bipolar-I disorder: Evidence from nine epidemiological studies. *Bipolar Disorders*, 21 (5): 437-448.
13. Antunes, A., Frasquilho, D., Azeredo-Lopes, S., Silva, M., Cardoso, G., Caldas-de-Almeida, J.M. (2019). Changes in socioeconomic position among individuals with mental disorders during the Economic Recession in Portugal: A follow-up of the National Mental Health Study. *Epidemiology and Psychiatric Sciences*, 28(6), 638-643
14. Auerbach, R.P., Mortier, P., Bruffaerts, R., Alonso, J., Benjet, C., Cujipers, P., Demyttenaere, K., Ebert, D., Green, J.G., Hasking, P., Lee, S., Lochner, C., McLafferty, M., Nock, M.K., Petukhova, M., Pinder-Amaker, S., Rosellini, A.J., Sampson, N.A., Vilagut G., Zaslavsky, A.M., Kessler, R.C., and on behalf of the WHO WMH-ICS Collaborators. (2019). Mental disorder comorbidity and suicidal thoughts and behaviors in the World Health Organization World Mental Health Surveys International College Student initiative. *International Journal of Methods in Psychiatric Research*, 28(2):e1752. [P](https://www.ncbi.nlm.nih.gov/pubmed/30450753)
15. Ballester, L., Alayo, I., Vilagut, G., Almenara, J., Cebrià, A.I., Echeburúa, E., Gabilondo, A., Gili, M., Lagares, C., Piqueras, J.A., Roca, M., Soto-Sanz, V., Blasco, M.J., Castellví, P., Forero, C.G., Bruffaerts, R., Mortier, P. Auerbach, R.P., Nock, M.K., Sampson, N.A., Kessler, R.C., and Alonso, J. on behalf of the UNIVERSAL study group. (2019). Accuracy of online survey assessment of mental disorders and suicidal thoughts and behaviors in Spanish university students. Results of the WHO World Mental Health- International College Student initiative. *PLoS One*, 14(9): e0221529.
16. Blasco, M.J., Vilagut, G., Almenara, J., Roca, M., Piqueras, J.A., Gabilondo, A., Lagares, C., Soto-Sanz, V., Alayo, I., Forero, C.G., Echeburúa, E., Gili, M., Cebrià, A.I., Bruffaerts, R., Auerbach, R.P., Nock, M.K., Kessler, R.C., Alonso, J., UNIVERSAL study group. (2019). Suicidal Thoughts and Behaviors: Prevalence and Association with Distal and Proximal Factors in Spanish University Students. *Suicide and Life Threatening Behavior*, 256: 192-204.
17. Bruffaerts, R., Mortier, P., Auerbach, R.P., Alonso, J., De la Torre, A.E.H., Cuijpers, P., Demyttenaere, K., Ebert, D.D., Green, J.G., Hasking, P., Stein, D.J., Ennis, E., Nock, M.K., Pinder-Amaker, S., Sampson, N.A., Vilagut, G., Zaslavsky, A.M., Kessler, R.C. and on behalf of the WHO WMH-ICS Collaborators. (2019). Lifetime and 12-month treatment for mental disorders and suicidal thoughts and behaviors among first year college students. *International Journal of Methods in Psychiatric Research*, 28(2):e1764.
18. Serra, R., Kiekens, G., Vanderlinden, J., Vrieze, E., Auerbach, R.P., Claes, L., Cuijpers, P., Demyttenaere, K., Ebert, D.D.., Tarsitani, L., Green, J.G., Kessler, R.C., Nock, M.K., Mortier, P., Bruffaerts, R. (2020). Binge eating and purging in first-year college students: Prevalence, psychiatric comorbidity, and academic performance. *International Journal of Eating Disorders*, 53(3), 339-348.
19. Silva, M., Antunes, A., Azeredo-Lopes, S., Cardoso, G., Xavier, M., Saraceno, B., Caldas de Almeida, J.M. (2020). How did the use of psychotropic drugs change during the Great Recession in Portugal? A follow-up to the National Mental Health Survey. *BMC Psychiatry*, 20(1):215.
20. Axinn, W.G., Chardoul, S., Gatny, H., Ghimire, D.J., Smoller, J.W., Zhang, Y., Scott, K.M. (2020). Using life history calendars to improve measurement of lifetime experience with mental disorders. *Psychological Medicine*, 50(3), 515-522.
21. Benjet, C., Axinn, W., Hermosilla, S., Schulz, P., Cole, F., Sampson, L., Ghimire, D. (2020). Exposure to armed conflict in childhood versus older ages and subsequent onset of major depressive disorder. *Jama Network Open*,3(11): e2019848.
22. Cardoso, G., Antunes, A., Silva, M., Azeredo-Lopes, S., Xavier, M., Koenen, K., Caldas-de-Almeida, J.M. (2020). Trauma exposure and PTSD in Portugal: Findings from the WHO World Mental Health Survey Initiative. *Psychiatry Research*, 284:112644.
23. Fowler, C., Homandberg, L., Steele, C., Bolt, M.A., Tintle N., Van de Griend, K., Ulroch, R., Christians, M. (2020). Adult correlates of adverse childhood experiences in Ukraine. *Child Abuse & Neglect*, 107:104617.
24. Glantz, M.D., Bharat, C., Degenhardt, L., Sampson, N.A., Scott, K.M., Lim, C.C.W., Al-Hamzawi, A., Alonso, J., Andrade L.H., Cardoso, G., De Girolamo, G., Gureje, O., He, Y., Hinkov, H., Karam, E.G., Karam. G., Kovess-Masfety, V., Lasebikan, V., Lee, S., Levinson, D., McGrath, J., Medina-Mora, M.E., Mihaescu-Pintia, C., Mneimneh, Z., Moskalewicz, Z., Navarro-Mateu. F., Posada-Villa, J., Rapsey, C., Stagnaro, J.C., Tachimori, H., Ten Have, M., Tintle, N., Torres, Y., Williams, D.R., Ziv, Y., Kessler, R.C., on behalf of the WHO World Mental Health Survey Collaborators. (2020). The epidemiology of alcohol use disorders cross-nationally: Findings from the World Mental Health Surveys.*Addictive Behaviors*, 102: 106128.
25. Oliveira, J., Paixão, V,, Cardoso, G., Xavier, M., Caldas de Almeida, J.M., Oliveira-Maia, A.J. (2021). Childhood adversities and the comorbidity between mood and general medical disorders in adults: Results from the WHO World Mental Health Survey Portugal. *Brain, Behavior & Immunity - Health*, 17: 100329.
26. Roest, A.M., de Vries, Y.A., Al-Hamzawi, A., Alonso, J., Ayinde, O., Bruffaerts, R., Bunting, B., Caldas-de-Almeida, J.M., de Girolamo, G., Degenhardt, L., Florescu, S., Gureje, O., Haro, J.M., Hu, C.Y., Karam, E.G., Kiejna, A., Kovess-Masfety, V., Lee, S., McGrath, J.J., Medina-Mora, M.E., Navarro-Mateu, F., Nishi, D., Piazza, M., Posada-Villa, J., Scott, K.M., Stagnaro, J.C., Stein, D.J., Torres, Y., Viana, M.C., Zarkov, Z., Kessler, R.C., de Jonge, P., on behalf of the WHO World Mental Health Survey collaborators. (2021). Previous disorders and depression outcomes in individuals with 12-month major depressive disorder in the World Mental Health surveys. *Epidemiology and Psychiatric Sciences*, 30: e70.
27. Scott, K.M., Zhang, Y., Chardoul, S., Ghimire, D.J., Smoller, J.W., Axinn, W.G. (2021). Resilience to mental disorders in a low-income, non-Westernized setting. *Psychological Medicine*, 51(16): 2825-2834
28. Vigo, D., Jones, L., Munthali, R., Pei, J., Westenberg, J., Munro, L., Judkowicz, C., Wang, A.Y., Van den Adel, B., Dulai, J., Krausz, M., Auerbach, R.P., Bruffaerts, R., Yatham, L., Gadermann, A., Rush, B., Xie, H., Pendakur, K., Richardson, C. (2021). Investigating the effect of COVID-19 dissemination on symptoms of anxiety and depression among university students. *BJPsych Open*, 7(2):e69
29. Voorspoels, W., Jansen, L., Mortier, P., Vilagut, G., De Vocht, J., Kessler, R.C., Alonso, J., Bruffaerts, R. (2021). Positive screens for mental disorders among healthcare professionals during the first Covid-19 wave in Belgium. *Journal of Psychiatric Research*, 140:329-336.
30. Nierenberg, A.A., Harris, M.G., Kazdin, A.E., Puac-Polanco, V., Sampson, N.A., Vigo, D.V., Chiu, W.T., Ziobrowski, H.N., Alonso, J., Altwaijri, Y., Borges, G., Bunting, B., Caldas-de-Almeida, J.M., Haro, J.M., Hu, C.Y., Kiejna, A., Lee, S., McGrath, J.J., Navarro-Mateu, F., Posada-Villa, J., Scott, K.M., Stagnaro, J.C., Viana, M.C., Kessler, R.C., and on behalf of the WHO World Mental Health Survey Collaborators. (2021). Perceived helpfulness of bipolar disorder treatment: Findings from the World Health Organization World Mental Health Surveys. *Bipolar Disorders*, 23(6):565-583.
31. Cuijpers, P., Smit, F., Aalten, P., Batelaan, N., Klein, A., Salemink, E., Spinhoven, P., Struijs, S., Vonk, P., Wiers, R.W., de Wit, L., Gentili, C., Ebert, D.D., Bruffaerts, R., Kessler, R.C., Karyotaki, E. (2021). The associations of common psychological problems with mental disorders among college students. *Frontiers in Psychiatry*, 12: 573637. [P](https://pubmed.ncbi.nlm.nih.gov/34646167/)
32. Cuijpers, P., Miguel, C., Ciharova, M., Aalten, P., Batelaan, N., Salemink, E., Spinhoven, P., Struijs, S., de Wit, L., Gentili, C., Ebert, D., Harrer, M., Bruffaerts, R., Kessler, R.C., Karyotaki, E. (2021). Prevention and treatment of mental health and psychosocial problems in college students: An umbrella review of meta-analyses. *Clinical Psychology: Science and Practice*, 28(3), 229-244.
33. Degenhardt, L., Bharat, C., Chiu, W.T., Harris, M., Kazdin, A.E., Vigo, D.V., Sampson, N., Alonso, J., Andrade, L.H., Bruffaerts, R., Bunting, B., Cardoso, G., de Girolamo, G., Florescu, S., Gureje, O., Haro, J.M., Hu, C.Y., Karam, A., Karam, E.G., Kovess-Masfety, V., Lee, S., Makanjuola, V., McGrath, J.J., Medina-Mora, M.E., Moskalewicz, J., Navarro-Mateu, F., Posada-Villa, J., Rapsey, C., Stagnaro, J.C., Tachimori, H., ten Have, M., Torres, Y., Williams, D.R., Zarkov, Z., Kessler, R.C., and on behalf of the WHO World Mental Health Survey collaborators. (2021). Perceived helpfulness of treatment for alcohol use disorders: Findings from the World Mental Health Surveys. *Drug and Alcohol Dependence*, 229 (Pt B): 109158.
34. Fernández, D., Vigo, D., Sampson, N.A., Hwang, I., Aguilar-Gaxiola, S., Al-Hamzawi, A., Alonso, J., Andrade, L.H., Bromet, E.J., de Girolamo, G., de Jonge, P., Florescu, S., Gureje, O., Hinkov, H. , Hu, C., Karam, E.G., Karam, G., Kawakami, N., Kiejna, A., Kovess-Masfety, A., Medina-Mora, M.E., Navarro-Mateu, F., Ojagbemi, A., O'Neill, S., Piazza, M., Posada-Villa, J., Rapsey, C., Williams, D.R., Xavier, M., Ziv. Y., Kessler, R.C., Haro, J.M., and on behalf of the World Health Organization World Mental Health Survey collaborators. (2021). Patterns of care and dropout rates from outpatient mental healthcare in low-, middle- and high-income countries from the World Health Organization's World Mental Health Survey Initiative. *Psychological Medicine*, 51(12): 2104-2116.
35. Ward, C., McLafferty, M., McLaughlin, J., McHugh, R., McBride, L., Brady, J., Bjourson, A.J., Walsh, C.P., O'Neill, S.M., Murray, E.K. (2022). Suicidal behaviours and mental health disorders among students commencing college. *Psychiatry Research*, 307: 114314.
36. Alonso, J., Vilagut, G., Mortier, P., Ferrer, M., Alayo, I., Aragón-Peña, A., Aragonès, E., Campos, M., del Cura-González, I., Emparanxa, J.I., Espuga, M., Forjaz, M.J., González-Pinto, A., Haro, J.M., Lopez-Fresneña, N., de Salázar, A.M., Molina, J.D., Orti-Lucas, R.M., Parellada, M., Pelayo-Terán, J.M., Pérez-Zapata, A.P., Pijoan, J.I., Plana, N., Puig, T., Ruis, C., Rodríguez-Blázquez, C., Sanz, F., Serra, C., Kessler, R.C., Bruffaerts, R., Vieta, E., Pérez-Solà, V., the MINDCOVID Working group. (2021). Mental health impact of the first wave of COVID-19 pandemic on Spanish healthcare Workers: a large cross-sectional survey. *Revista de Psiquiatría y Salud Mental*, 14(2):90-105.
37. Andersson, C., Bendtsen, M., Lindfors, P., Molander, O., Lindner, P., Topooco, N., Egström, K., Berman, A.H. (2021). Does the management of personal integrity information lead to differing participation rates and response patterns in mental health surveys with young adults? A three-armed methodological experiment. *International Journal of Methods in Psychiatric Research*, 30(4): e1891.
38. Axinn, W.G., Chardoul, S. (2021). Improving Reports of Health Risks: Life History Calendars and Measurement of Potentially Traumatic Experiences. *International Journal of Methods in Psychiatric Research*, 30(1): e1853.
39. Bantjes, J., Breet, E., Lochner, C., Roos, J., Kessler, R.C., Stein, D.J. (2021). Reducing nonfatal suicidal behaviour among university students: actuarial analysis of potential effects of treating common mental disorders. *South African Journal of Psychology*, 51(1): 21-34.
40. Coêlho BM, Andrade LH, Santana GL, Viana MC, Wang YP. (2021). Association between childhood adversities and psychopathology onset throughout the lifespan: Findings from a large metropolitan population. *Journal of Psychiatric Research*, 135:8-1
41. Sousa, R. D., Gouveia, M., Nunes da Silva, C., Rodrigues, A. M., Cardoso, G., Antunes, A. F., Canhao, H. & Caldas-de Almeida, J.M. (2022). Treatment-resistant depression and major depression with suicide risk-The cost of illness and burden of disease. *Frontiers in Public Health*, 10, 898491.
42. Vigo, D.V., Kazdin, A.E., Sampson, N.A., Hwang, I., Alonso, J., Andrade, L.H., Ayinde, O., Borges, G., Bruffaerts, R., Bunting, B., de Girolamo, G., Florescu, S., Gureje, O., Haro, J.M., Harris, M.G., Karam, E.G., Karam, G., Kovess-Masfety, V., Lee, S., Navarro-Mateu, F., Posada-Villa, J., Scott, K., Stagnaro, J.C., ten Have, M., Wu, C.S., Xavier, M., Kessler, R.C. (2022). Determinants of effective treatment coverage for major depressive disorder in the WHO World Mental Health Surveys. *International Journal of Mental Health Systems*, 16(1):29
43. Mak, A.D.P., Lee, S., Sampson, N.A., Albor, Y., Alonso, J., Auerbach, R.P., Baumeister, H., Benjet, C., Bruffaerts, R., Cuijpers, P., Ebert, D.D., Gutierrez-Garcia, R., Hasking, P., Lapsley, C., Lochner, C., Kessler, R.C., and on behalf of the WHO World Mental Health Survey International College Student collaborators. (2022). ADHD Comorbidity Structure and Impairment: Results of the WHO World Mental Health Surveys International College Student Project (WMH-ICS). *Journal of Attention Disorders*, 26(8):1078-1096.
44. McLafferty, M., Brown, N., Brady, J., McLaughlin, J., McHugh, R., Ward, C., McBride, L., Bjourson, A. J., O'Neill, S.M., Walsh, C.P., & Murray, E.K. (2022). Variations in psychological disorders, suicidality, and help-seeking behaviour among college students from different academic disciplines. *PloS One*, 17(12), e0279618.
45. Mortier, P., Alonso, J., Auerbach, R.P., Bantjes, J., Benjet, C., Bruffaerts, R., Cuijpers, P., Ebert, D.D., Green, J.G., Hasking, P., Karyotaki, E., Kiekens, G., Mak, A., Nock, M.K., O'Neill, S., Pinder-Amaker, S., Sampson, N.A., Stein, D.J., Vilagut, G., Wilks, C., Zaslavsky, A.M., Mair, P., Kessler, R.C., WHO WMH-ICS collaborators. (2022). Childhood adversities and suicidal thoughts and behaviors among first-year college students: results from the WMH-ICS initiative. *Social Psychiatry and Psychiatric Epidemiology*, 57(8):1591-1601.
46. Mortier, P., Vilagut, G., Alayo, I., Ferrer, M., Amigo, F., Aragonès, E., Aragón-Peña, A., Asúnsolo del Barco, A., Campos, M., Espuga, M., González-Pinto, A., Haro, J.M., López Fresneña, N., Martínez de Salázar, A., Molina, J.D., Ortí-Lucas, R.M., Parellada, M., Pelayo-Terán, J.M., Pérez-Gómez, B., Pérez-Zapata, A., Pijoan, J.I., Plana, N., Polentinos-Castro, E., Portillo-Van Dienst, A., Puig, M.T., Rius, C., Sanz, F., Serra, C., Urreta-Barallobre, I., Kessler, R.C., Bruffaerts, R., Vieta, E., Pérez- Solá, V., Alonso, J., on behalf of the MINDCOVID Working group. (2022) Four-month incidence of suicidal thoughts and behaviors among healthcare workers after the first wave of the Spain COVID-19 pandemic. *Journal of Psychiatric Research*, 149, 10-17.
47. Sanza, M., Monzio Compagnoni, M., Caggiu, G., Allevi, L., Barbato, A., Campa, J., Carle, F., D’avanzo, B., di Fiandra, T., Ferrara, L., Gaddini, A., Saponaro, A., Scondotto, S., Tozzi, V. D., Lorusso, S., Giordani, C., Corrao, G., & Lora, A. (2023). Assessing the quality of the care offer for people with personality disorders in Italy: the QUADIM project. A multicentre research based on the database of use of Mental Health services. *International Journal of Mental Health Systems*, *17*(1). https://doi.org/10.1186/s13033-023-00603-9
48. Wang, R. A. H., Smittenaar, P., Thomas, T., Kamal, Z., Kemp, H., & Sgaier, S. K. (2024). Geographical variation in perceptions, attitudes and barriers to mental health care-seeking across the UK: A cross-sectional study. *BMJ Open*, *14*(3). https://doi.org/10.1136/bmjopen-2023-073731
49. Doll, C. M., Michel, C., Rosen, M., Osman, N., Schimmelmann, B. G., & Schultze-Lutter, F. (2021). Predictors of help-seeking behaviour in people with mental health problems: a 3-year prospective community study. *BMC Psychiatry*, *21*(1). https://doi.org/10.1186/s12888-021-03435-4
50. Chaudhary, P., Fadnes, L. T., Fosse, S., Chalabianloo, F., & Johansson, K. A. (2024). Universal Health Coverage of Opioid Agonist Treatment in Norway: An Equity-Adjusted Economic Evaluation. *PharmacoEconomics*. https://doi.org/10.1007/s40273-024-01442-3
51. Karyotaki, E., Klein, A.M., Ciharova, M., Bolinski, F., Krijnen, L., de Koning, L., de Wit, L., van der Heijde, C.M., Ebert, D.D., Riper, H., Batelaan, N., Vonk, P., Auerbach, R.P., Kessler, R.C., Bruffaerts, R., Struijs, S., Wiers, R. W., & Cuijpers, P. (2022). Guided internet-based transdiagnostic individually tailored Cognitive Behavioral Therapy for symptoms of depression and/or anxiety in college students: A randomized controlled trial. *Behaviour Research and Therapy*, 150, 104028
52. Kessler, R.C., Kazdin, A.E., Aguilar-Gaxiola, S., Al-Hamzawi, A., Alonso, J., Altwaijri, Y., Andrade, L.H., Benjet, C., Bharat, C., Borges, G., Bruffaerts, R., Bunting, B., Caldas-de-Almeida, J.M., Cardoso, G., Chiu, W.T., Cia, A., Ciutan, M., Degenhardt, L., de Girolamo, G., de Jonge, P., de Vries, Y.A., Florescu, S., Gureje, O., Haro, J.M., Harris, M.G., Hu, C.Y., Karam, A.N., Karam, E.G., Karam, G., Kawakami, N., Kiejna, A., Kovess-Masfety, V., Lee, S., Makanjuola, V., McGrath, J.J., Medina-Mora, M.E., Moskalewicz, J., Navarro-Mateu, F., Nierenberg, A.N., Nishi, D., Ojagbemi, A., Oladeji, B.D., O'Neill, S., Posada-Villa, J., Puac-Polanco, V., Rapsey, C., Ruscio, A.M., Sampson, N.A., Scott, K.M., Slade, T., Stagnaro, J.C., Stein, D.J., Tachimori, H., ten Have, M., Torres, Y., Viana, M.C., Vigo, D.V., Williams, D.R., Wojtyniak, B., Xavier, M., Zarkov, Z., Ziobrowski, H.N., and on behalf of the WHO World Mental Health Survey collaborators. (2022). Patterns and correlates of patient-reported helpfulness of treatment for common mental and substance use disorders in the WHO World Mental Health Surveys. *World Psychiatry*, 21(2):272-286.
53. Leung, C., Pei, J., Hudec, K., Shams, F., Munthali, R., Vigo, D. (2022). The Effects of Nonclinician Guidance on Effectiveness and Process Outcomes in Digital Mental Health Interventions: Systematic Review and Meta-analysis. *Journal of Medical Internet Research*, 24(6):e36004.
54. Mak, A.D.P., Lee, S., Sampson, N.A., Albor, Y., Alonso, J., Auerbach, R.P., Baumeister, H., Benjet, C., Bruffaerts, R., Cuijpers, P., Ebert, D.D., Gutierrez-Garcia, R., Hasking, P., Lapsley, C., Lochner, C., Kessler, R.C., and on behalf of the WHO World Mental Health Survey International College Student collaborators. (2022). ADHD Comorbidity Structure and Impairment: Results of the WHO World Mental Health Surveys International College Student Project (WMH-ICS). *Journal of Attention Disorders*, 26(8):1078-1096
55. McLafferty, M., Brown, N., Brady, J., McLaughlin, J., McHugh, R., Ward, C., McBride, L., Bjourson, A. J., O'Neill, S.M., Walsh, C.P., & Murray, E.K. (2022). Variations in psychological disorders, suicidality, and help-seeking behaviour among college students from different academic disciplines. *PloS One*, 17(12), e0279618
56. Mortier, P., Alonso, J., Auerbach, R.P., Bantjes, J., Benjet, C., Bruffaerts, R., Cuijpers, P., Ebert, D.D., Green, J.G., Hasking, P., Karyotaki, E., Kiekens, G., Mak, A., Nock, M.K., O'Neill, S., Pinder-Amaker, S., Sampson, N.A., Stein, D.J., Vilagut, G., Wilks, C., Zaslavsky, A.M., Mair, P., Kessler, R.C., WHO WMH-ICS collaborators. (2022). Childhood adversities and suicidal thoughts and behaviors among first-year college students: results from the WMH-ICS initiative. *Social Psychiatry and Psychiatric Epidemiology*, 57(8):1591-1601
57. Degenhardt, L., Bharat, C., Glantz, M., Bromet, E., Alonso, J., Bruffaerts, R., Bunting, B., de Girolamo, G., de Jonge, P., Florescu, S., Gureje, O., Haro, J.M., Harris, M.G., Hinkov, H., Karam, E.G., Karam, G., Kovess-Masfety, V., Lee, S., Makanjuola, V., Medina-Mora, M.E., Navarro-Mateu, F., Piazza, M., Posada-Villa, J., Scott, K.M., Stein, D.J., Tachimori, H., Tintle, N., Torres, Y., Viana, M.C., Kessler, R.C., and on behalf of the WHO World Mental Health Survey collaborators. (2022). The associations between traumatic experiences and subsequent onset of a substance use disorder: Findings from the World Health Organization World Mental Health surveys. *Drug and Alcohol Dependence*, 240:109574.
58. de Vries, Y.A., Al-Hamzawi, A., Alonso, J., Andrade, L.H., Benjet, C., Bruffaerts, R., Bunting, B., De Girolamo, G., Florescu, S., Gureje, O., Haro, J.M., Karam, A., Karam, E.G., Kawakami, N., Kovess-Masfety, V., Lee, S., Mneimneh, Z., Navarro-Mateu, F., Ojagbemi, A., Posada-Villa, J., Scott, K.M., Stagnaro, J.C., Torres, Y., Xavier, M., Zarkov, Z., Kessler, R.C., de Jonge, P.; WHO World Mental Health Survey collaborators. (2022). Transdiagnostic development of internalizing psychopathology throughout the life course up to age 45: a World Mental Health Surveys report. *Psychological Medicine*, 52(11), 2134-2143
59. Gmelin, J.H., de Vries, Y.A., Baams, L., Aguilar-Gaxiola, S., Alonso, J., Borges, G., Bunting, B., Cardoso, G., Florescu, S., Gureje, O., Karam, E.G., Kawakami, N., Lee, S., Mneimneh, Z., Navarro-Mateu, F., Posada-Villa, J., Rapsey, C., Slade, T., Stagnaro, J.C., Torres, Y., Kessler, R.C., de Jonge, P., WHO World Mental Health Survey collaborators. (2022). Increased risks for mental disorders among LGB individuals - Cross-National evidence from the World Mental Health surveys. *Social Psychiatry and Psychiatric Epidemiology*, 57(11):2319-2332.
60. Ballester, L., Alayo, I., Vilagut, G., Mortier, P., Almenara, J., Cebrià, A.I., Echeburúa, E., Gabilondo, A., Gili, M., Lagares, C., Piqueras, J.A., Roca, M., Soto-Sanz, V., Blasco, M.J., Castellví, P., Miranda-Mendizábal, A., Bruffaerts, R., Auerbach, R.P., Nock, M.K., Kessler, R.C., Alonso, J., on behalf of the UNIVERSAL study group (2022). Predictive models for first-onset and persistence of depression and anxiety among university students. *Journal of Affective Disorders*, 308:432-441.
61. Benjet, C., Borges, G., Miah, S., Albor, Y., Gutiérrez-García, R. A., Zavala Berbena, A., Guzmán, R., Vargas-Contreras, E., Hermosillo de la Torre, A. E., Hernández Uribe, P. C., Quevedo, G., Covarrubias Díaz, A., Martínez Ruiz, S., Valdés-García, K. P., Martínez Jerez, A. M., & Mortier, P. (2022). One-year incidence, predictors, and accuracy of prediction of suicidal thoughts and behaviors from the first to second year of university. *Depression and Anxiety*, 39(12):727-740.
62. Benjet, C.J., Mortier, P., Kiekens, G., Ebert, D.D., Auerbach, R.P., Kessler, R.C., Cuijpers, P., Green, J.G., Nock, M.K., Demyttenaere, K., Albor, Y., Bruffaerts, R. (2022). A risk algorithm that predicts alcohol use disorders among college students. *European Child & Adolescent Psychiatry*, 31(7):1-11.
63. Berman, A.H., Bendtsen, M., Molander, O., Lindfors, P., Lindner, P., Granlund, L., Topooco, N., Engstrom, K., Andersson, C. (2022). Compliance with recommendations limiting COVID-19 contagion among university students in Sweden: associations with self-reported symptoms, mental health and academic self-efficacy. *Scandinavian Journal of Public Health*,50(1):70-84.
64. Preece, D.A., Kiekens, G., Boyes, M., Mortier, P., Nock, M.K., Kessler, R.C., Bruffaerts, R., Hasking, P. (2021). Acquired Capability for Suicide Among Belgian and Australian University Students: Psychometric Properties of the German Capability for Suicide Questionnaire and a test of the Interpersonal Theory of Suicide. *Suicide and Life-Threatening Behavior*, 51(3):403-415.
65. Karyotaki, E., Araya, R., Kessler, R.C., Waqas, A., Bhana, A., Rahman, A., Matsuzaka, C.T., Miguel, C., Lund, C., Garman, E.C., Nakimuli-Mpungu, E., Petersen, I., Naslund, J.A., Schneider, M., Sikander, S., Jordans, M.J.D., Abas, M., Slade, P., Walters, S., Brugha, T.S., Furukawa, T.A., Amanvermez, Y., Mello, M.F., Wainberg, M.L., Cuijpers, P., Patel, V. (2022). Association of Task-Shared Psychological Interventions with Depression Outcomes in Low- and Middle-Income Countries: A Systematic Review and Individual Patient Data Meta-analysis. *JAMA Psychiatry*, 79(5):430-443.
66. Kiekens, G., Claes, L., Hasking, P., Mortier, P., Bootsma, E., Boyes, M., Myin-Germeys, I., Demyttenaere, K., Cuijpers, P., Kessler, R.C., Nock, M.K., Bruffaerts, R. (2023). A longitudinal investigation of non-suicidal self-injury persistence patterns, risk factors, and clinical outcomes during the college period. *Psychological Medicine*, 53(13):6011-6026.
67. Borges, G., Benjet, C., Orozco, R., Albor, Y., Contreras, E.V, Monroy-Velasco, I.R., Hernández-Uribe, P.C., Báez-Mansur, P.M., Covarrubias Díaz Couder, M.A., Quevedo-Chávez, G.E., Gutierrez-García, R.A., Machado, N. (2023). Internet Gaming Disorder Does Not Predict Mood, Anxiety or Substance Use Disorders in University Students: A One-Year Follow-Up Study. *International Journal of Environmental Research and Public Health*, 20(3):2063. [PubMed Abstract](https://pubmed.ncbi.nlm.nih.gov/36767430/)
68. Ruscio, A.M, Rassaby, M., Stein, M.B., Stein, D.J., Aguilar-Gaxiola, S., Al-Hamzawi, A., Alonso, J., Atwoli, L., Borges, G., Bromet, E.B., Bruffaerts, R., Bunting, B., Cardoso, G., Chardoul, S., de Girolamo, G., de Jonge, P., Gureje, O., Haro, J.M., Karam, E.G., Karam, A., Kiejna, A., Kovess-Masfety, V., Lee, S., Navarro-Mateu, F., Nishi, D., Piazza, M., Posada-Villa, J., Sampson, N.A., Scott, K.M., Slade, T., Stagnaro, J.C., Torres, Y., Viana, M.C., Vladescu, C., Zarkov, Z., Kessler, R.C., and the World Mental Health Survey collaborators. (In press). The case for eliminating excessive worry as a requirement for generalized anxiety disorder: A cross-national investigation. *Psychological Medicine*
69. Kazdin, A. E., Harris, M. G., Hwang, I., Sampson, N. A., Stein, D. J., Viana, C. M., Vigo, D. V., Wu, C.-S., Aguilar-Gaxiola, S., Alonso, J., Benjet, C., Bruffaerts, R., Caldas-Almeida, J. M., Cardoso, G., Caselani, E., Chardoul, S., Cia, A., de Jonge, P., Gureje, O., Haro, J. M., Karam, E. G., Kovess-Masfety, V., Navarro-Mateu, F., Piazza, M., Posada-Villa, J., Scott, K. M., Stagnaro, J. C., ten Have, M., Torres, Y., Vladescu, C., & Kessler, R. C. (2024). Patterns, predictors, and patient-reported reasons for antidepressant discontinuation in the WHO World Mental Health Surveys. *Psychological Medicine*, 54(1), 67-78.
70. Husky, M. M., Pic, O., Callahan, S., & Navarro-Mateu, F. (2024). Twelve-month suicidal ideation, incidence and persistence among college students pre-pandemic and during the pandemic: A longitudinal study. *Psychiatry Research*, 331, 115669
71. Navarro-Mateu, F., Salmerón, D., Vilagut, G., Husky, M., Ballesta, M., Chirlaque, M.D., Huerta, J.M., Martínez, S., Navarro, C., Alonso, J., Nock, M.K., Kessler, R.C. (2024). Childhood adversities and suicidal behavior in the general population. The cross-sectional PEGASUS-Murcia Project. *Spanish Journal of Psychiatry and Mental Health*, 17(1), 11-18.
72. Pei, J., Amanvermez, Y., Vigo, D., Puyat, J., Kessler, R.C., Mortier, P., Bruffaerts, R., Rankin, O., Chua, S.N., Martínez, V., Rapsey, C., Fodor, L.A., David, O.A., Garcia, C. & Cuijpers, P. (2024). Sociodemographic Correlates of Mental Health Treatment Seeking Among College Students: A Systematic Review and Meta-Analysis. *Psychiatric Services*, 75(6), 556-569.
73. Roldán-Espínola, L., Riera-Serra, P., Roca, M., García-Toro, M., Coronado-Simsic, V., Castro, A., Navarra-Ventura, G., Vilagut, G., Alayo, I., Ballester, L., Blasco, M.J., Almenara, J., Cebrià, A.I., Echeburúa, E., Gabilondo, A., Lagares, C., Piqueras, J.A., Soto-Sanz, V., Mortier, P., Kessler, R.C., Alonso, J., Forteza-Rey, I., Gili,M. (2024). Depression and lifestyle among university students: A one-year follow-up study. *The European Journal of Psychiatry*, 38(3): 100250.
74. Axinn, W.G., Bruffaerts, R., Kessler, T.L., Frounfelker, R., Aguilar-Gaxiola, S., Alonso, J., Bunting, B., Caldas-de-Almeida J.M., Cardoso, G., Chardoul, S., Chiu, W.T., Cía, A., Gureje, O., Karam, E.G., Kovess-Masfety, V., Petukhova, M.V., Piazza, M., Posada-Villa, J., Sampson, N.A., Scott, K.M., Stagnaro, J.C., Stein, D.J., Torres, Y., Williams, D.R., Kessler, R.C. & WHO World Mental Health Survey Collaborators. (2023). The association of exposure to civil violence with the subsequent onset and persistence of mental disorders: Results from the World Mental Health Surveys. *JAMA Network Open* 6(6), e2318919.
75. Biscond, M., Revranche, M., Navarro-Mateu, F., Janota, M., Kovess-Masfety, V., & Husky, M. M. (2023). The effect of childhood adversities on the persistence of suicidal ideation and plans among college students: A longitudinal study. *Journal of Affective Disorders*, 323, 354-360
76. McGrath, J. J., Al-Hamzawi, A., Alonso, J., Altwaijri, Y., Andrade, L. H., Bromet, E. J., Bruffaerts, R., Caldas de Almeida, J. M., Chardoul, S., Chiu, W. T., Degenhardt, L., Demler, O. V., Ferry, F., Gureje, O., Haro, J. M., Karam, E. G., Karam, G., Khaled, S. M., Kovess-Masfety, V., Magno, M., Medina-Mora, M. E., Moskalewicz, J., Navarro-Mateu, F., Nishi, D., Plana-Ripoll, O., Posada-Villa, J., Rapsey, C., Sampson, N. A., Stagnaro, J. C., Stein, D. J., ten Have, M., Torres, Y., Vladescu, C., Woodruff, P. W., Zarkov, Z., Kessler, R. C., WHO World Mental Health Survey collaborators. (2023). Age-of-onset and cumulative risk of mental disorders: A cross-national analysis of population surveys data based on 156,331 respondents from 29 countries. *Lancet Psychiatry*, 10(9):668-681.
77. Portillo-Van Diest, A., Vilagut, G., Alayo, I., Ferrer, M., Amigo, F., Amann, B.L., Aragón-Peña, A., Aragonès, E., Asúnsolo Del Barco, Á., Campos, M., Del Cura-González, I., Espuga, M., González-Pinto, A., Haro, J.M., Larrauri, A., López-Fresneña, N., Martínez de Salázar, A., Molina, J.D., Ortí-Lucas, R.M., Parellada, M., Pelayo-Terán, J.M., Pérez-Zapata, A., Pijoan, J.I., Plana, N., Puig, T., Rius, C., Rodríguez-Blázquez, C., Sanz, F., Serra, C., Urreta-Barallobre, I., Kessler, R.C., Bruffaerts, R., Vieta, E., Pérez-Solá, V., Alonso, J., Mortier, P. & MINDCOVID Working Group. (2023). Traumatic stress symptoms among Spanish healthcare workers during the COVID-19 pandemic: a prospective study. *Epidemiology & Psychiatric Sciences*, 32, e50.
78. Sivertsen, B., Knudsen, A.K., Kirkøen, B., Skogen, J.C., Lagerstrøm, B.O., Lønning, K-J., Kessler, R.C., Reneflot. A. (2023). Prevalence of mental disorders among Norwegian college and university students: a population-based cross-sectional analysis. *The Lancet Regional Health - Europe*, 19(34), 100732.
79. Alonso, J., Vilagut, G., Alayo, I., Ferrer, M., Amigo, F., Aragón-Peña, A., Aragonès, E., Campos, M., Del Cura-González, I., Urreta, I., Espuga, M., González Pinto, A., Haro, J.M., López Fresneña, N., Martínez de Salázar, A., Molina, J.D., Ortí Lucas, R.M., Parellada, M., Pelayo-Terán, J.M., Pérez Zapata, A., Pijoan, J.I., Plana, N., Puig, M.T., Rius, C., Rodriguez-Blazquez, C., Sanz, F., Serra, C., Kessler, R.C., Bruffaerts, R., Vieta, E., Pérez-Solá, V., Mortier, P., and MINDCOVID Working group. (2022). Mental impact of Covid-19 among Spanish healthcare workers. A large longitudinal survey. *Epidemiology and Psychiatric Sciences*, 31: e28.
80. Aluh, D. O., Azeredo-Lopes, S., Cardoso, G., Pedrosa, B., Grigaitè, U., Dias, M., Xavier, M., & Caldas-de-Almeida, J. M. (2022). Social anxiety disorder and childhood adversities in Portugal: Findings from the WHO world mental health survey initiative. *Psychiatry Research*, 315, 114734.
81. Amanvermez, Y., Zhao, R., Cuijpers, P., de Wit, L.M., Ebert, D.D., Kessler, R.C., Bruffaerts, R., & Karyotaki, E. (2022). Effects of self-guided stress management interventions in college students: A systematic review and meta-analysis. *Internet Interventions*, 28, 100503.
82. Antunes, A., Silva, M., Azeredo-Lopes, S., Cardoso, G., Caldas-de-Almeida, J.M. (2022). Perceived stigma and discrimination among persons with mood and anxiety disorders: Results from the WHO World Mental Health Survey Portugal. *The European Journal of Psychiatry*, 36(4): 280-287.
83. Harrer, M., Baumeister, H., Cuijpers, P., Heber, E., Lehr, D., Kessler, R. C., & Ebert, D. D. (2024). Predicting effects of a digital stress intervention for patients with depressive symptoms: Development and validation of meta-analytic prognostic models using individual participant data. *Journal of Consulting and Clinical Psychology*, 92(4):226-235
84. Mortier, P., Vilagut, G., Alayo, I., Ferrer, M., Amigo, F., Aragonès, E., Aragón-Peña, A., Asúnsolo del Barco, A., Campos, M., Espuga, M., González-Pinto, A., Haro, J.M., López Fresneña, N., Martínez de Salázar, A., Molina, J.D., Ortí-Lucas, R.M., Parellada, M., Pelayo-Terán, J.M., Pérez-Gómez, B., Pérez-Zapata, A., Pijoan, J.I., Plana, N., Polentinos-Castro, E., Portillo-Van Dienst, A., Puig, M.T., Rius, C., Sanz, F., Serra, C., Urreta-Barallobre, I., Kessler, R.C., Bruffaerts, R., Vieta, E., Pérez- Solá, V., Alonso, J., on behalf of the MINDCOVID Working group. (2022) Four-month incidence of suicidal thoughts and behaviors among healthcare workers after the first wave of the Spain COVID-19 pandemic. *Journal of Psychiatric Research*, 149, 10-17.
85. Kessler, R.C., Kazdin, A.E., Aguilar-Gaxiola, S., Al-Hamzawi, A., Alonso, J., Altwaijri, Y., Andrade, L.H., Benjet, C., Bharat, C., Borges, G., Bruffaerts, R., Bunting, B., Caldas-de-Almeida, J.M., Cardoso, G., Chiu, W.T., Cia, A., Ciutan, M., Degenhardt, L., de Girolamo, G., de Jonge, P., de Vries, Y.A., Florescu, S., Gureje, O., Haro, J.M., Harris, M.G., Hu, C.Y., Karam, A.N., Karam, E.G., Karam, G., Kawakami, N., Kiejna, A., Kovess-Masfety, V., Lee, S., Makanjuola, V., McGrath, J.J., Medina-Mora, M.E., Moskalewicz, J., Navarro-Mateu, F., Nierenberg, A.N., Nishi, D., Ojagbemi, A., Oladeji, B.D., O'Neill, S., Posada-Villa, J., Puac-Polanco, V., Rapsey, C., Ruscio, A.M., Sampson, N.A., Scott, K.M., Slade, T., Stagnaro, J.C., Stein, D.J., Tachimori, H., ten Have, M., Torres, Y., Viana, M.C., Vigo, D.V., Williams, D.R., Wojtyniak, B., Xavier, M., Zarkov, Z., Ziobrowski, H.N., and on behalf of the WHO World Mental Health Survey collaborators. (2022). Patterns and correlates of patient-reported helpfulness of treatment for common mental and substance use disorders in the WHO World Mental Health Surveys. *World Psychiatry*, 21(2):272-286.
86. Husky, M. M., Kovess-Masfety, V., Gobin-Bourdet, C., Swendsen, J. (2021). Prior depression predicts greater stress during covid-19 mandatory lockdown among college students in France. *Comprehensive Psychiatry*, 107, 152234.
87. Hyder, S., Bilal, L., Mneimneh, Z.,.& Altwaijri, Y. (2021). Content analysis and predicting survey refusal: Why do respondents refuse to participate in a household mental health survey? *Field Methods*, 33(2): 125-142
88. Knudsen, A.K., Stene-Larsen, K., Gustavson, K., Hotopf, M., Kessler, R.C., Krokstad, S., Skogen, J.C., Øverland, S., Reneflot, A. (2021). Prevalence of mental disorders, suicidal ideation and suicides in the general population before and during the COVID-19 pandemic in Norway. A population-based repeated cross-sectional analysis from the HUNT study and the Norwegian Cause of Death Registry. *The Lancet Regional Health-Europe*, 4, 100071. [F](https://www.sciencedirect.com/science/article/pii/S266677622100048X/)
89. Lokkerbol, J., Wijnen, B.F.M., Chatterji, S., Kessler, R.C., Chisholm, D. (2021). Mapping of the World Health Organization's Disability Assessment Schedule 2.0 to Disability Weights using the Multi-Country Survey Study on Health and Responsiveness. *International Journal of Methods in Psychiatric Research*, 30(3):e1886.
90. McLafferty, M., Brown, N., McHugh, R., Ward, C., Stevenson, A., McBride, L., Brady, J., Bjourson, A.J., ONeill, S.M., Walsh, C.P., Murray, E.K. (2021). Depression, Anxiety and Suicidal Behaviour Among College Students: Comparisons Pre-Covid-19 and During the Pandemic. *Psychiatry Research Communications*, 1(2): 100012.
91. Mortier, P., Vilagut, G., Ferrer, M., Alayo, I., Bruffaerts, R., Cristóbal-Narváez, P., del Cura-González, I., Domènech-Abella, J., Felez-Nobrega, M., Olaya, B., Pijoan, J.I., Vieta, E., Pérez-Solà, V., Kessler, R.C., Haro. J.M., Alonso, J. (2021). Thirty-day suicidal thoughts and behaviours in the Spanish adult general population during the first wave of the Spain COVID-19 pandemic. *Epidemiology and Psychiatric Sciences*, 30, E19.
92. Mortier, P., Vilagut, G., Ferrer, M., Serra, C., de Dios Molina, J., Lopez-Fresneña, N., Puig, T., Pelayo-Terán, J.M., Pijoan, J.I., Emparanxa, J.I., Espuga, M., Plana, N., González-Pinto, A., Orti-Lucas, R.M., de Salázar, A.M., del Cura-González, I., Aragón-Peña, A., Campos, M., Parellada, M., Zapata, A.P., Forjaz, M.J., Sanz, F., Haro, J.M., Vieta, E., Pérez-Solà, V., Kessler, R.C., Bruffaerts, R., and Alonso, J., the MINDCOVID Working group. (2021). Thirty-Day Suicidal Thoughts and Behaviors among Hospital Workers during the First Wave of the Spain COVID-19 Outbreak. *Depression & Anxiety*, 38(5):528-544
93. Husky, M.M., Bharat, C., Vilagut, G., Salmerón, D., Martínez, S., Navarro, C., Alonso, J., Kessler, R.C., Navarro-Mateu, F. (2020). Birth-sex cohort use and alcohol use transitions in the general population: the cross-sectional PEGASUS-Murcia project. *Addiciones*, 32(2):94-104.
94. Husky, M. M., Kovess-Masfety, V., Swendsen, J. D. (2020). Stress and anxiety among university students in France during Covid-19 mandatory confinement. *Comprehensive Psychiatry*, 102: 152191.
95. Karyotaki, E., Cuijpers, P., Albor, Y., Alonso, J., Auerbach, R.P., Bantjes, J., Bruffaerts, R., Ebert, D.D., Hasking, P., Kiekens, G., Lee, S., McLafferty, M., Mak. A., Mortier, P., Sampson, N., Stein, D.J., Vilagut, G., Kessler, R.C. (2020). Sources of stress and their association with mental disorders in college students: Results of the WHO World Mental Health Surveys International College Student Initiative.*Frontiers in Psychology*, 11:1759.
96. Kawakami, N., Thi Thu Tran, T., Watanabe, K., Imamura, K., Thanh Nguyen, H., Sasaki, N., Kuribayashi, K., Sakuraya, A., Thuy Nguyen, Q., Thi Nguyen, N., Minh Bui, T., Thi Huong Nguyen, G., Minas, H., Tsutsumi, A. (2020). Internal consistency reliability, construct validity, and item response characteristics of the Kessler 6 scale among hospital nurses in Vietnam. *PLoS One*, 15(5):e0233119.
97. McGrath, J. J., Lim, C. C. W., Plana-Ripoll, O., Holtz, Y., Agerbo, E., Momen, N.C., Mortensen, P.B., Pedersen, C.B., Abdulmalik, J., Aguilar-Gaxiola, S., Al-Hamzawi, A., Alonso, J., Bromet, E.J., Bruffaerts, R., Bunting, B., Caldas de Almeida, J.M., de Girolamo, G., De Vries, Y.A., Florescu, S., Gureje, O., Haro, J.M., Harris, M.G., Hu, C.Y., Karam, E.G., Kawakami, N., Kiejna, A., Kovess-Masfety, V., Lee, S., Mneimneh, Z., Navarro-Mateu, F., Orozco, R., Posada-Villa, J., Roest, A.M., Saha, S., Scott, K.M., Stagnaro, J.C., Stein, D.J., Torres, Y., Viana, M.C., Ziv, Y., Kessler, R.C., de Jonge, P. (2020). Comorbidity within mental disorders: a comprehensive analysis based on 145,990 survey respondents from 27 countries. *Epidemiology and Psychiatric Sciences*, 29:e153.
98. McLafferty, M., Bunting, B.P, Armour, C., Lapsley, C., Ennis, E., Murray, E., O'Neill, S.M. (2020). The mediating role of emotion regulation strategies on psychopathology and suicidal behaviour following negative childhood experiences. *Children and Youth Services Review*, 116.
99. Ferriani, L.O., Coutinho, E.S.F., Silva, D.A., Bivanco-Lima, D., Benseñor, I.J.M., Viana, M.C. (2019). Validity of self-reported measures of body weight and height in participants of the São Paulo Megacity Mental Health Survey.[Article in Portuguese]. *Cadernos Saúde Coletiva*, 27(2).
100. Ferriani, L. O., Coutinho, E. S. F., Silva, D. A., Faria, C. P., Molina, M. D. C. B., Benseñor, I. J. M., & Viana, M. C. (2019). [Underestimation of obesity and overweight based on self-report measures in the general population: prevalence and a proposal for correction models].[Article in Portuguese]. *Cadernos de Saude Publica*, 35(6), e0006561
101. Harrer, M., Adam, S.H., Baumeister, H., Cuijpers, P., Karyotaki, E., Auerbach, R.P., Kessler, R.C., Bruffaerts, R., Berking, M., Ebert, D.D. (2019). Internet interventions for mental health in university students: A systematic review and meta-analysis. *International Journal*
102. .G., Koenen, K.C., Lee, S., Liu, H., Pennell, B-E., Petukhova, M.V., Sampson, N.A., Shahly, V.L., Stein, D.J., Atwoli, L., Borges, G., Bunting, B., de Girolamo, G., Gluzman, S., Haro, J.M., Hinkov, H., Kawakami, N., Kovess-Masfety, V., Navarro-Mateu, F., Posada-Villa, J., Scott, K.M., Shalev, A.Y., ten Have, M., Torres, Y., Viana, M.C., Zaslavsky, A.M., WHO World Mental Health Survey collaborators. (2018). The associations of earlier trauma exposures and history of mental disorders with PTSD after subsequent traumas. *Molecular Psychiatry*, 23(9):1892-1899
103. Kovess-Masfety, V., Saha, S., Lim, C.C.W., Aguilar-Gaxiola, S. , Al-Hamzawi, A., Alonso, J., Borges, G., de Girolamo, G., de Jonge, P., Demyttenaere, K., Florescu, S., Haro, J.M., Hu, C., Karam, E.G., Kawakami, N., Lee, S., Lepine, J.P. , Navarro-Mateu, F., Stagnaro, J.C. , ten Have, M. , Viana, M.C. , Kessler, R.C. , McGrath, J.J. , on behalf of the WHO World Mental Health Survey Collaborators. (2018). Psychotic experiences and religiosity: Data from the WHO World Mental Health Surveys. *Acta Psychiatrica Scandinavica*, 137(4):306-315.
104. McGrath, J.J., Saha, S., Lim, C.C.W., Gureje, O., Florescu, S. (2018). Psychotic Experiences. In K.M Scott, P. de Jonge, D.J. Stein, R.C Kessler (Eds.), Mental Disorders Around the World: Facts and Figures from the WHO World Mental Health Surveys,(pp. 286-296). New York: Cambridge University Press.
105. McLafferty, M., Armour, C., & O’Neill, S. (2018). Adverse Childhood Experiences (ACEs) among university students and the relationship with lifetime mood and anxiety disorders. *European Journal of Trauma and Dissociation, Stress Points*, 32(4).
106. McLafferty, M., O’Neill, S., Armour, C., Murphy, S., Ferry, F., & Bunting, B. (2018). The impact of childhood adversities on the development of Posttraumatic Stress Disorder (PTSD) in the Northern Ireland population. *European Journal of Trauma and Dissociation*, 3(2):135-141.
107. McLafferty, M., O’Neill, S., Armour, C., Murphy, S. & Bunting, B. (2018). The mediating role of various types of social networks on psychopathology following adverse childhood experiences. *Journal of Affective Disorders*, 238, 547-553.
108. McLafferty, M., O'Neill, S.M., Murphy, S., Armour, C., Ferry, F., & Bunting, B.P. (2018). The moderating impact of childhood adversity profiles and conflict on psychological health and suicidal behaviour in the Northern Ireland population. *Psychiatry Research*262: 213-220.
109. O'Neill, S., McLafferty, M., Ennis E., Lapsley, C., Bjourson, T., Armour, C., Murphy, S., Bunting, B., Murray E. (2018). Socio-demographic, mental health and childhood adversity risk factors for self-harm and suicidal behaviour in College students in Northern Ireland. *Journal of Affective Disorders*, 239:58-65
110. Ratanatharathorn, A., Ng, L.C., Navarro-Mateu, F., Koenen, K.C. (2018). Posttraumatic Stress Disorder. In K.M Scott, P. de Jonge, D.J. Stein, R.C Kessler (Eds.), Mental Disorders Around the World: Facts and Figures from the WHO World Mental Health Surveys (pp. 153-166). New York: Cambridge University Press
111. Scott, K.M., Koenen, K.C., King, A., Petukhova, M.V., Alonso, J., Bromet, E.J., Bruffaerts, R., Bunting, B., de Jonge, P., Haro, J.M., Karam, E.G., Lee, S., Medina-Mora, M.E., Navarro-Mateu, F., Sampson, N.A., Shahly, V., Stein, D.J., Torres, Y., Zaslavsky, A.M. Kessler, R.C. (2018). Post-traumatic stress disorder associated with sexual assault among females in the WHO World Mental Health Surveys. *Psychological Medicine*, 48(1):155-167.
112. Scott, K.M, Saha, S., Lim, C.C.W., Aguilar-Gaxiola, S., Al-Hamzawi, A., Alonso, J., Benjet, C., Bromet, E.J., Bruffaerts, R., Caldas-de-Almeida, J.M, de Girolamo, G., de Jonge, P., Degenhardt, L., Florescu, S., Gureje, O., Haro, J.M., Hu, C., Karam, E.G., Kovess-Masfety, V., Lee, S., Lepine, J.P., Mneimneh, Z., Navarro-Mateu, F., Piazza, M., Posada-Villa, J., Sampson, N., Stagnaro, J.C., Kessler, R.C., McGrath, J.J. (2018). Psychotic experiences and general medical conditions: a cross-national analysis based on 28,002 respondents from 16 countries in the WHO World Mental Health Surveys. *Psychological Medicine*, 48(16): 2730-2739.
113. Viana, M.C., Lim, C.C.W., Pereira, F.G., Aguilar-Gaxiola, S., Alonso, J., Bruffaerts. R., de Jonge, P., Caldas-de-Almeida, J.M., O’Neill, S.M, Stein, D.J., Al-Hamzawi, A., Benjet, C., Cardoso, G., Florescu, S., de Girolamo, G., Haro, J.M., Hu, C., Kovess-Masfety, V., Levinson, D., Nakane, Y., Piazza, M., Posada-Villa, J., Rabczenko, D., Kessler, R.C., Scott, K.(2018). Prior mental disorders and subsequent onset of chronic back or neck pain: findings from 19 countries. *The Journal of Pain*, 19(1): 99-110.
114. Atwoli, L., Stein, D.J., King, A., Petukhova, M., Aguilar-Gaxiola, S., Alonso, J., Bromet, E.J., de Girolamo, G., Demyttenaere, K., Florescu, S., Haro, J.M., Karam, E.G., Kawakami, N., Lee, S., Lepine, J.P., Navarro-Mateu, F., O'Neill, S., Pennell, B.E., Piazza, M., Posada-Villa, J., Sampson, N.A., ten Have, M., Zaslavsky, A.M, Kessler, R.C., the World Health Organization World Mental Health Survey Collaborators. (2017). Posttraumatic stress disorder associated with unexpected death of a loved one: Cross-national findings from the World Mental Health Surveys. *Depression and Anxiety*, 34(4), 315-326.
115. Bromet, E.J., Nock, M.K., Saha, S., Lim, C.W.W., Aguilar-Gaxiola, S., Al-Hamzawi, A., Alonso, J., Borges, G., Bruffaerts, R., Degenhardt, L., de Girolamo, G., de Jonge, P., Florescu, S., Gureje, O., Haro, J.M., He, Y., Hu, C., Karam, E.G., Kovess-Masfety, V., Lee, S., Lepine, J.P., Mneimneh, Z., Navarro-Mateu, F., Ojagbemi, A., Posada-Villa, J., Sampson, N., Scott, K.M., Stagnaro, J.C., Viana, M.C., Xavier, M., Kessler, R.C., McGrath, J.J., for the World Health Organization World Mental Health Survey Collaborators. (2017). Association between psychotic experiences and subsequent suicidal thoughts and behaviours: A cross-national analysis from the World Health Organization World Mental Health Surveys. *JAMA Psychiatry*, 74(11):1136-1144.
116. Bromet, E.J., Atwoli, L., Kawakami, N., Navarro-Mateu, F., Piotrowski, P, King, A.J., Aguilar-Gaxiola, S., Alonso, J., Bunting, B., Demyttenaere, K., Florescu, S., de Girolamo, G., Gluzman, S., Haro, J.M., de Jonge, P., Karam, E.G., Lee, S., Kovess-Masfety, V., Medina-Mora,M.E., Mneimneh, Z., Pennell, B.E., Posada-Villa, J., Salmerón, D., Takeshima, T., Kessler, R.C. (2017). Post-traumatic stress disorder associated with natural and human-made disasters in the World Mental Health Surveys. *Psychological Medicine*, 47(2), 227-241.
117. Caldas-de-Almeida, J.M., Silva, M., Frasquilho, D., Antunes, A., Cardoso, G., (2017) Implicações do estudo do impacto da crise económica na saúde mental dos Portugueses [Implications of the study of the impact of the economic crisis on the mental health of the Portuguese population]. *Psilogos*, 15(2).
118. Cardoso, G., Xavier, M., Vilagut, G., Petukhova, M., Alonso, J., Kessler, R.C., Caldas-de-Almeida, J.M. (2017). Days Out of Role due to Common Physical and Mental Conditions in Portugal: Results from the WHO World Mental Health Survey. *British Journal of Psychiatry Open,*3(1), 15-21.
119. Chiavegatto Filho, A., Sampson, L., Martins, S.S., Yu, S., Huang, Y., He, Y., Lee, S., Hu, C., Zaslavsky, A.M., Kessler, R.C., Galea, S. (2017). Neighborhood characteristics and mental disorders in three large Chinese cities: Results from the World Mental Health Surveys.*BMJ Open*, 7(10):e017679
120. Herrera, A.V., Benjet, C., Mendez, E., Casanova, L., Medina-Mora, M.E. (2017) How Mental Health Interviews Conducted Alone, in the Presence of an Adult, a Child or Both Affects Adolescents' Reporting of Psychological Symptoms and Risky Behaviors. *Journal of Youth and Adolescence,*46(2), 417-428
121. Kessler, R.C., van Loo, H.M., Wardenaar, K.J., Bossarte, R.M., Brenner, L.A., Ebert, D.D., de Jonge, P., Nierenberg, A.A., Rosellini, A.J., Sampson, N.A., Schoevers, R.A., Wilcox, M.A., Zaslavsky, A.M. (2017). Using patient self-reports to study heterogeneity of treatment effects in major depressive disorder. *Epidemiology and Psychiatric Sciences*, 26(1):22-36.
     Related Commentaries: Cuijpers, P., Christensen, H. (2017). Are personalised treatments of adult depression finally within reach? *Epidemiology and Psychiatric Sciences*, 26:40-42.
     Rush, A.J. (2017) Targeting treatments for depression: what can our patients tell us? *Epidemiology and Psychiatric Sciences*, 26, 37-39
122. Kessler, R.C., Aguilar-Gaxiola, S., Alonso, J., Benjet, C., Bromet, E.J., Cardoso, G., Degenhardt, L., de Girolamo, G., Dinolova, R.V., Ferry, F., Florescu, S., Gureje, O., Haro, J.M., Huang, Y., Karam, E.G., Kawakami, N., Lee, S., Lepine, J.P., Levinson, D., Navarro-Mateu, F., Pennell, B.E., Piazza, M., Posada-Villa, J., Scott, K.M., Stein, D.J., ten Have, M., Torres, Y., Viana, M.C., Petukhova, M.V., Sampson, N.A., Zaslavsky, A.M., Koenen, K.C. (2017). Trauma and PTSD in the WHO World Mental Health Surveys. *European Journal of Pyschotraumatology*, 8(sup5):1353383.
123. Koenen, K.C., Ratanatharathorn, A., Ng, L., McLaughlin, K., Bromet, E.J., Stein, D.J., Karam, E.G., Ruscio, A.M., Benjet, C., Scott, K., Atwoli, L., Petukhova, M., Lim, C.C.W., Aguilar-Gaxiola, S., Al-Hamzawi, A., Alonso, J., Bunting, B., Ciutan, M., de Girolamo, G., Degenhardt, L., Gureje, O., Haro, J.M., Huang, Y., Kawakami, N., Lee, S., Navarro-Mateu, F., Pennell, B. E., Piazza, M., Sampson, N., ten Have, M., Torres, Y., Viana, M.C., Williams, D., Xavier, M., Kessler, R.C. (2017). Posttraumatic Stress Disorder in the World Mental Health Surveys. *Psychological Medicine*, 47(13):2260-2274
124. Kovess-Masfety, V., Evans-Lacko, S., Williams, D., Andrade, L.H., Benjet, C., Ten Have, M., Wardenaar, K., Karam, E.G., Bruffaerts, R., Abdumalik, J., Haro Abad, J.M., Florescu, S., Wu, B., De Jonge, P., Altwaijri, Y., Hinkov, H., Kawakami, N., Caldas-de-Almeida, J.M., Bromet, E.J., de Girolamo, G., Posada-Villa, J., Al-Hamzawi, A., Huang, Y., Hu, C., Viana, M.C., Fayyad, J., Medina-Mora, M.E., Demyttenaere, K., Lepine, J.P., Murphy, S., Xavier, M., Takeshima, T., Gureje, O. (2017). The role of religious advisors in mental health care in the World Mental Health surveys. *Social Psychiatry and Psychiatric Epidemiology*, 52(3), 353-367.
125. Lago, L., Glantz, M., Kessler, R.C., Sampson, N., Al-Hamzawi, A., Florescu, S., Moskalewicz, J., Murphy, S., Navarro-Mateu, F., Torres de Galvis, Y., Viana, M.C., Xavier, M., Degenhardt, L. (2017) Substance Dependence among those without symptoms of Substance Abuse in the World Mental Health Survey. *International Journal of Methods in Psychiatric Research*, 26(3)
126. Liu, H., Petukhova, M.V., Sampson, N.A., Aguilar-Gaxiola, S., Alonso, .J, Andrade, L.H., Bromet, E.J, de Girolamo, G., Haro, J.M., Hinkov, H., Kawakami, N., Koenen, K.C., Kovess-Masfety, V., Lee, S., Medina-Mora, M.E, Navarro-Mateu, F., O'Neill, S., Piazza, M., Posada-Villa, J., Scott, K.M., Shahly, V., Stein, D.J., Ten Have, M., Torres, Y., Gureje, O., Zaslavsky, A.M., Kessler, R.C.; World Health Organization World Mental Health Survey Collaborators. (2017). Association of DSM-IV Posttraumatic Stress Disorder With Traumatic Experience Type and History in the World Health Organization World Mental Health Surveys. *JAMA Psychiatry,*74(3), 270-281.
127. McGrath, J.J., McLaughlin, K.A., Saha, S., Aguilar-Gaxiola, S., Al-Hamzawi, A., Alonso, J., Bruffaerts, R., de Girolamo, G., de Jonge, P., Esan, O., Florescu, S., Gureje, O., Haro, J.M., Hu, C.Y., Karam, E.G., Kovess-Masfety, V., Lee, S., Lepine, J., Lim, C.C.W., Medina-Mora, M.E., Mneimneh, Z., Pennell, B.E., Piazza, M., Posada-Villa, J., Sampson, N., Viana, M.C., Xavier, M., Bromet, E.J., Kendler, K.S., Kessler, R.C. (2017). The association between childhood adversities and subsequent first onset of psychotic experiences: a cross-national analysis of 23,998 respondents from 17 countries. *Psychological Medicine*, 47(7), 1230-1245.
128. McGrath, J., Saha, S., Lim, C.C.W., Aguilar-Gaxiola, S., Alonso, J., Andrade, L., Bromet, E.J., Bruffaerts, R., Caldas de Almeida, J.M., Cardoso, G., de Girolamo, G., Fayyad, J., Florescu, S., Gureje, O. Haro, J.M., Kawakami, N., Koenen, K., Kovess-Masfety, V., Lee, S., Lépine, J.P., McLaughlin, K., Medina-Mora, M.E., Navarro-Mateu, F., Ojagbemi, A., Posada-Villa, J., Sampson, N., Scott, K., Tachimori, H., ten Have, M., Kendler, K., Kessler, R.C., WHO World Mental Health Survey collaborators. (2017). Trauma and psychotic experiences: transnational data from the World Mental Health Survey. *British Journal of Psychiatry*, 211(6):373-380.
129. Mc Laughlin, K.A, Koenen, K.C., Bromet, E.J., Karam, E.G., Liu, H., Petukhova, M., Ruscio, A.M., Sampson, N.A., Stein, D.J., Aguilar-Gaxiola, S., Alonso, J., Borges, G., Demyttenaere, K., Dinolova, R.V., Ferry, F., Florescu, S., de Girolamo, G., Gureje, O., Kawakami, N., Lee, S., Navarro-Mateu, F., Piazza, M., Pennell, B.E., Posada-Villa, J., ten Have, M., Viana, M.C., Kessler, R.C., on behalf of the WHO World Mental Health Survey Collaborators. (2017). Childhood adversities and post-traumatic stress disorder: evidence for stress sensitisation in the World Mental Health Surveys.*British Journal of Psychiatry*, 211(5):280-288.
130. Mojtabai, R., Stuart, E.A., Hwang, I., Eaton, W.W., Sampson, N., Kessler, R.C. (2017). Long-term effects of mental disorders on marital outcomes in the National Comorbidity Survey ten-year follow-up. *Social Psychiatry and Psychiatric Epidemiology,* 52(10): 1217-1226.
131. Mortier, P., Kiekens, G., Auerbach, R.P., Cuijpers, P., Demyttenaere, K., Green, J.G., Kessler, R.C., Nock, M.K, Zaslavsky, A.M., Bruffaerts, R. (2017). A Risk Algorithm for the Persistence of Suicidal Thoughts and Behaviors during College. *Journal of Clinical Psychiatry,*78(7):e828-e836.
132. Mortier, P., Demyttenaere, K., Auerbach, R.P., Cuijpers, P., Green, J.G., Kiekens, G., Kessler, R.C., Nock, M.K., Zaslavsky, A., Bruffaerts, R. (2017). First onset of suicidal thoughts and behaviours in college. *Journal of Affective Disorders*, 207, 291-299
133. Navarro-Mateu, F., Alonso, J., Lim, C.C.W., Saha, S., Aguilar-Gaxiola, S., Al-Hamzawi, A., Andrade, L.H., Bromet, E.J., Bruffaerts, R., Chatterji, S., Degenhardt, L., de Girolamo, G., de Jonge, P., Fayyad, J., Florescu, S., Gureje, O., Haro, J.M., Hu, C., Karam, E.G., Kovess-Masfety, V., Lee, S., Medina-Mora., M.E., Ojagbemi, A., Pennell, B.E., Piazza, M., Posada-Villa, J., Scott, K. M., Stagnaro, J.C., Xavier, M., Kendler, K.S., Kessler, R.C., McGrath, J.J., on behalf of the WHO World Mental Health Survey Collaborators. (2017).The association between psychotic experiences and disability: Results from the WHO World Mental Health Surveys. *Acta Psychiatrica Scandinavica*, 136(1), 74-84.
134. Navarro-Mateu, F., Salmerón, D., Vilagut, G., Tormo, M.J., Ruíz-Merino, G., Escámez, T., Júdez, J., Martínez, S., Koenen, K.C., Navarro, C., Alonso, J., Kessler, R.C. (2017). Post-Traumatic Stress Disorder and other mental disorders in the general population after Lorca's earthquakes, 2011 (Murcia, Spain): A cross-sectional study. *PLOS One,*12(7), e0179690.
135. Pereira, F.G., França, M.H., Paiva, M.C.A., Andrade, L.H., Viana, M.C. (2017). Prevalence and clinical profile of chronic pain and its association with mental disorders. *Revista de Saúde Pública*, 51:96
136. Roest, A.M., De Jonge, P., Lim, C.W., Stein, D.J., Al-Hamzawi, A., Alonso, J., Benjet, C., Bruffaerts, R., Bunting, B., Caldas-de-Almeida, J., Ciutan, M., De Girolamo, G., Hu, C., Levinson, D., Nakamura, Y., Navarro-Mateu, F., Piazza, M., Posada-Villa, J., Torres, Y., Wojtyniak, B., Kessler, R.C., Scott, K.M. (2017). Fear and distress disorders as predictors of heart disease: a temporal perspective. *Journal of Psychosomatic Research*, 96:67-75
137. Ruscio, A.M., Hallion, L.S., Lim, C.C.W., Aguilar-Gaxiola, S., Al-Hamzawi, A., Alonso, J., Andrade, L.H., Borges, G., Bromet, E.J., Bunting, B., Caldas de Almeida, J.C., Demyttenaere, K., Florescu, S., de Girolamo, G., Gureje, O., Haro, J.M., He, Y., Hinkov, H., Hu, C., de Jonge, P., Karam, E., Lee, S., Lepine, J.P., Levinson, D., Mneimneh, Z., Navarro-Mateu, F., Posada-Villa, J., Slade, T., Stein, D.J., Torres, Y., Uda, H., Wojtyniak, B., Kessler, R.C., Chatterji, S., Scott, K. (2017). Cross-Sectional Comparison of the Epidemiology of DSM-5 Generalized Anxiety Disorder Across the Globe. *JAMA Psychiatry*, 74(5), 465-475.
138. Silove, D., Baker, J.R., Mohsin, M. , Teesson, M., Creamer, M., O'Donnell, M. , Forbes, D., Carragher, N., Slade, T., Mills, K., Bryant, R., McFarlane, A., Steel, Z., Felmingham, K., Rees, S. (2017) The contribution of gender-based violence and network trauma to gender differences in Post-Traumatic Stress Disorder. *PLOS One*,12(2), e0171879.
139. Stein, D.J., Lim, C.C.W., Roest, A.M., de Jonge, P., Aguilar-Gaxiola, S., Al-Hamzawi, A., Alonso, J., Benjet, C., Bromet, E.J., Bruffaerts, R., de Girolamo, G., Florescu, S., Gureje, O., Haro, J.M., Harris, M. G., He, Y., Hinkov, H., Horiguchi, I., Hu, C., Karam, A., Karam, E.G., Lee, S., Leacutepine, J.P., Navarro-Mateu, F., Pennell, B.E., Piazza, M., Posada-Villa, J., ten Have, M., Torres, Y., Viana, M.C., Wojtyniak, B., Xavier, M., Kessler, R.C., Scott, K., WHO World Mental Health Survey collaborators. (2017). The cross-national epidemiology of social anxiety disorder: Data from the World Mental Health Survey Initiative. *BMC Medicine,*15(1), 143.
140. Stein, D.J., Scott, K.M., de Jonge, P., Kessler, R.C. (2017). Epidemiology of anxiety disorders: From surveys to nosology and back. *Dialogues in Clinical Neuroscience,*19(2). 127-136.
141. Aguilar-Gaxiola, S., Loera, G., Geraghty, E.M., Ton, H., Lim, C.C., de Jonge, P., Kessler, R.C., Posada-Villa, J., Medina-Mora, M.E., Hu, C., Fiestas, F., Bruffaerts, R., Kovess-Masféty, V., Al-Hamzawi, A.O., Levinson, D., de Girolamo, G., Nakane, Y., ten Have, M., O'Neill, S., Wojtyniak, B., Caldas de Almeida, J.M., Florescu, S., Haro, J.M., Scott, K.M. (2016). Associations between DSM-IV mental disorders and subsequent onset of arthritis. *Journal of Psychosomatic Research*, 82, 11-16.
142. Auerbach, R.P., Alonso, J., Axinn, W.G., Cuijpers, P., Ebert, D.D., Greif Green, J., Hwang, I., Kessler, R.C., Liu, H., Mortier, P., Nock, M.K., Pinder-Amaker, S., Sampson, N.A., Aguilar-Gaxiola, S., Al-Hamzawi, A., Andrade, L.H., Benjet, C., Caldas-de-Almeida, J.M., Demyttenaere, K., Florescu, S., de Girolamo, G., Gureje, O., Haro, J.M., Karam, E.G., Kiejna, A., Kovess-Masfety, V., Lee, S., McGrath, J.J., O'Neill, S., Pennell, B.-E., Scott, K., ten Have, M., Torres, Y., Zaslavsky, A.M., Zarkov, Z., Bruffaerts, R. (2016). Mental disorders among college students in the WHO World Mental Health Surveys. *Psychological Medicine,*46(14), 2955-2970.
143. Benjet, C., Bromet, E., Karam, E.G., Kessler, R.C., McLaughlin, K.A., Ruscio, A.M., Shahly, V., Stein, D.J., Petukhova,M., Hill, E., Alonso, J., Atwoli, L., Bunting, B., Bruffaerts, R., Caldas-de-Almeida, J.M., de Girolamo, G., Florescu, S., Gureje, O., Huang, Y., Lepine, J.P., Kawakami, N., Kovess-Masfety, V., Medina-Mora, M.E., Navarro-Mateu, F., Piazza, M., Posada-Villa, J., Scott, K.M., Shalev, A., Slade, T., Ten Have, M., Torres, Y., Viana, M.C., Zarkov, Z., Koenen, K.C. (2016). The epidemiology of traumatic event exposure worldwide: results from the World Mental Health Survey Consortium. *Psychological Medicine*, 46(2), 327-43.
144. Blasco, M.J.,Castellví , P., Almenara, J., Lagares, C., Roca, M., Sesé, A., Piqueras, J.A., Soto-Sanz, V., Rodrĩguez-Marín, J.,Echeburúa, E., Gabilondo, A., Cebrià, A.I., Miranda-Mendizábal, A., Vilagut, G., Bruffaerts, R., Auerbach, R.P., Kessler, R.C., Alonso, J. and on behalf of the UNIVERSAL study group. (2016). Predictive models for suicidal thoughts and behaviors among Spanish University students: Rationale and methods of the UNIVERSAL (University & mental health) project. *BMC Psychiatry*, 16, 122.
145. Cuijpers, P., Cristea, I.A., Ebert, D.D., Koot, H.M., Auerbach, R.P., Bruffaerts, R., Kessler, R.C. (2016). Psychological treatment of depression in college students: A meta-analysis. *Depression and Anxiety*, 33(5), 400-14
146. de Jonge, P., Roest, A.M., Lim, C.C., Florescu, S.E., Bromet, E.J., Stein, D.J., Harris, M., Nakov, V., Caldas-de-Almeida, J.M., Levinson, D., Al-Hamzawi, A.O., Haro, J.M., Viana, M.C., Borges, G., O'Neill, S., de Girolamo, G., Demyttenaere, K., Gureje, O., Iwata, N., Lee, S., Hu, C., Karam, A., Moskalewicz, J., Kovess-Masfety, V., Navarro-Mateu, F., Browne, M.O., Piazza, M., Posada-Villa, J., Torres, Y, Ten Have, M.L., Kessler, R.C., Scott, K.M. (2016). Cross-national epidemiology of panic disorder and panic attacks in the world mental health surveys.*Depression and Anxiety,*33(12), 1155- 1177.
147. Karam, G., Itani, L., Fayyad, J., Karam, A., Mneimneh, Z., Karam, E. (2016). Prevalence, Correlates, and Treatment of Mental Disorders among Lebanese Older Adults: A National Study. *American Journal of Geriatric Psychiatry*; 24(4), 278-86.
148. Kiekens, G., Claes, L., Demyttenaere, K., Auerbach, R.P., Green, J.G., Kessler, R.C., Mortier, P., Nock, M.K., Bruffaerts, R. (2016). Lifetime and 12-month non-suicidal self-injury and academic performance in college freshmen. *Suicide and Life-Threatening Behavior*, 46(5), 563-576.
149. Lubin, G., Barash, I., Levinson, D. (2016). Combat experience and mental health in the Israel national health survey. *Israel Journal of Psychiatry and Related Sciences*, 53(3), 3-8.
150. McGrath, J.J., Saha, S., Al-Hamzawi, A.O., Alonso, J., Andrade, L., Borges, G., Bromet, E.J., Oakley Browne, M., Bruffaerts, R., Caldas de Almeida, J.M., Fayyad, J., Florescu, S., de Girolamo, G., Gureje, O., Hu, C., de Jonge, P., Kovess-Masfety, V., Lepine, J.P., Lim, C.C., Navarro-Mateu, F., Piazza, M., Sampson, N., Posada-Villa, J., Kendler, K.S., Kessler, R.C. (2016). Age of onset and lifetime projected risk of psychotic experiences: Cross-national data from the World Mental Health Survey. *Schizophrenia Bulletin,*42(4), 933-941.
151. McGrath, J.J., Saha, S., Al-Hamzawi, A., Andrade, L., Benjet, C., Bromet, E.J., Browne, M.O., Caldas de Almeida, J.M., Chiu, W.T., Demyttenaere, K., Fayyad, J., Florescu, S., de Girolamo, G., Gureje, O., Haro, J.M., ten Have, M., Hu, C., Kovess-Masfety, V., Lim, C.C., Navarro-Mateu, F., Sampson, N., Posada-Villa, J., Kendler, K.S., Kessler, R.C. (2016). The bidirectional associations between psychotic experiences and DSM-IV mental disorders. *The American Journal of Psychiatry*, 173 (10), 997-1006.
152. McLafferty, M., Armour, C., O'Neill. S., Murphy. S., Ferry, F., Bunting, B. (2016). Suicidality and profiles of childhood adversities, conflict related trauma and psychopathology in the Northern Ireland population. *Journal of Affective Disorders*, 200, 97-102
153. Scott, K.M., Lim, C.C.W., Al-Hamzawi, A., Alonso, J., Bruffaerts, R., Caldas-de-Almeida, J.M., Florescu, S., de Girolamo, G., Hu, C., de Jonge, P., Kawakami, N., Medina-Mora, ME., Moskalewicz, J., Navarro-Mateu, F., O'Neill, S., Piazza, M., Posada-Villa, J., Torres, Y., Kessler, R.C. (2016). Association of Mental Disorders With Subsequent Chronic Physical Conditions: World Mental Health Surveys From 17 Countries. *JAMA Psychiatry*, 73(2), 150-8
154. Scott, K.M., Lim, C.W., Hwang, I., Adamowski, T., Al-Hamzawi, A. Bromet, E.J, Bunting, B., Piazza Ferrand, M., Florescu, S., Gureje, O., Hinkov, H., Hu, C., Karam, E.G, Lee, S., Posada-Villa, J., Stein, D. J., Tachimori, H., Viana, M.C., Xavier, M., Kessler, R.C. (2016). The cross-national epidemiology of DSM IV Intermittent Explosive Disorder. *Psychological Medicine*, 46(15), 3161-3172.
155. Slade, T., Chiu, W.T., Glantz, M., Kessler, R.C., Lago, L., Sampson, N., Al-Hamzawi, A., Florescu, S., Moskalewicz, J., Murphy, S., Navarro-Mateu, F., Torres de Galvis, Y., Viana, M.C., Xavier, M., Degenhardt, L. (2016). A Cross-National Examination of Differences in Classification of Lifetime Alcohol Use Disorder Between DSM-IV and DSM-5: Findings from the World Mental Health Survey. *Alcoholism Clinical and Experimental Research*, 40(8), 1728-36.
156. Stein, D. J., Karam, E.G, Shahly, V., Hill, E.D, King, A., Petukhova, M., Atwoli, L., Bromet, E.J., Florescu, S., Haro, J.M., Hinkov, H., Karam, A., Medina-Mora, M., Navarro-Mateu, F., Piazza, M., Shalev, A., Torres, Y., Zaslavsky, A.M., Kessler, R.C. (2016) Post-traumatic stress disorder associated with life-threatening motor vehicle collisions in the WHO World Mental Health Surveys. *BMC Psychiatry*. 16, 257
157. Uwakwe, R., Gureje, O. Sociodemographic correlates of continuing tobacco use - a descriptive report from the Nigerian Survey of Mental Health and Wellbeing. (2016). *Acta Psychiatrica Scandinavica*, 133(6), 506-13.
158. Al-Hamzawi, A., Bruffaerts, R., Bromet, E. J., AlKhafaji, A.M., Kessler, R.C. (2015). The epidemiology of major depressive episode in the Iraqi general population. *PLoS One*, 10(7), e0131937.
159. Axinn, W.G., Ghimire, D.J., Williams, N.E., Scott, K.M. (2015). Associations between the social organization of communities and psychiatric disorders in rural Asia. *Social Psychiatry and Psychiatric Epidemiology*, 50(10), 1537-45.
160. Birrell, L., Newton, N.C., Teesson, M., Tonks, Z., Slade, T. Anxiety disorders and first alcohol use in the general population. Findings from a nationally representative sample. (2015) *Journal of anxiety disorders*. 31:108-13.
161. Boyd, A., Van de Velde, S., Pivette, M., Ten Have, M., Florescu, S., O'Neill, S., Caldas-de-Almeida, J.M., Vilagut, G., Haro, J.M., Alonso, J., Kovess-Masfety, V., and the EU-WMH Investigators. (2015). Gender differences in psychotropic use across Europe: Results from a large cross-sectional, population-based study. *European Psychiatry*, 30(6), 778-88.
162. Boyd, A., Van de Velde, S., Vilagut, G., de Graaf, R., O'Neill, S., Florescu, S., Alonso, J., Kovess-Masfety, V., and the EU-WMH Investigators. (2015). Gender differences in mental disorders and suicidality in Europe: Results from a large cross-sectional population-based study. *Journal of Affective Disorders*. 173: 245-254
163. Byrne, G.J, Steele, S.J., Pachana, N.A. (2015) Delusion-like experiences in older people with anxiety disorders. *International psychogeriatrics*. 27(7):1191-6.
164. Bruffaerts, R., Demyttenaere, K., Kessler, R.C., Tachimori, H., Bunting, B., Hu, C., Florescu, S., Haro, J.M., Lim, C.W., Kovess-Masfety, V., Levinson, D., Medina Mora, M.E., Piazza, M., Piotrowski, P., Posada-Villa, J., Salih Khalaf, M., ten Have, M., Xavier, M., Scott, K.M. (2015). The associations between pre-existing mental disorders and subsequent onset of chronic headaches: A worldwide epidemiological perspective. *The Journal of Pain*. 16(1):42-52.
165. Bruffaerts, R., Kessler, R.C., Demyttenaere, K., Bonnewyn, A., Nock, M. K. (2015). Examination of the population attributable risk of different risk factor domains for suicidal thoughts and behaviors. *Journal of Affective Disorders*, 187, 66-72.
166. Fayyad, J., Kessler, R.C. (2015). The epidemiology and societal burden of ADHD. In: Adler, L., Spencer T., Wilens, T.E. (Eds.), Attention-Deficit Hyperactivity Disorder in Adults and Children (pp 24-41). *Cambridge University Press*.
167. Green, J.G., Alegría, M., M., Kessler, R.C., McLaughlin, K.A., Gruber, M.J., Sampson, N.A., Zaslavsky, A. (2015). Neighborhood Sociodemographic Predictors of Serious Emotional Disturbance (SED) in Schools: Demonstrating a Small Area Estimation Method in the National Comorbidity Survey (NCS-A) *Adolescent Supplement. Administration and Policy in Mental Health*. 42(1):111-20.
168. Husky, M.M., Lépine, J.-P., Gasquet, I., Kovess-Masfety, V. (2015). Exposure to traumatic events and posttraumatic stress disorder in France: Results from the WMH Survey. *Journal of Traumatic Stress*, 28(4), 275-82.
169. Karam, E.G., Itani, I., Fayyad, J., Hantouche, E., Karam, A., Mneimneh, Z., Akiskal, H., Rihmer, Z. (2015). Temperament and suicide: A national study. *Journal of Affective Disorders*, 184: 123-128.
170. Kessler, R.C., Sampson, N.A., Berglund. P, Gruber, M.J., Al-Hamzawi, A., Andrade, L., Bunting, B., Demyttenaere, K., Florescu, S., de Girolamo, G., Gureje, O., He, Y., Hu, C., Huang, Y., Karam, E., Kovess-Masfety, V., Lee, S., Levinson, D., Medina Mora, M.E., Moskalewicz, J., Nakamura, Y., Navarro-Mateu, F., Browne, M.A., Piazza, M., Posada-Villa, J., Slade, T., Ten Have, M., Torres, Y., Vilagut, G., Xavier, M., Zarkov, Z., Shahly, V., Wilcox, M.A. (2015) Anxious and non-anxious major depressive disorder in the World Health Organization World Mental Health Surveys. *Epidemiology and psychiatric sciences*. 24(3):210-26.
171. Kiejna, A., Piotrowski, P., Adamowski, T., Moskalewicz, J., Wciórka, J., Stokwiszewski, J., Rabczenko, D., Kessler, R.C. (2015). The prevalence of common mental disorders in the population of adult Poles by sex and age structure - an EZOP Poland study. *Psychiatria Polska*, 49(1), 15-27.
172. Kiejna, A., Piotrowski, P., Adamowski, T., Moskalewicz, J., Wciórka, J., Stokwiszewski, J., Rabczenko, D., Kessler, R.C. (2015). The prevalence of common mental disorders in the population of adult Poles by sex and age structure - an EZOP Poland study. *Psychiatria Polska*. 49(1): 15-27.
173. McGrath, J.J., Saha, S., Al-Hamzawi, A., Alonso, J., Bromet, E.J., Bruffaerts, R., Caldas-de-Almeida, J.M., Chiu, W.T., de Jonge, P., Fayyad, J., Florescu, S., Gureje, O., Haro, J.M., Hu, C., Kovess-Masfety, V., Lepine, J.P., Lim, C.C., Mora, M.E., Navarro-Mateu, F., Ochoa , S., Sampson , N.A., Scott , K., Viana, M.C., Kessler, R.C. (2015). Psychotic experiences in the general population: A cross-national analysis based on 31,261 respondents from 18 countries. *JAMA Psychiatry*, 72(7), 697-705.
174. McLafferty, M., Armour, C., McKenna, A., O’Neill, S., Murphy, S., & Bunting, B. (2015). Childhood adversity profiles and adult psychopathology in a representative Northern Ireland study. *Journal of Anxiety Disorders*, 35, 42-48.
175. McLaughlin, K.A., Koenen, K.C., Friedman, M.J., Ruscio, A.M., Karam, E.G., Shahly, V., Stein, D.J., Hill, E.D., Petukhova, M., Alonso, J., Andrade, L.H., Angermeyer, M.C., Borges, G., de Girolamo, G., de Graaf, R., Demyttenaere, K., Florescu, S.E., Mladenova, M., Posada-Villa, J., Scott, K.M., Takeshima, T., Kessler, R.C. (2015). Subthreshold Posttraumatic Stress Disorder in the World Health Organization World Mental Health Surveys. *Biological Psychiatry*. 77(4):375-84.
176. Mojtabai, R., Stuart, E.A., Hwang, I., Eaton, W.W., Sampson, N., Kessler, R.C. (2015). Long-term effects of mental disorders on educational attainment in the National Comorbidity Survey ten-year follow-up. *Social Psychiatry and Psychiatric Epidemiology*, 50(10), 1577-9
177. Mortier, P., Demyttenaere, K., Auerbach, R.P., Green, J.G., Kessler, R.C., Kiekens, G., Nock, M.K., Bruffaerts, R. (2015). The impact of lifetime suicidality on academic performance in college freshmen. *Journal of Affective Disorders*, 186, 254-260.
178. Mortier, P., Demyttenaere, K., Nock, M.K., Green, J.G., Kessler, R.C., Bruffaerts, R. (2015) [The epidemiology of ADHD in first-year university students] (article in Dutch). *Tijdschrift voot Psychiatrie*. 57(9):635-44.
179. Navarro-Mateu, F., Tormo, M.J., Salmerón, D., Vilagut, G., Navarro, C., Ruíz-Merino, G., Escámez, T., Júdez, J., Martínez, S., Kessler, R.C., Alonso, J. (2015). Prevalence of mental disorders in the South-East of Spain, one of the European regions most affected by the economic crisis: The cross-sectional PEGASUS-Murcia Project. *PLoS One*, 10(9), e0137293.
180. Olaya, B., Alonso, J., Atwoli, L., Kessler, R.C., Vilagut, G., Haro, J.M. (2015). Association between traumatic events and post-traumatic stress disorder: results from the ESEMeD-Spain study. Epidemiology and Psychiatric Sciences, 24(2): 172-183.
181. Rapsey, C.M., Lim, C.C.W., Al-Hamzawi, A., Alonso, J., Bruffaerts, R., Caldas-de-Almeida, J.M., Florescu, S., de Girolamo, G., Hu, C., Kessler, R.C., Kovess-Masfety, V., Levinson, D., Medina-Mora, M.E., Murphy, S., Ono, Y., Piazza M., Posada-Villa, J., ten Have, M., Wojtyniak, B., Scott, K.M. (2015). Associations between DSM-IV mental disorders and subsequent COPD diagnosis. *Journal of Psychosomatic Research*, 79(5), 333-9.
182. Santana, G.L., Coelho, B.M., Borges, G., Viana, M.C., Wang, Y.P., Andrade, L.H. (2015). The influence of parental psychopathology on offspring suicidal behavior across the lifespan. *PLoS One*, 10(7), e0134970.
183. Silove, D., Alonso, J., Bromet, E., Gruber, M., Sampson, N., Scott, K., Andrade, L., Benjet, C., Caldas de Almeida, J.M., de Girolamo, G., de Jonge, P., Demyttenaere, K., Fiestas, F., Florescu, S., Gureje, O., He., Y., Karam, E., Lepine, J.P., Murphy, S., Villa-Posada, J., Zarkov, Z., Kessler, R.C. (2015). Pediatric-onset and adult-onset separation anxiety disorder across countries in the World Mental Health Survey. *American Journal of Psychiatry*, 172(7), 647-56.
184. Slade, T., McEvoy, P.M., Chapman, C., Grove, R., Teesson, M. (2015) Onset and temporal sequencing of lifetime anxiety, mood and substance use disorders in the general population. *Epidemiology and Psychiatric Sciences*, 24(1):45-5
185. Sunderland, M., Slade, T., Krueger, R.F. (2015). Examining the shared and unique relationships among substance use and mental disorders. *Psychological Medicine*. 45(5):1103-13.
186. Swain, N.R., Lim, C.C., Levinson, D., Fiestas, F., De Girolamo, G., Moskalewicz, J., Lepine, J.-P., Posada-Villa, J., Haro, J.M., Medina-Mora, M.E., Xavier, M., Iwata, N., De Jonge, P., Bruffaerts, R., O'Neill, S., Kessler, R. C., Scott, K.M. (2015). Associations between DSM-IV mental disorders and subsequent non-fatal, self-reported stroke. *Journal of Psychosomatic Research*, 79(2), 130-6.
187. Swanson, J.W., Sampson, N.A., Petukhova, M.V., Zaslavsky, A.M.,, Appelbaum, P.S., Swartz, M.S., Kessler, R.C. (2015) Guns, Impulsive Angry Behavior, and Mental Disorders: Results from the National Comorbidity Survey Replication (NCS-R). *Behavioral sciences & the law*. 33(2-3):199-212.

DUPLICATES

1. Bruffaerts, R., Posada-Villa, J., Al-Hamzawi, A.O., Gureje, O., Huang, Y., Hu, C., Bromet, E.J., Viana, M.C., Hinkov, H.R., Karam, E.G., Borges, G., Florescu, S.E., Williams, D.R., Demyttenaere, K., Kovess-Masfety, V., Matschinger, H., Levinson, D., De Girolamo, G., Ono, Y., de Graaf, R., Oakley Browne, M., Bunting, B., Xavier, M., Haro, J.M., Kessler, R.C. (2015). Proportion of patients without mental disorders being treated in mental health services worldwide. *British Journal of Psychiatry*. 206(2):101-9.
2. Harris, M., Kazdin, A.E., Chiu, W.T., Sampson, N.A., Aguilar-Gaxiola, S., Al-Hamzawi, A., Alonso, J., Altwaijri, Y., Andrade, L.H., Cardoso, G., Cía, A., Florescu, S., Gureje, O., Hu, C., Karam, E.G., Karam, G., Mneimneh, Z., Navarro-Mateu, F., Oladeji, B., O'Neill, S., Scott, K., Slade, T., Torres, Y., Vigo, D., Wojtyniak, B., Zarkov, Z., Ziv, Y., Kessler, R.C., on behalf of the WHO World Mental Health Survey Collaborators. (2020). Findings From World Mental Health Surveys of the Perceived Helpfulness of Treatment for Patients With Major Depressive Disorder. *JAMA Psychiatry*,77(8):830-841.
3. Harris, M. G., Kazdin, A. E., Munthali, R. J., Vigo, D. V., Hwang, I., Sampson, N. A., Al-Hamzawi, A., Alonso, J., Andrade, L. H., Borges, G., Bunting, B., Florescu, S., Gureje, O., Karam, E. G., Lee, S., Navarro-Mateu, F., Nishi, D., Rapsey, C., Scott, K. M., Stagnaro, J. C., Viana, M. C., Wojtyniak, B., Xavier, M., & Kessler, R. C. (2022). Perceived helpfulness of service sectors used for mental and substance use disorders: Findings from the WHO World Mental Health Surveys. *International Journal of Mental Health Systems*, 16(1), 6.
4. Silva, M., Antunes, A., Azeredo-Lopes, S., Cardoso, G., Xavier, M., Saraceno, B., Caldas de Almeida, J.M. (2022). Barriers to mental health services utilization in Portugal: results from the National Mental Health Survey. *Journal of Mental Health*, 31(4):453-461.
5. Thornicroft, G., Chatterji , S., Evans-Lacko, S., Gruber, M., Sampson, N., Aguilar-Gaxiola , S., Al-Hamzawi , A., Alonso, A., Andrade, L., Borges, G., Bruffaerts , R., Bunting, B., Caldas de Almeida, J.M., Florescu, S., de Girolamo, G., Gureje, O., Haro, J.M, He, Y., Hinkov, H., Karam, E., Kawakami, N., Lee, S., Navarro-Mateu, F., Piazza, M., Posada-Villa, J., Torres de Galvis, Y., Kessler, R.C. (2017). Undertreatment of people with major depressive disorder in 21 countries. *British Journal of Psychiatry,*210(2), 119-124.
6. Commentary: Shaw, P., Ahn, K., Rapoport, J.L. (2017). Good news for screening for Adult Attention-Deficit/Hyperactivity Disorder. *JAMA Psychiatry*, 74(5), 527.
7. Wardenaar, K.J., Lim, C.W., Al-Hamzawi, A., Alonso, J., Andrade, L.H., Benjet, C., Bunting, B., de Girolamo, G., Demyttenaere, K., Florescu, S., Gureje, O., Hisateru, T., Hu, C., Huang, Y., Karam, E., Kiejna, A., Lepine, J.P., Navarro-Mateu, F., Oakley Browne, M., Piazza, M., Posada-Villa, J., ten Have, M.L., Torres, Y., Xavier, M., Zarkov, Z., Kessler, R.C., Scott, K.M., de Jonge, P. (2017). The cross-national epidemiology of specific phobia in the World Mental Health Surveys. *Psychological Medicine*, 47(10), 1744-1760.
8. Bruffaerts, R., Posada-Villa, J., Al-Hamzawi, A.O., Gureje, O., Huang, Y., Hu, C., Bromet, E.J., Viana, M.C., Hinkov, H.R., Karam, E.G., Borges, G., Florescu, S.E., Williams, D.R., Demyttenaere, K., Kovess-Masfety, V., Matschinger, H., Levinson, D., De Girolamo, G., Ono, Y., de Graaf, R., Oakley Browne, M., Bunting, B., Xavier, M., Haro, J.M., Kessler, R.C. (2015). Proportion of patients without mental disorders being treated in mental health services worldwide. *British Journal of Psychiatry*. 206(2):101-9
9. Kiejna, A., Adamowski, T., Piotrowski, P., Moskalewicz, J., Wojtyniak, B., Swiatkiewicz, G., Stokwiszewski, J., Kantorska-Janiec, M., Zagdanska, M., Kessler, R.C. (2015). Epidemiology of mental disorders and access to mental health care. EZOP - Poland - research methodology. *Psychiatria Polska*. 49(1): 5-13.

# **Table S1. Characteristics of included studies**

| **Country** | **Study ID** | **Representativeness** | **Study design** | **Population subgroup** | **Mental Disorder** | **Calendar Year** | **Sample Size** | **Age** | **Mental Health Coverage or Treatment gap** |
| --- | --- | --- | --- | --- | --- | --- | --- | --- | --- |
| Albania | Santomauro 2024 - Albania | nationally representative | Cross-sectional: World Mental Health Surveys report | None specified | major depressive disorder | 2000 and 2021 | 42200 (95%UI 2000) 58300 (95%UI 2021) | >= 18 y | Proportion of persons with major depressive disorder receiving minimally adequate treatment by sex and location for 2000 and 2021 (95%UI 95% UIs): 12·4 (95%UI 9·5–17·0) and 13·6 (95%UI 10·4–18·2) |
| Andorra | Santomauro 2024 - Andorra | nationally representative | modelling study | None specified | major depressive disorder | 2000 and 2021 | 2000 (95%UI 2000) 3300 (95%UI 2021) | >= 18 y | Proportion of persons with major depressive disorder receiving minimally adequate treatment by sex and location for 2000 and 2021 (95%UI 95% UIs): 27·2 (95%UI 19·1–39·3) and 27·7 (95%UI 19·7–40·0) |
| Armenia | Santomauro 2024 - Armenia | nationally representative | modelling study | None specified | major depressive disorder | 2000 and 2021 | 62400 (95%UI 2000) 91400 (95%UI 2021) | >= 18 y | Proportion of persons with major depressive disorder receiving minimally adequate treatment by sex and location for 2000 and 2021 (95%UI 95% UIs): 10·8 (95%UI 7·9–15·0) and 12·0 (95%UI 8·7–16·6) |
| Austria | Santomauro 2024 - Austria | nationally representative | modelling study | None specified | major depressive disorder | 2000 and 2021 | 196800 (95%UI 2000) 246400 (95%UI 2021) | >= 18 y | Proportion of persons with major depressive disorder receiving minimally adequate treatment by sex and location for 2000 and 2021 (95%UI 95% UIs): 25·8 (95%UI 18·6–36·4) and 26·7 (95%UI 19·5–37·5) |
| Azerbaijan | ATLAS -Azerbaijan | nationally representative | ATLAS | unclear | Psychosis | 2017 | 10047719 | unclear | service coverage for psychosis 2017 and 2020 % = 45·12 |
|  | Santomauro 2024 - Azerbaijan | nationally representative | modelling study | None specified | major depressive disorder | 2000 and 2021 | 124300 (95%UI 2000) 252700 (95%UI 2021) | >= 18 y | Proportion of persons with major depressive disorder receiving minimally adequate treatment by sex and location for 2000 and 2021 (95%UI 95% UIs): 9·6 (95%UI 6·8–13·6) and 11·0 (95%UI 7·9–15·9) |
| Belarus | ATLAS -Belarus | nationally representative | ATLAS | unclear | Psychosis | 2017 and 2020 | 9452409 | unclear | service coverage for psychosis 2017 and 2020 % = 55·9 and 64·04 |
|  | Santomauro 2024 - Belarus | nationally representative | modelling study | None specified | major depressive disorder | 2000 and 2021 | 380500 (95%UI 2000) 461800 (95%UI 2021) | >= 18 y | Proportion of persons with major depressive disorder receiving minimally adequate treatment by sex and location for 2000 and 2021 (95%UI 95% UIs): 11·8 (95%UI 8·7–16·7) and 13·5 (95%UI 9·8–19·1) |
| Belgium | Alonso 2018 - Belgium | nationally representative | cross sectional - WHO World Mental Health Survey Initiative | None specified | anxiety (95%UI agoraphobia, generalized anxiety disorder, panic disorder, PTSD, social phobia, specific phobia, adult separation anxiety disorder)· | 2001–2002 | 2419 | 18–95 y | Any treatment among those with disorder % (95%UI se); possibly adequate treatment % (95%UI se)= 35·7 (95%UI 3·8) ; 11·2 (95%UI 2·9) |
|  | de Vries 2021- Belgium | nationally representative | cross-sectional: World Mental Health Surveys report | None specified | specific phobia | 2001-2002 | all participants (95%UI not only diagnosed)=1043 | 18-95 y | Respondents who obtained treatment for specific phobia %(95%UI se): 37·6 (95%UI 16·3) |
|  | Degenhard 2017 - Belgium | nationally representative | cross sectional - WHO World Mental Health Survey Initiative | None specified | Substance use disorders | 2001-2002 | 2419 | 18-95 y | Minimally adequate treatment among allthose with substanceuse disorders % (95%UI se) = 6·8 (95%UI 1·5) |
|  | Fayyad 2017 - Belgium | nationally representative | cross sectional - WHO World Mental Health Survey Initiative | None specified | ADHD | 2001-2002 | 2419 | 18-97 y | Twelve-month treatment (95%UI any mental health treatment) among respondents with multiply imputed DSM-IV/CIDI adult attention-deficit hyperactivity disorder % (95%UI se)= 0·0 (95%UI 0·0) |
|  | Kadzin 2023- Belgium | nationally representative | Cross-sectional: World Mental Health Surveys report | None specified | major depressive disorder, anxiety disorders | 2001-2002 | 2419 | 18-95 y | Prevalence of ADM use by MDD and anxiety disorder histories (95%UI 12-month MDD), % (95%UI SE)= 41·1 (95%UI 7·0) and 21·9 (95%UI 5·2) |
|  | Rens 2022 | Antwerp province (95%UI urban and rural zones) | Cross-sectional | Ethnic minorities and low-income individuals with unmet needs | Depression, anxiety, alcohol abuse | May- July 2021 | 1208 | mean age 45·5 years old (95%UI SD = 17·8)· Females 49·8% | 47·6% (95%UI n=69) people with clinically met needs· 52·4% (95%UI 66) people with clincal need for mental health reasons who did not use health care for mental health· |
|  | Santomauro 2024 - Belgium | nationally representative | modelling study | None specified | major depressive disorder | 2000 and 2021 | 280900 (95%UI 2000) 410000 (95%UI 2021) | >= 18 y | Proportion of persons with major depressive disorder receiving minimally adequate treatment by sex and location for 2000 and 2021 (95%UI 95% UIs): 32·4 (95%UI 24·0–44·1) and 34·4 (95%UI 25·2–47·6) |
|  | Stein 2021- Belgium | nationally representative | cross sectional - WHO World Mental Health Survey Initiative | None specified | GAD (95%UI generalized anxiety disorder) | 2001-2002 | all participants (95%UI not only diagnosed)=1043 | 18-95 y | Proportion of Respondents with lifetime GAD, who obtained treatment % (95%UI se)= 35·6 (95%UI 12·5) |
|  | Stein 2023 -Belgium | nationally representative | Cross-sectional: World Mental Health Surveys report | None specified | PTSD | 2001-2002 | 2419 | 18-95 y | missing |
| Belgium, France, Germany, Italy, The Netherlands and Spain | Demyttenaere 2008 | random sample | In a cross-sectional, population-based study | random sample of non-institutionalised adults | anxiety disorder and painful physical symptoms | January 2001 - July 2003 | 21,425 respondents | >=18 y (95%UI table 1) | respondents with both Anxiety disorders and painful physical symptoms had numerically higher rates of help-seeking for emotional reasons (95%UI 20·8% vs· 15·0% for respondents with AD but not PPS) and delayed their helpseeking for a numerically shorter period of time (95%UI 289 vs· 413 days, respectively)· |
| Bosnia and Herzegovina | ATLAS -Bosnia and Herzegovina | nationally representative | ATLAS | unclear | Psychosis | 2017 and 2020 | 3300998 | unclear | service coverage for psychosis 2017 %= 37·11 |
|  | Santomauro 2024 - Bosnia and Herzegovina | nationally representative | modelling study | None specified | major depressive disorder | 2000 and 2021 | 98600 (95%UI 2000) 95900 (95%UI 2021) | >=18 y | Proportion of persons with major depressive disorder receiving minimally adequate treatment by sex and location for 2000 and 2021 (95%UI 95% UIs): 11·7 (95%UI 8·6–16·6) and 12·8 (95%UI 9·4–17·9) |
| Bulgaria | Alonso 2018 - Bulgaria | nationally representative | cross sectional - WHO World Mental Health Survey Initiative | None specified | anxiety (95%UI agoraphobia, generalized anxiety disorder, panic disorder, PTSD, social phobia, specific phobia, adult separation anxiety disorder)· | 2002-2006 | 5318 | 18-98 y | Any treatment among those with disorder % (95%UI se); possibly adequate treatment % (95%UI se)= 21·6 (95%UI 3·7); 7·3 (95%UI 1·9) |
|  | de Vries 2021 - Bulgaria | nationally representative | cross-sectional: World Mental Health Surveys report | None specified | specific phobia | 2002-2006 | all participants (95%UI not only diagnosed)= 5318 | 18-98 y | Respondents who obtained treatment for specific phobia %(95%UI se): 6·6 (95%UI 1·9) |
|  | de Vries 2021 - Bulgaria b | nationally representative | cross-sectional: World Mental Health Surveys report | None specified | specific phobia | 2016-2017 | all participants (95%UI not only diagnosed)= 1508 | 18-91 y | Respondents who obtained treatment for specific phobia %(95%UI se): 6·6 (95%UI 1·9) |
|  | Degenhard 2017 - Bulgaria | nationally representative | cross sectional - WHO World Mental Health Survey Initiative | None specified | Substance use disorders | 2002-2006 | 5318 | 18-93 y | Minimally adequate treatment among allthose with substanceuse disorders % (95%UI se) = 2·4 (95%UI 0·2) |
|  | Harris 2024 -Bulgaria | nationally representative | cross-sectional: World Mental Health Surveys report | None specified | major depressive disorder, bipolar spectrum disorder, panic disorder/agoraphobia, generalized anxiety disorder, posttraumatic stress disorder, social phobia, specific phobia, and substance use disorders (95%UI alcohol and illicit drug abuse with or without dependence) | 2016-2017 | 578 | 18-91 y | 12-month use of providers for mental health among those with any DSM-IV 12-month disorders: 12·1% (95%UI SE=4·3) |
|  | Kadzin 2023- Bulgaria | nationally representative | Cross-sectional: World Mental Health Surveys report | None specified | major depressive disorder, anxiety disorders | 2002-2006 | 5318 | 18-98 y | Prevalence of ADM use by MDD and anxiety disorder histories (95%UI 12-month MDD), % (95%UI SE)= 5·5 (95%UI 2·3) and 1·6 (95%UI 0·7) |
|  | Santomauro 2024 - Bulgaria | nationally representative | modelling study | None specified | major depressive disorder | 2000 and 2021 | 196300 (95%UI 2000) 20300 (95%UI 2021) | >= 18 y | Proportion of persons with major depressive disorder receiving minimally adequate treatment by sex and location for 2000 and 2021 (95%UI 95% UIs): 8·7 (95%UI 6·4–11·6) and 9·5 (95%UI 6·9–13·0) |
|  | Stein 2020- Bulgaria | nationally representative | Cross-sectional: World Mental Health Surveys report | None specified | PTSD | 2016-2017 | 2811 | 18-98 y | among respondents with lifetime PTSD, % (95%UI SE) obtained treatment = 10·3 (95%UI 2·8) |
|  | Stein 2021- Bulgaria | nationally representative | cross sectional - WHO World Mental Health Survey Initiative | None specified | GAD (95%UI generalized anxiety disorder) | 2002-2006 | all participants (95%UI not only diagnosed)= 5318 | 18-98 y | Proportion of Respondents with lifetime GAD, who obtained treatment % (95%UI se)= 16·8 (95%UI 3·2) |
| Croatia | ATLAS -Croatia | nationally representative | ATLAS | unclear | Psychosis | 2017 and 2020 | 4130299 | unclear | service coverage for psychosis 2017 %= 99·45 |
|  | Santomauro 2024 - Croatia | nationally representative | modelling study | None specified | major depressive disorder | 2000 and 2021 | 127800 (95%UI 2000) 18900 (95%UI 2021) | >= 18 y | Proportion of persons with major depressive disorder receiving minimally adequate treatment by sex and location for 2000 and 2021 (95%UI 95% UIs): 12·8 (95%UI 9·4–18·1) and 13·8 (95%UI 10·1–19·1) |
| Cyprus | Santomauro 2024 - Cyprus | nationally representative | modelling study | None specified | major depressive disorder | 2000 and 2021 | 20300 (95%UI 2000) 38400 (95%UI 2021) | >= 18 y | Proportion of persons with major depressive disorder receiving minimally adequate treatment by sex and location for 2000 and 2021 (95%UI 95% UIs): 22·0 (95%UI 15·6–31·9) and 26·8 (95%UI 19·0–38·7) |
| Czechia | Kagstrom 2019 | Nationally representative survey | Cross-sectional | None specified | Affective disorders, anxiety disorders, alcohol use disorders, substance use disorders | October - November 2017 | 659 participants | mean age 49 years old, 54% females | 17% utilized mental health services in the past year· Total gap 83%; Stratifying by diagnosis, the treatment gap was 61% for affective, 69% for anxiety, 77% for substance use and 93% for alcohol use disorders |
|  | Potocar 2024 | Nationally representative survey | Cross-sectional survey on post-COVID-19 mental health | None specified | Mood disorders, anxiety, substance use disorders | 2017, 2020, 2022 | 7,311 respondents in 2022 | The mean age in 2022 data collection was 48·82 years (95%UI standard deviation (95%UI SD) = 16·57), and the sample included 53·17 % females· | Table 2· prevalence of treatment gap stratified by disorder· When compared with the 2017 pre-pandemic estimates (95%UI 82·22 %; 95 % CI = 79·29–85·15), the treatment gap in people who had at least one of the studied mental disorders was largely unchanged in both 2020 pandemic (95%UI 78·7 %; 76·06–81·35 and 76·02; 73·34–78·69) and 2022 (95%UI 77·49 %; 75·66–79·31) data collections· Almost 60 % of individuals who sought professional help in the last 12 months reported that they were confronted with structural barriers such as long waiting lists and unavailability of services (95%UI 56·97 %; 95 % CI = 53·37–60·56)· |
|  | Sebela 2020 | Nationally representative survey | Cross-sectional study (95%UI subset of CZEMS study) | Females on maternity or parental leave | General mental health, anxiety, alcohol use | October 16 2017- November 20 2017 | 119 females | Females on maternity/parental leave; mean age 30·3 y (95%UI SD = 4·4; median 30; range = 19–43 years | Treatment gap= 76% (95%UI 95%CI 55-89), n=16/21 for any mental disorder· Alcohol use disorder 78% (95%UI 45-94); Agoraphobia 64% (95%UI 35-85) n=7/11, presence of suicidal thoughts 60%(95%UI 23-88) n =3/5 |
|  | ATLAS -Czechia | nationally representative | ATLAS | unclear | Psychosis | 2017 and 2020 | 10689213 | unclear | service coverage for psychosis 2017 %= 94 |
|  | Pitonák 2024 | Czech community-dwelling, representative of the adult population | cross-sectional | sexual minorities focus | any | November and December 2022 | 2917 (95%UI 95·2%) heterosexual, 44 (95%UI 1·44%) gay or lesbian, 48 (95%UI 1·57%) bisexual and 54 (95%UI 1·76%) more sexually diverse individuals· | mean age (95%UI sd)= Heterosexual 49·86 (95%UI 16·76); Gay or lesbian 34·64 (95%UI 12·1); Bisexual 34·81 (95%UI 15·56); More sexually diverse 44·26 (95%UI 18·08)· Female n(95%UI %)= Heterosexual 1587 (95%UI 54·41); Gay or lesbian 19 (95%UI 43·18); Bisexual 33 (95%UI 68·75); More sexually diverse 32 (95%UI 59·26) | table 2· We demonstrated broadly consistent levels of treatment gap in heterosexual and SM individuals fulfilling the criteria for any mental disorder (95%UI 82·91%; 95% CI = 79·50–85·96 vs· 81·13%; 68·03–90·56)· In SM individuals scoring positively for MDE, we detected a wider treatment gap than in heterosexual people (95%UI 82·35%; 56·57–96·20 vs· 60·87%; 52·20–69·06)· |
|  | Santomauro 2024 - Czechia | nationally representative | modelling study | None specified | major depressive disorder | 2000 and 2021 | 264800 (95%UI 2000) 44700 (95%UI 2021) | >= 18 y | Proportion of persons with major depressive disorder receiving minimally adequate treatment by sex and location for 2000 and 2021 (95%UI 95% UIs): 13·1 (95%UI 9·6–18·8) and 14·1 (95%UI 10·2–19·6) |
| Denmark | Aagaard 2004 | 2 regions | Epidemiological analysis: prevalence study using the national Danish Psychiatric Central Register | None specified | severe mental illness | 1981 to 2000 |  | Compared to the average in Denmark, SJ county has a smaller fraction aged 20/30 years· In the Tønder region, relatively more are employed with farming and unskilled work· In the Aabenraa region, relatively more are employed with transportation or as public servants· The distribution of gender and age groups was equal between the Tønder and Aabenraa region | Rate of inactive patients is 0·28/1000· Prevalence rate of SMI is 1·31/1000 · Crude coverage rate is the inverse of this: 1·31–0·28 = 1·03· 1·03/1·31 = 79% coverage (95%UI not calculated in the report) |
|  | ATLAS -Denmark | nationally representative | ATLAS | unclear | Psychosis | 2017 and 2020 | 5771877 | unclear | service coverage for psychosis 2017 %= 72·88 |
|  | Santomauro 2024 - Denmark | nationally representative | modelling study | None specified | major depressive disorder | 2000 and 2021 | 174600 (95%UI 2000) 200700 (95%UI 2021) | >= 18 y | Proportion of persons with major depressive disorder receiving minimally adequate treatment by sex and location for 2000 and 2021 (95%UI 95% UIs): 24·8 (95%UI 17·8–35·2) and 26·0 (95%UI 19·1–36·5) |
| UK- England | Improving Access to Psychological Therapies (95%UI IAPT) programme | nationally representative | large-scale initiative that aims to significantly increase the availability of psychological treatments for depression and anxiety disorders within NHS-commissioned services | None specified | Common mental disorders (95%UI CMDs) | 2008-2012 | unclear | unclear | 64·5% coverage (95%UI any treatment) |
|  | Santomauro 2024 - England | nationally representative | modelling study | None specified | major depressive disorder | 2000 and 2021 | 1868800 (95%UI 2000) 2619100 (95%UI 2021) | >= 18 y | Proportion of persons with major depressive disorder receiving minimally adequate treatment by sex and location for 2000 and 2021 (95%UI 95% UIs): 24·5 (95%UI 20·8–29·4) and 25·8 (95%UI 21·7–31·2) |
| Estonia | Santomauro 2024 - Estonia | nationally representative | modelling study | None specified | major depressive disorder | 2000 and 2021 | 63300 (95%UI 2000) 59500 (95%UI 2021) | >= 18 y | Proportion of persons with major depressive disorder receiving minimally adequate treatment by sex and location for 2000 and 2021 (95%UI 95% UIs): 12·0 (95%UI 8·7–16·8) and 13·7 (95%UI 10·0–19·0) |
| European (95%UI Italy, Germany, Hungary, Latvia, Poland and Spain) | Probst 2015 | regionally representative samples | epidemiological study | None specified | individuals with Alcohol use disorders | January 2013 - January 2014 | 1008 | The majority of the 9,098 patients (95%UI 3,715 males and 5,383 females) interviewed came from Hungary (95%UI N = 2,306), followed by Spain (95%UI N = 1,994)· The remaining patients came from Germany (95%UI N = 1,356), Latvia (95%UI N = 1,302), Poland (95%UI N = 1,197) and Italy (95%UI N = 943)· The mean age was 44·3 years (95%UI standard deviation 13·3 years), ranging between 18 and 64 years· On average the patients received 12·7 years of education (95%UI standard deviation 3·6 years) and 13·3 % of the sample was unemployed· | 1,008 patients diagnosed with an alcohol use disorder (95%UI via general practitioner or patient interview) in the past 12 months, the majority (95%UI N = 810) did not receive treatment (95%UI =80·2%, 95%CI 77·6-82·8) |
| Finland | ATLAS -Finland | nationally representative | ATLAS | unclear | Psychosis | 2017 and 2020 | 5532159 | unclear | service coverage for psychosis 2017 and 2020 % = 73,63 ans 97·43 |
|  | Santomauro 2024 - Finland | nationally representative | modelling study | None specified | major depressive disorder | 2000 and 2021 | 223900 (95%UI 2000) 234700 (95%UI 2021) | >= 18 y | Proportion of persons with major depressive disorder receiving minimally adequate treatment by sex and location for 2000 and 2021 (95%UI 95% UIs): 17·9 (95%UI 14·8–22·2) and 20·6 (95%UI 15·2–29·0) |
|  | Hamalainen 2008 | mainland | multidisciplinary epidemiological study | regionally stratified according to the five university hospital regions, each serving approximately one million inhabitan | major depressive episodes and disorder,dysthymia, general anxiety disorder, panic disorderwith or without agoraphobia, agoraphobia, socialphobia, alcohol abuse, and alcohol dependence | August 2000- March 2001 | panic disorder 114 (95%UI 40%), generalized anxiety disorder 75 (95%UI 27%), social anxiety disorder 60 (95%UI 21%), and agoraphobia 33 (95%UI 12%)· Of persons suffering from any anxiety disorder, 71 (95%UI 29%) suffered from mild, 106 (95%UI 44%) from moderate, and 65 (95%UI 27%) from severe disorder· | 30 years; males (95%UI 39%) and 147 females (95%UI 61%)· | The proportion of subjects classified as having MDD who used any health services for mental problems during the past 12 months was 30% for males and 36% for females· The corresponding proportions for anxiety disorder were 38% and 35%· MDD n=64 (95%UI 28%), MDD and anxiety n=38 (95%UI 58%), anxiety and DO n=49 (95%UI 28%)· Fig·1 ·For specific anxiety disorders, the use ofservices differed slightly; for panic disorder, the use was33% (95%UI 38/114), for social phobia 51% (95%UI 30/59), foragoraphobia 39% (95%UI 13/33), and for generalized anxietydisorder 43% (95%UI 32/75)· |
| France | Alonso 2018 - France | nationally representative | cross sectional - WHO World Mental Health Survey Initiative | None specified | anxiety (95%UI agoraphobia, generalized anxiety disorder, panic disorder, PTSD, social phobia, specific phobia, adult separation anxiety disorder)· | 2001-2002 | 2894 | 18-97 y | Any treatment among those with disorder % (95%UI se); possibly adequate treatment % (95%UI se)= 29·4 (95%UI 4·3) ; 13·7 (95%UI 2·9) |
|  | ATLAS -France | nationally representative | ATLAS | unclear | Psychosis | 2017 and 2020 | 65129731 | unclear | service coverage for psychosis 2017 and 2020 % = 63·16 and 81·59 |
|  | Dauriac-Le Masson 2020 | Greater Paris area | Population-based study on mental healthcare utilization | French-speaking homeless people with diagnosed psychiatric disorders | Psychotic disorders, mood disorders, and anxiety disorders | 2009 | 840 homeless· 377 homeless individuals with psychiatric disorders | table 2 | Among 840 homeless people with useable data, 377 (95%UI N = 9762) had a psychiatric disorder· The use of whole-life care for these people may seem high, estimated at 68·7%, but few people were followed up for their disorders (95%UI 18·2%); individuals with a psychotic disorder were more frequently followed up (95%UI 36·5%) than others were (95%UI p < 0·05)· Among those followed up (95%UI n = 86, N = 1760), 63·0% were taking medication· Access to care for these people seemed preserved, but the maintenance of care seemed problematic; indeed, among people with a lifelong whole use of care (95%UI n= 232, N = 6705), 72·3% could be considered to have discontinued care· |
|  | de Vries 2021- France | nationally representative | cross-sectional: World Mental Health Surveys report | None specified | specific phobia | 2001-2002 | all participants (95%UI not only diagnosed)=1436 | 18-97 y | Respondents who obtained treatment for specific phobia %(95%UI se): 9·7 (95%UI 3·2) |
|  | Degenhard 2017 - France | nationally representative | cross sectional - WHO World Mental Health Survey Initiative | None specified | Substance use disorders | 2001-2002 | 2894 | 18-97 y | Minimally adequate treatment among allthose with substanceuse disorders % (95%UI se) = 14·9 (95%UI 3·8) |
|  | Fayyad 2017 - France | nationally representative | cross sectional - WHO World Mental Health Survey Initiative | None specified | ADHD | 2001-2002 | 2894 | 18-97 y | Twelve-month treatment (95%UI any mental health treatment) among respondents with multiply imputed DSM-IV/CIDI adult attention-deficit hyperactivity disorder % (95%UI se)= 0·0 (95%UI 0·0) |
|  | Font 2018 | 47 different sites | Cross-sectional survey | general population | Mood disorders, anxiety disorders, AUDs, SUDs, psychotic disorders | 1999–2003 | 39,617 respondents | age mean (95%UI sd)= 44·7 (95%UI 18·2); female= 54%; | 53·5% lifetime utilization among diagnosed individuals· 46·5% No lifetime utilization of mental health treatment; 35·6% for MDs, 39·7% for PDs, 42·8% for ADs, 56·0% for AUDs, and 56·7% for SUDs)· |
|  | Kadzin 2023- France | nationally representative | Cross-sectional: World Mental Health Surveys report | None specified | major depressive disorder, anxiety disorders | 2001-2002 | 2894 | 18-97 y | Prevalence of ADM use by MDD and anxiety disorder histories (95%UI 12-month MDD), % (95%UI SE)= 30·8 (95%UI 4·7) and 14·6 (95%UI 2·2) |
|  | Santomauro 2024 - France | nationally representative | modelling study | None specified | major depressive disorder | 2000 and 2021 | 2253500 (95%UI 2000) 2655000 (95%UI 2021) | >= 18 y | Proportion of persons with major depressive disorder receiving minimally adequate treatment by sex and location for 2000 and 2021 (95%UI 95% UIs): 26·2 (95%UI 19·0–37·0) and 29·0 (95%UI 21·3–40·7) |
|  | Stein 2021- France | nationally representative | cross sectional - WHO World Mental Health Survey Initiative | None specified | GAD (95%UI generalized anxiety disorder) | 2001-2002 | all participants (95%UI not only diagnosed)=1436 | 18-97 y | Proportion of Respondents with lifetime GAD, who obtained treatment % (95%UI se)= 33·2 (95%UI 4·4) |
|  | Stein 2023 -France | nationally representative | Cross-sectional: World Mental Health Surveys report | None specified | PTSD | 2001-2002 | 2894 | 18-97 y | missing |
| Georgia | Murphy 2018 | Conflict-affected regions, Mtskheta-Mtianeti | Rapid appraisal study on barriers to mental health care utilization | Internally displaced persons (95%UI IDPs) | PTSD, depression, anxiety disorders | 2013 | 29 | unclear | The prevalence of anxiety among IDPs in Georgia is estimated at 13·0% (95%UI 1990s IDPs) and 9·2% (95%UI 2008 IDPs)) [19] and the proportion of those selfreporting anxiety and not using care is high (95%UI 21·6%) |
|  | Santomauro 2024 - Georgia | nationally representative | modelling study | None specified | major depressive disorder | 2000 and 2021 | 127100 (95%UI 2000) 134500 (95%UI 2021) | >= 18 y | Proportion of persons with major depressive disorder receiving minimally adequate treatment by sex and location for 2000 and 2021 (95%UI 95% UIs): 11·0 (95%UI 8·0–15·3) and 11·4 (95%UI 8·3–16·0) |
| Germany | Alonso 2018 - Germany | nationally representative | cross sectional - WHO World Mental Health Survey Initiative | None specified | anxiety (95%UI agoraphobia, generalized anxiety disorder, panic disorder, PTSD, social phobia, specific phobia, adult separation anxiety disorder)· | 2002-2003 | 3555 | 18-97 y | Any treatment among those with disorder % (95%UI se); possibly adequate treatment % (95%UI se)= 24·0 (95%UI 3·7); 13·0 (95%UI 2·8) |
|  | de Vries 2021- Germany | nationally representative | cross-sectional: World Mental Health Surveys report | None specified | specific phobia | 2002-2003 | all participants (95%UI not only diagnosed)= 1323 | 18-95 y | Respondents who obtained treatment for specific phobia %(95%UI se): 20·5 (95%UI 3·6) |
|  | Degenhard 2017 - Germany | nationally representative | cross sectional - WHO World Mental Health Survey Initiative | None specified | Substance use disorders | 2002-2003 | 3555 | 19-95 y | Minimally adequate treatment among allthose with substanceuse disorders % (95%UI se) = 8·2 (95%UI 3·0) |
|  | Fayyad 2017 - Germany | nationally representative | cross sectional - WHO World Mental Health Survey Initiative | None specified | ADHD | 2002-2003 | 3555 | 19-95 y | Twelve-month treatment (95%UI any mental health treatment) among respondents with multiply imputed DSM-IV/CIDI adult attention-deficit hyperactivity disorder % (95%UI se)= 0·0 (95%UI 0·0) |
|  | Kadzin 2023- Germany | nationally representative | Cross-sectional: World Mental Health Surveys report | None specified | major depressive disorder, anxiety disorders | 2002-2003 | 3555 | 19-95 y | Prevalence of ADM use by MDD and anxiety disorder histories (95%UI 12-month MDD), % (95%UI SE)= 33·9 (95%UI 5·1) and 14·8 (95%UI 3·5) |
|  | Reich 2023 | Dresden city | Cross-sectional | None specified | any | November 2015 - December 2016 | 1180 | mean age ±SD =17·9±2·3 years, female 8·3% | Of n = 597 participants with any lifetime mental disorder, n = 193 (95%UI 32·4% [28·4; 36·7]) had ever used health services because of a mental health, psychosomatic, or substance use problem· 65 out of 303 with one diagnosis ever used services· 55/152 with 2 diagnoses· 73/142 with 3 diagnoses· (95%UI Of n=597 participants with any lifetime mental disorder, n = 72 participants (95%UI 12·1% [9·5; 15·2]) reported to have received CBT and n=66 (95%UI 10·7% [8·4, 13·7]) to have been treated with other psychotherapy· Only n=30 participants (95%UI 5·4% [3·7; 7·8]) with any lifetime mental disorder reported pharmacological treatment· Of those with any lifetime mental disorder, 12·3% [9·8; 15·3] had been thinking about using health services because of mental health, psychosomatic, or substance use problems without ever doing it)· |
|  | Santomauro 2024 - Germany | nationally representative | modelling study | None specified | major depressive disorder | 2000 and 2021 | 2179000 (95%UI 2000) 2992500 (95%UI 2021) | >= 18 y | Proportion of persons with major depressive disorder receiving minimally adequate treatment by sex and location for 2000 and 2021 (95%UI 95% UIs): 32·9 (95%UI 24·7–43·7) and 35·0 (95%UI 26·0–48·0) |
|  | Stein 2021- Germany | nationally representative | cross sectional - WHO World Mental Health Survey Initiative | None specified | GAD (95%UI generalized anxiety disorder) | 2002-2003 | all participants (95%UI not only diagnosed)= 1323 | 18-95 y | Proportion of Respondents with lifetime GAD, who obtained treatment % (95%UI se)= 34·2 (95%UI 8·7) |
|  | Stein 2023 -Germany | nationally representative | Cross-sectional: World Mental Health Surveys report | None specified | PTSD | 2002-2003 | 3555 | 19-95 y | missing |
|  | Stolzenburg 2019 | Nationally representative | cross-sectional | None specified | any mental disorder (95%UI F1, F3, F4, F5) ICD-10 | unclear | 207 participants | mean age 49·6 years old (95%UI S·D· = 16·3) and mostly female· As seen in Table 1 the sample included people of all ages, with lower numbers in the 35–44 years age group | 50 persons (95%UI 24·2% of the sample) reported that they had sought help with a psychiatrist and 86 persons (95%UI 41·5%) with a psychotherapist/psychologist· Details in notes for each diagnosis· |
| Greece | Santomauro 2024 - Greece | nationally representative | modelling study | None specified | major depressive disorder | 2000 and 2021 | 689100 (95%UI 2000) 605300 (95%UI 2021) | >= 18 y | Proportion of persons with major depressive disorder receiving minimally adequate treatment by sex and location for 2000 and 2021 (95%UI 95% UIs): 25·6 (95%UI 18·8–36·5) and 25·6 (95%UI 18·8–35·8) |
| Hungary | Santomauro 2024 - Hungary | nationally representative | modelling study | None specified | major depressive disorder | 2000 and 2021 | 267900 (95%UI 2000) 39000 (95%UI 2021) | >= 18 y | Proportion of persons with major depressive disorder receiving minimally adequate treatment by sex and location for 2000 and 2021 (95%UI 95% UIs): 12·5 (95%UI 9·2–17·7) and 13·4 (95%UI 9·8–18·7) |
| Iceland | Santomauro 2024 - Iceland | nationally representative | modelling study | None specified | major depressive disorder | 2000 and 2021 | 6300 (95%UI 2000) 8500 (95%UI 2021) | >= 18 y | Proportion of persons with major depressive disorder receiving minimally adequate treatment by sex and location for 2000 and 2021 (95%UI 95% UIs): 25·9 (95%UI 18·2–37·1) and 26·6 (95%UI 19·3–37·1) |
| Ireland | Santomauro 2024 - Ireland | nationally representative | modelling study | None specified | major depressive disorder | 2000 and 2021 | 134300 (95%UI 2000) 217400 (95%UI 2021) | >= 18 y | Proportion of persons with major depressive disorder receiving minimally adequate treatment by sex and location for 2000 and 2021 (95%UI 95% UIs): 26·7 (95%UI 19·0–38·7) and 30·1 (95%UI 21·3–42·9) |
| Israel | Alonso 2018 - Israel | nationally representative | cross sectional - WHO World Mental Health Survey Initiative | None specified | anxiety (95%UI agoraphobia, generalized anxiety disorder, panic disorder, PTSD, social phobia, specific phobia, adult separation anxiety disorder)· | 2003–2004 | 4859 | 21-98 y | Any treatment among those with disorder % (95%UI se); possibly adequate treatment % (95%UI se)= 41·2 (95%UI 3·9); 15·8 (95%UI 3·0) |
|  | Degenhard 2017 - Israel | nationally representative | cross sectional - WHO World Mental Health Survey Initiative | None specified | Substance use disorders | 2003-2004 | 4859 | 21-98 y | Minimally adequate treatment among allthose with substanceuse disorders % (95%UI se) = 3·4 (95%UI 1·4) |
|  | Kadzin 2023- Israel | nationally representative | Cross-sectional: World Mental Health Surveys report | None specified | major depressive disorder, anxiety disorders | 2003-2004 | 4859 | 21-98 y | Prevalence of ADM use by MDD and anxiety disorder histories (95%UI 12-month MDD), % (95%UI SE)= 13·6 (95%UI 2·1) and 14·1 (95%UI 2·6) |
|  | Santomauro 2024 - Israel | nationally representative | modelling study | None specified | major depressive disorder | 2000 and 2021 | 242600 (95%UI 2000) 369700 (95%UI 2021) | >= 18 y | Proportion of persons with major depressive disorder receiving minimally adequate treatment by sex and location for 2000 and 2021 (95%UI 95% UIs): 18·0 (95%UI 12·9–25·3) and 20·0 (95%UI 14·6–28·3) |
|  | Stein 2020 - Israel | nationally representative | Cross-sectional: World Mental Health Surveys report | None specified | PTSD | 2003-2004 | 8463 | 21-98 y | among respondents with lifetime PTSD, % (95%UI SE) obtained treatment = 12·0 (95%UI 4·1) |
|  | Stein 2021- Israel | nationally representative | cross sectional - WHO World Mental Health Survey Initiative | None specified | GAD (95%UI generalized anxiety disorder) | 2003-2004 | all participants (95%UI not only diagnosed) 4859 | 21-98 y | Proportion of Respondents with lifetime GAD, who obtained treatment % (95%UI se)= 36·9 (95%UI 3·4) |
| Italy | Alonso 2018 - Italy | nationally representative | cross sectional - WHO World Mental Health Survey Initiative | None specified | anxiety (95%UI agoraphobia, generalized anxiety disorder, panic disorder, PTSD, social phobia, specific phobia, adult separation anxiety disorder)· | 2001-2002 | 4712 | 21-98 | Any treatment among those with disorder % (95%UI se); possibly adequate treatment % (95%UI se)= 29·7 (95%UI 3·3); 9·1 (95%UI 2·3) |
|  | ATLAS -Italy | nationally representative | ATLAS | unclear | Psychosis | 2017 and 2020 | 60550092 | unclear | service coverage for psychosis 2017 and 2020 % = 81,51 and 58·85 |
|  | Barbato 2016 | Lombardy | Survey-based research on access to psychosocial treatments (95%UI service utilization were retrieved from the psychiatric database of Lombardy, Italy, covering a population of 9,743,000, for all adults who had at least one contact in 2009 with psychiatric services) | Patients within the public mental health system, in the psychiatric database of Lombardy | Bipolar disorder, with comparisons to schizophrenia and unipolar depression | 2009 | 9,743,000 (95%UI population considered); 8,899 subjects with bipolar disorder | Mean age (95%UI SD): bipolar: 52·5 (95%UI 14·6); schizophrenia: 49·4 (95%UI 14·6); unipolar depression: 54·2 (95%UI 15·6)· Female: biplar: 4,754 (95%UI 57·4); schizophrenia: 14,713 (95%UI 47·6); unipolar: 19,011 (95%UI 66·9) | Table 2 shows that more than 80% of bipolar disorders were treated on outpatient basis, as indicated by the high frequency of clinical (95%UI 45·4%) and community (95%UI 35·6) packages· A few had one or more hospital admissions and even fewer were treated in residential or day care settings· Compared to the bipolar group, patients with schizophrenia received more community (95%UI 48·6%), day care (95%UI 6·5%) and residential (95%UI 7·8%) packages and fewer clinical (95%UI 27·1%) and hospital (95%UI 9·9%) packages· |
|  | Corrao 2021 | resident in Lombardy, Emilia-Romagna, Lazio, and Palermo | cohort study | general population | any | January 2013 to December 2016 for Lombardy, from January 2015 to December 2016 for Emilia-Romagna and Palermo, and from January 2015 to December 2015 for Lazio | 45,761 individuals met the inclusion newly taken-intocare patients with diagnosis of depression (95%UI 73%), personality disorder (95%UI 12%), schizophrenia (95%UI 10%), or bipolar disorder (95%UI 4%)· | 18–65 y | Between 71 (95%UI personality disorder) and 83% (95%UI bipolar disorder) of these patients received generic MHC· There was again a general fall in coverage rates, with only 10% of patients with depression, 15% with personality disorder, and 20% with bipolar disorder still in contact with services 5 years after diagnosis· Patients with schizophrenia had longer contacts, with a coverage rate around 50%· |
|  | de Vries 2021- Italy | nationally representative | cross-sectional: World Mental Health Surveys report | None specified | specific phobia | 2001-2002 | all participants (95%UI not only diagnosed)= 1779 | 18-100 y | Respondents who obtained treatment for specific phobia %(95%UI se): 13·5 (95%UI 2·9) |
|  | Degenhard 2017 - Italy | nationally representative | cross sectional - WHO World Mental Health Survey Initiative | None specified | Substance use disorders | 2001-2002 | 4712 | 18-100 y | Minimally adequate treatment among allthose with substanceuse disorders % (95%UI se) = 4·1 (95%UI 0·6) |
|  | Fayyad 2017 - Italy | nationally representative | cross sectional - WHO World Mental Health Survey Initiative | None specified | ADHD | 2001-2002 | 4712 | 18-100 y | Twelve-month treatment (95%UI any mental health treatment) among respondents with multiply imputed DSM-IV/CIDI adult attention-deficit hyperactivity disorder % (95%UI se)= 0·0 (95%UI 0·0) |
|  | Kadzin 2023- Italy | nationally representative | Cross-sectional: World Mental Health Surveys report | None specified | major depressive disorder, anxiety disorders | 2001-2002 | 4712 | 18-100 y | Prevalence of ADM use by MDD and anxiety disorder histories (95%UI 12-month MDD), % (95%UI SE)= 17·2 (95%UI 3·2) and 11·2 (95%UI 2·5) |
|  | Lora 2012 | Lombardy region | System-level evaluation of mental health service accessibility and patterns of care | Individuals with various mental health diagnoses (95%UI schizophrenia, neurotic, affective, personality disorders) | Schizophrenia, affective disorders, neurotic disorders, personality disorders | 1999–2009 | 146 per 10,000 adult population accessed services in 2009 | table 1 | data on treated incidence and treated prevalence are reported per year |
|  | Santomauro 2024 - Italy | nationally representative | modelling study | None specified | major depressive disorder | 2000 and 2021 | 2058500 (95%UI 2000) 2406000 (95%UI 2021) | >= 18 y | Proportion of persons with major depressive disorder receiving minimally adequate treatment by sex and location for 2000 and 2021 (95%UI 95% UIs): 19·9 (95%UI 17·0–23·9) and 19·5 (95%UI 16·6–23·4) |
|  | Stein 2021- Italy | nationally representative | cross sectional - WHO World Mental Health Survey Initiative | None specified | GAD (95%UI generalized anxiety disorder) | 2001-2002 | all participants (95%UI not only diagnosed)= 1779 | 18-100 y | Proportion of Respondents with lifetime GAD, who obtained treatment % (95%UI se)= 24·3 (95%UI 4·8) |
|  | Stein 2023 -Italy | nationally representative | Cross-sectional: World Mental Health Surveys report | None specified | PTSD | 2001-2002 | 4712 | 18-100 y | missing |
| Kazakhstan | Santomauro 2024 - Kazakhstan | nationally representative | modelling study | None specified | major depressive disorder | 2000 and 2021 | 373600 (95%UI 2000) 542900 (95%UI 2021) | >= 18 y | Proportion of persons with major depressive disorder receiving minimally adequate treatment by sex and location for 2000 and 2021 (95%UI 95% UIs): 9·7 (95%UI 7·0–13·5) and 11·4 (95%UI 8·2–15·9) |
| Kyrgyzstan | Santomauro 2024 - Kyrgyzstan | nationally representative | modelling study | None specified | major depressive disorder | 2000 and 2021 | 111100 (95%UI 2000) 188200 (95%UI 2021) | >= 18 y | Proportion of persons with major depressive disorder receiving minimally adequate treatment by sex and location for 2000 and 2021 (95%UI 95% UIs): 9·4 (95%UI 6·7–13·1) and 10·6 (95%UI 7·5–15·0) |
| Latvia | Santomauro 2024 - Latvia | nationally representative | modelling study | None specified | major depressive disorder | 2000 and 2021 | 94400 (95%UI 2000) 90200 (95%UI 2021) | >= 18 y | Proportion of persons with major depressive disorder receiving minimally adequate treatment by sex and location for 2000 and 2021 (95%UI 95% UIs): 11·8 (95%UI 8·6–16·6) and 12·9 (95%UI 9·4–17·8) |
| Lithuania | Santomauro 2024 - Lithuania | nationally representative | modelling study | None specified | major depressive disorder | 2000 and 2021 | 143200 (95%UI 2000) 146200 (95%UI 2021) | >= 18 y | Proportion of persons with major depressive disorder receiving minimally adequate treatment by sex and location for 2000 and 2021 (95%UI 95% UIs): 12·2 (95%UI 8·9–17·2) and 12·9 (95%UI 9·5–18·2) |
| Luxembourg | Santomauro 2024 - Luxembourg | nationally representative | modelling study | None specified | major depressive disorder | 2000 and 2021 | 13000 (95%UI 2000) 19800 (95%UI 2021) | >= 18 y | Proportion of persons with major depressive disorder receiving minimally adequate treatment by sex and location for 2000 and 2021 (95%UI 95% UIs): 25·6 (95%UI 18·3–37·0) and 26·9 (95%UI 19·3–38·2) |
| Malta | Santomauro 2024 - Malta | nationally representative | modelling study | None specified | major depressive disorder | 2000 and 2021 | 9200 (95%UI 2000) 12400 (95%UI 2021) | >= 18 y | Proportion of persons with major depressive disorder receiving minimally adequate treatment by sex and location for 2000 and 2021 (95%UI 95% UIs): 23·6 (95%UI 17·2–34·0) and 26·3 (95%UI 19·6–36·4) |
| Monaco | ATLAS -Monaco | nationally representative | ATLAS | unclear | Psychosis | 2017 and 2020 | 38967 | unclear | service coverage for psychosis 2017 and 2020 % = 96,84 and 83·68 |
|  | Santomauro 2024 - Monaco | nationally representative | modelling study | None specified | major depressive disorder | 2000 and 2021 | 1200 (95%UI 2000) 1700 (95%UI 2021) | >= 18 y | Proportion of persons with major depressive disorder receiving minimally adequate treatment by sex and location for 2000 and 2021 (95%UI 95% UIs): 26·0 (95%UI 19·3–36·8) and 26·2 (95%UI 19·4–36·7) |
| Montenegro | Santomauro 2024 - Montenegro | nationally representative | modelling study | None specified | major depressive disorder | 2000 and 2021 | 13000 (95%UI 2000) 2400 (95%UI 2021) | >= 18 y | Proportion of persons with major depressive disorder receiving minimally adequate treatment by sex and location for 2000 and 2021 (95%UI 95% UIs): 12·4 (95%UI 9·0–17·1) and 13·5 (95%UI 9·8–18·8) |
| North Macedonia | Santomauro 2024 - North Macedonia | nationally representative | modelling study | None specified | major depressive disorder | 2000 and 2021 | 36000 (95%UI 2000) 56600 (95%UI 2021) | >= 18 y | Proportion of persons with major depressive disorder receiving minimally adequate treatment by sex and location for 2000 and 2021 (95%UI 95% UIs): 11·6 (95%UI 8·5–16·5) and 12·8 (95%UI 9·3–17·8) |
| UK- Northern Ireland | de Vries 2021- Northern Ireland | nationally representative | cross-sectional: World Mental Health Surveys report | None specified | specific phobia | 2005-2008 | all participants (95%UI not only diagnosed)=4340 | 18-97 y | Respondents who obtained treatment for specific phobia %(95%UI se): 14·4 (95%UI 1·5) |
|  | Degenhard 2017 - Northern Ireland | nationally representative | cross sectional - WHO World Mental Health Survey Initiative | None specified | Substance use disorders | 2005-2008 | 4340 | 18-97 y | Minimally adequate treatment among allthose with substanceuse disorders % (95%UI se) = 7·1 (95%UI 2·0) |
|  | Fayyad 2017 - Northern Ireland | nationally representative | cross sectional - WHO World Mental Health Survey Initiative | None specified | ADHD | 2004-2007 | 4340 | 18-97 y | Twelve-month treatment (95%UI any mental health treatment) among respondents with multiply imputed DSM-IV/CIDI adult attention-deficit hyperactivity disorder % (95%UI se)= 0·6 (95%UI 0·6) |
|  | Harris 2024 - Northern Ireland | nationally representative | cross-sectional: World Mental Health Surveys report | None specified | major depressive disorder, bipolar spectrum disorder, panic disorder/agoraphobia, generalized anxiety disorder, posttraumatic stress disorder, social phobia, specific phobia, and substance use disorders (95%UI alcohol and illicit drug abuse with or without dependence) | 2005-2008 | 1986 | 18-97 y | 12-month use of providers for mental health among those with any DSM-IV 12-month disorders: 50·6 (95%UI SE=2·6) |
|  | Santomauro 2024 - Northern Ireland | nationally representative | modelling study | None specified | major depressive disorder | 2000 and 2021 | 65400 (95%UI 2000) 89000 (95%UI 2021) | >= 18 y | Proportion of persons with major depressive disorder receiving minimally adequate treatment by sex and location for 2000 and 2021 (95%UI 95% UIs): 24·5 (95%UI 17·4–34·9) and 26·1 (95%UI 18·7–37·4) |
|  | Stein 2020 - Northern Irland | nationally representative | Cross-sectional: World Mental Health Surveys report | None specified | PTSD | 2005-2008 | 4340 | 18-97 | among respondents with lifetime PTSD, % (95%UI SE) obtained treatment = 39·2 (95%UI 4·3) |
|  | Stein 2021- Northern Ireland | nationally representative | cross sectional - WHO World Mental Health Survey Initiative | None specified | GAD (95%UI generalized anxiety disorder) | 2005-2008 | all participants (95%UI not only diagnosed)=4340 | 18-97 y | Proportion of Respondents with lifetime GAD, who obtained treatment % (95%UI se)= 39·6 (95%UI 2·3) |
| Norway | ATLAS -Norway | nationally representative | ATLAS | unclear | Psychosis | 2017 and 2020 | 5378859 | unclear | service coverage for psychosis 2017 % = 48·68 |
|  | Santomauro 2024 - Norway | nationally representative | modelling study | None specified | major depressive disorder | 2000 and 2021 | 127700 (95%UI 2000) 191300 (95%UI 2021) | >= 18 y | Proportion of persons with major depressive disorder receiving minimally adequate treatment by sex and location for 2000 and 2021 (95%UI 95% UIs): 25·5 (95%UI 21·3–31·0) and 26·7 (95%UI 22·5–32·3) |
| Poland | de Vries 2021- Poland | nationally representative | cross-sectional: World Mental Health Surveys report | None specified | specific phobia | 2010-2011 | all participants (95%UI not only diagnosed)= 10081 | 18-65 y | Respondents who obtained treatment for specific phobia %(95%UI se): 13·3 (95%UI 2·2) |
|  | Degenhard 2017 - Poland | nationally representative | cross sectional - WHO World Mental Health Survey Initiative | None specified | Substance use disorders | 2010-2011 | 10081 | 18-65 y | Minimally adequate treatment among allthose with substanceuse disorders % (95%UI se) = 6·2 (95%UI 1·8) |
|  | Fayyad 2017 - Poland | nationally representative | cross sectional - WHO World Mental Health Survey Initiative | None specified | ADHD | 2010-2011 | 10081 | 18-65 y | Twelve-month treatment (95%UI any mental health treatment) among respondents with multiply imputed DSM-IV/CIDI adult attention-deficit hyperactivity disorder % (95%UI se)= 5·8 (95%UI 5·7) |
|  | Harris 2024 - Poland | nationally representative | cross-sectional: World Mental Health Surveys report | None specified | major depressive disorder, bipolar spectrum disorder, panic disorder/agoraphobia, generalized anxiety disorder, posttraumatic stress disorder, social phobia, specific phobia, and substance use disorders (95%UI alcohol and illicit drug abuse with or without dependence) | 2010-2011 | 4000 | 18-65 y | 12-month use of providers for mental health among those with any DSM-IV 12-month disorders: 18·2 % (95%UI SE= 1·7) |
|  | Santomauro 2024 - Poland | nationally representative | modelling study | None specified | major depressive disorder | 2000 and 2021 | 555000 (95%UI 2000) 732000 (95%UI 2021) | >= 18 y | Proportion of persons with major depressive disorder receiving minimally adequate treatment by sex and location for 2000 and 2021 (95%UI 95% UIs): 11·5 (95%UI 9·6–14·1) and 12·6 (95%UI 10·6–15·2) |
|  | Stein 2021- Poland | nationally representative | cross sectional - WHO World Mental Health Survey Initiative | None specified | GAD (95%UI generalized anxiety disorder) | 2010-2011 | all participants (95%UI not only diagnosed)= 10081 | 18-65 y | Proportion of Respondents with lifetime GAD, who obtained treatment % (95%UI se)= 37·8 (95%UI 5·0) |
| Portugal | Alonso 2018 - Portugal | nationally representative | cross sectional - WHO World Mental Health Survey Initiative | None specified | anxiety (95%UI agoraphobia, generalized anxiety disorder, panic disorder, PTSD, social phobia, specific phobia, adult separation anxiety disorder)· | 2008-2009 | 3849 | 18-81 | Any treatment among those with disorder % (95%UI se); possibly adequate treatment % (95%UI se)= 32·0 (95%UI 2·4); 10·9 (95%UI 1·5) |
|  | de Vries 2021- Portugal | nationally representative | cross-sectional: World Mental Health Surveys report | None specified | specific phobia | 2008-2009 | all participants (95%UI not only diagnosed)=3849 | 18-81 y | Respondents who obtained treatment for specific phobia %(95%UI se): 22·1 (95%UI 2·2) |
|  | Degenhard 2017 - Portugal | nationally representative | cross sectional - WHO World Mental Health Survey Initiative | None specified | Substance use disorders | 2008-2009 | 3849 | 18-81 y | Minimally adequate treatment among allthose with substanceuse disorders % (95%UI se) = 10·3 (95%UI 6·2) |
|  | Fayyad 2017 - Portugal | nationally representative | cross sectional - WHO World Mental Health Survey Initiative | None specified | ADHD | 2008-2009 | 2276 | 18-81 y | Twelve-month treatment (95%UI any mental health treatment) among respondents with multiply imputed DSM-IV/CIDI adult attention-deficit hyperactivity disorder % (95%UI se)= 0·0 (95%UI 0·0) |
|  | Grigaite˙2024 | Nationally representative | cross sectional - WHO World Mental Health Survey Initiative | females who survived IPV | ANY | 2008–2010 | 96 females who experienced IPV | table 1 | Table 4 has the detail for each mental disorder· over 46 % of IPV survivors who had any mental health diagnosis received treatment in specialised mental health services, and over 42 % of them were treated by a psychiatrist· As much as 61 % received mental health care from general medical doctors, and over 71 % consulted other healthcare professionals· |
|  | Harris 2024 - Portugal | nationally representative | cross-sectional: World Mental Health Surveys report | None specified | major depressive disorder, bipolar spectrum disorder, panic disorder/agoraphobia, generalized anxiety disorder, posttraumatic stress disorder, social phobia, specific phobia, and substance use disorders (95%UI alcohol and illicit drug abuse with or without dependence) | 2008-2009 | 2060 | 18-81 y | 12-month use of providers for mental health among those with any DSM-IV 12-month disorders: 36·9% (95%UI 2·2) |
|  | Kadzin 2023- Portugal | nationally representative | Cross-sectional: World Mental Health Surveys report | None specified | major depressive disorder, anxiety disorders | 2008-2009 | 3849 | 18-81 y | Prevalence of ADM use by MDD and anxiety disorder histories (95%UI 12-month MDD), % (95%UI SE)= 32·8 (95%UI 3·1) and 15·8 (95%UI 2·0) |
|  | Santomauro 2024 - Portugal | nationally representative | modelling study | None specified | major depressive disorder | 2000 and 2021 | 571400 (95%UI 2000) 583400 (95%UI 2021) | >= 18 y | Proportion of persons with major depressive disorder receiving minimally adequate treatment by sex and location for 2000 and 2021 (95%UI 95% UIs): 19·7 (95%UI 14·4–27·6) and 21·8 (95%UI 16·1–30·5) |
|  | Silva 2020 | Nationally representative survey | National mental health survey on barriers to care, World Mental Health Survey (95%UI WMHS) | None specified | Mood and anxiety disorders, alcohol use | 2009 | 809 | mean age (95%UI sd) = 42·3 (95%UI 16·2); female 597 (95%UI 66·9) | Of the 2060 Part II participants, 809 (95%UI 22·0%) met criteria for a 12-month mental disorder and, among those, 489 (95%UI 65·4%) reported no service use during that period· Single participants had 62% lower odds of having received treatment than married patients (95%UI OR ¼ 0·38; 95% CI: 0·20–0·70; p¼0·002)· Participants with basic or secondary education had 58% lower odds of having received treatment than those with university level (95%UI OR ¼ 0·42; 95% CI: 0·24–0·73; p¼0·002)· |
|  | Stein 2021- Portugal | nationally representative | cross sectional - WHO World Mental Health Survey Initiative | None specified | GAD (95%UI generalized anxiety disorder) | 2008-2009 | all participants (95%UI not only diagnosed)=3849 | 18-81 y | Proportion of Respondents with lifetime GAD, who obtained treatment % (95%UI se)= 41·3 (95%UI 2·7) |
|  | Stein 2023 -Portugal | nationally representative | Cross-sectional: World Mental Health Surveys report | None specified | PTSD | 2008-2009 | 2060 | 18-81 y | among respondents with lifetime PTSD, % (95%UI SE) obtained treatment = 28·7 (95%UI 4·4) |
| Republic of Moldova | Santomauro 2024 - Republic of Moldova | nationally representative | modelling study | None specified | major depressive disorder | 2000 and 2021 | 116500 (95%UI 2000) 130400 (95%UI 2021) | >= 18 y | Proportion of persons with major depressive disorder receiving minimally adequate treatment by sex and location for 2000 and 2021 (95%UI 95% UIs): 11·1 (95%UI 8·1–15·5) and 12·3 (95%UI 8·9–17·2) |
| Romania | Alonso 2018 - Romania | nationally representative | cross sectional - WHO World Mental Health Survey Initiative | None specified | anxiety (95%UI agoraphobia, generalized anxiety disorder, panic disorder, PTSD, social phobia, specific phobia, adult separation anxiety disorder)· | 2005-2006 | 2357 | 18-96 y | Any treatment among those with disorder % (95%UI se); possibly adequate treatment % (95%UI se)= 29·2 (95%UI 4·1); 8·7 (95%UI 2·4) |
|  | de Vries 2021- Romania | nationally representative | cross-sectional: World Mental Health Surveys report | None specified | specific phobia | 2005-2006 | all participants (95%UI not only diagnosed)=2357 | 18-96 y | Respondents who obtained treatment for specific phobia %(95%UI se): 15·4 (95%UI 3·3) |
|  | Degenhard 2017- Romania | nationally representative | cross sectional - WHO World Mental Health Survey Initiative | None specified | Substance use disorders | 2005-2006 | 2357 | 18-65 y | Minimally adequate treatment among allthose with substanceuse disorders % (95%UI se) = 10·2 (95%UI 8·0) |
|  | Fayyad 2017 - Romania | nationally representative | cross sectional - WHO World Mental Health Survey Initiative | None specified | ADHD | 2005-2006 | 2357 | 18-96 y | Twelve-month treatment (95%UI any mental health treatment) among respondents with multiply imputed DSM-IV/CIDI adult attention-deficit hyperactivity disorder % (95%UI se)= 0·0 (95%UI 0·0) |
|  | Harris 2024 - Romania | nationally representative | cross-sectional: World Mental Health Surveys report | None specified | major depressive disorder, bipolar spectrum disorder, panic disorder/agoraphobia, generalized anxiety disorder, posttraumatic stress disorder, social phobia, specific phobia, and substance use disorders (95%UI alcohol and illicit drug abuse with or without dependence) | 2005-2006 | 2357 | 18-96 y | 12-month use of providers for mental health among those with any DSM-IV 12-month disorders: 20·0% (95%UI SE=2·6) |
|  | Kadzin 2023- Romania | nationally representative | Cross-sectional: World Mental Health Surveys report | None specified | major depressive disorder, anxiety disorders | 2005-2006 | 2357 | 18-96 y | Prevalence of ADM use by MDD and anxiety disorder histories (95%UI 12-month MDD), % (95%UI SE)= 11· 1 (95%UI 5·5) and 10·0 (95%UI 4·5) |
|  | Santomauro 2024 - Romania | nationally representative | modelling study | None specified | major depressive disorder | 2000 and 2021 | 440300 (95%UI 2000) 512700 (95%UI 2021) | >= 18 y | Proportion of persons with major depressive disorder receiving minimally adequate treatment by sex and location for 2000 and 2021 (95%UI 95% UIs): 15·4 (95%UI 11·4–21·5) and 17·0 (95%UI 12·4–24·0) |
|  | Stein 2023 -Romania | nationally representative | Cross-sectional: World Mental Health Surveys report | None specified | PTSD | 2005–2006 | 2357 | 18-96 y | among respondents with lifetime PTSD, % (95%UI SE) obtained treatment = 6·9 (95%UI 5·2) |
| Russian Federation | Cook 2020 | Arkhangelsk and Novosibirsk | Cross-sectional population-based study | None specified | Depression and anxiety | 2015–2018 | moderate depression 334, moderate anxiety 181, comorbid 121 | The age-standardised prevalence of moderate depression (95%UI PHQ-9 ≥ 10) for those aged 35–69 in Novosibirsk was 10·6% (95%UI 95% CI 9·4, 11·9%) and in Arkhangelsk 6·3% (95%UI 95% CI 5·4, 7·3%)· The age-standardised prevalence of moderate anxiety (95%UI GAD-7 ≥ 10) was 6·0% (95%UI 95% CI 5·1, 7·0%) in Novosibirsk and 3·8% (95%UI 95% CI 3·0, 4·6%) in Arkhangelsk | Among those with PHQ-9 ≥ 10 17% reported ever having been diagnosed with depression (95%UI equivalent finding for anxiety 29%)· Only 1·5% of those with PHQ-9 ≥ 10 reported using anti-depressants and 0·6% of those with GAD- 7 ≥ 10 reported using anxiolytics· No males with PHQ-9≥ 10 and/or GAD-7 ≥ 10 reported use of anti-depressants or anxiolytics· Use of health services increased with increasing severity of both depression and anxiety |
|  | Santomauro 2024 - Russian Federation | nationally representative | modelling study | None specified | major depressive disorder | 2000 and 2021 | 4086800 (95%UI 2000) 4771300 (95%UI 2021) | >= 18 y | Proportion of persons with major depressive disorder receiving minimally adequate treatment by sex and location for 2000 and 2021 (95%UI 95% UIs): 10·9 (95%UI 9·1–13·2) and 12·8 (95%UI 10·8–15·6) |
| San Marino | Santomauro 2024 - San Marino | nationally representative | modelling study | None specified | major depressive disorder | 2000 and 2021 | 1000 (95%UI 2000) 1500 (95%UI 2021) | >= 18 y | Proportion of persons with major depressive disorder receiving minimally adequate treatment by sex and location for 2000 and 2021 (95%UI 95% UIs): 27·5 (95%UI 20·1–39·7) and 27·3 (95%UI 20·0–38·7) |
| UK- Scotland | Santomauro 2024 - UK- Scotland | nationally representative | modelling study | None specified | major depressive disorder | 2000 and 2021 | 176900 (95%UI 2000) 229400 (95%UI 2021) | >= 18 y | Proportion of persons with major depressive disorder receiving minimally adequate treatment by sex and location for 2000 and 2021 (95%UI 95% UIs): 23·7 (95%UI 17·3–34·1) and 25·7 (95%UI 18·6–36·8) |
| Serbia | Santomauro 2024 - Serbia | nationally representative | modelling study | None specified | major depressive disorder | 2000 and 2021 | 229200 (95%UI 2000) 256500 (95%UI 2021) | >= 18 y | Proportion of persons with major depressive disorder receiving minimally adequate treatment by sex and location for 2000 and 2021 (95%UI 95% UIs): 11·7 (95%UI 8·6–16·4) and 12·8 (95%UI 9·4–17·7) |
| Slovak Republic | Brazinova 2019 | Nationally representative survey | Analysis of mental health care gaps and unmet needs from National Health Information Center (NHIC), 2. the Statistical Office and 3. the Social Insurance Agency. | None specified | Mood disorders, anxiety disorders, alcohol dependence, and schizophrenia | 2015 | Not specified | General adult population aged 15–64 with mental disorders | Treatment gap estimated proportion of persons in need of treatment in the Slovak Republic who do not receive it: affective disorder= 67 (95%UI range 0-73·6); anxiety = 85 (95%UI 76·3–92·7); alcohol dependence= 80 (95%UI 0–88·1); schizophrenia 0 (95%UI 0–44·4) |
| Slovakia | Santomauro 2024 - Slovakia | nationally representative | modelling study | None specified | major depressive disorder | 2000 and 2021 | 118000 (95%UI 2000) 153100 (95%UI 2021) | >= 18 y | Proportion of persons with major depressive disorder receiving minimally adequate treatment by sex and location for 2000 and 2021 (95%UI 95% UIs): 12·1 (95%UI 8·9–17·1) and 13·4 (95%UI 9·9–19·0) |
| Slovenia | ATLAS -Slovenia | nationally representative | ATLAS | unclear | Psychosis | 2017 and 2020 | 2078654 | unclear | service coverage for psychosis 2017 and 2020 % =71·81 and 64·98 |
|  | Santomauro 2024 - Slovenia | nationally representative | modelling study | None specified | major depressive disorder | 2000 and 2021 | 65700 (95%UI 2000) 70800 (95%UI 2021) | >= 18 y | Proportion of persons with major depressive disorder receiving minimally adequate treatment by sex and location for 2000 and 2021 (95%UI 95% UIs): 13·2 (95%UI 9·8–18·6) and 15·0 (95%UI 10·9–20·7) |
| Spain | Ballester 2020 | nationally representative | UNIVERSAL: Web-based surveys | Spanish first-year university students | major depressive episode(95%UI MDE), mania/hypomania, generalized anxiety disorder(95%UI GAD), panic disorder(95%UI PD), alcohol abuse/dependence(95%UI AUD), drug abuse/dependence(95%UI DUD), and adult attention-deficit/hyperactivity disorder(95%UI ADHD) | October 2014 -October 2015 | 2118 | mean age=18·8 y; 55·4% female | In this study, 12·6% of students with possible mental disorder and 2·3% of those without mental disorder reported receiving treatment in the past year, respectively· The receipt of mental health treatment was highest among participants with 12-month anxiety disorders: 35·3% (95%UI se=7·6) for PD and 20·1% (95%UI 7·6) for GAD; and increased with increasing comorbidity up to 18·0-19·0% when students reported two or more disorders· MDD 16% (95%UI SE=1·83); Broad mania (95%UI 16·1%, se=4·69); alcohol abuse 7·6 % (95%UI se=2·35), ADHD 10·3% (95%UI se=1·97)· |
|  | de Vries 2021- Spain | nationally representative | cross-sectional: World Mental Health Surveys report | None specified | specific phobia | 2001-2002 | all participants (95%UI not only diagnosed)= 2121 | 18-96 y | Respondents who obtained treatment for specific phobia %(95%UI se): 7·6 (95%UI 2·3) |
|  | Degenhard 2017 - Spain | nationally representative | cross sectional - WHO World Mental Health Survey Initiative | None specified | Substance use disorders | 2001-2002 | 5473 | 18-98 y | Minimally adequate treatment among allthose with substanceuse disorders % (95%UI se) = 5·2 (95%UI 1·2) |
|  | Fayyad 2017 - Spain | nationally representative | cross sectional - WHO World Mental Health Survey Initiative | None specified | ADHD | 2001-2002 | 5473 | 18-98 y | Twelve-month treatment (95%UI any mental health treatment) among respondents with multiply imputed DSM-IV/CIDI adult attention-deficit hyperactivity disorder % (95%UI se)= 3·2 (95%UI 3·4) |
|  | Kadzin 2023- Spain | nationally representative | Cross-sectional: World Mental Health Surveys report | None specified | major depressive disorder, anxiety disorders | 2001-2002 | 5473 | 18-98 y | Prevalence of ADM use by MDD and anxiety disorder histories (95%UI 12-month MDD), % (95%UI SE)= 28·5 (95%UI 3·3) and 17·0 (95%UI 3·0) |
|  | Mortier 2024 | Various regions (95%UI 6 Autonomous Communities) | Prospective cohort study on mental health service use among HCW during COVID-19 | Healthcare workers with positive screens for mental disorders or suicidal thoughts and behaviors | Depression, anxiety, PTSD, substance use disorder | May 2020 – September 2021 | 4,809 HCW at baseline | females (95%UI 80·5 %); median age 45·2 (95%UI IQR 35·5–52·9)· | Service use from 18·2% to 29·6% over 16 months· The most often used types of services were a psychiatrist or psychologist (95%UI range 10·5–19·9 % across T1-T4), a family physician (95%UI range 7·3–13·9 %), followed by occupational health services (95%UI range 2·9–5·9 %), primary care emergency visits (95%UI range 1·0–2·3 %), and hospital emergency department visits (95%UI range 0·5–1·2 %)· Odds for service use more than doubled (95%UI OR = 2·07) over the 16-month follow-up period in the fully adjusted multivariable model (95%UI Table 1 - left pane)· In this same model, service use was negatively associated with being female (95%UI OR = 0·69) and with higher daily number of work hours (95%UI OR = 0·95), and positively associated with pre-pandemic use of medication or psychological help (95%UI OR = 1·99), MDD (95%UI OR = 1·50), panic attacks (95%UI OR = 1·74), STB (95%UI OR = 1·22)) and severe role impairment (95%UI OR = 1·33) |
|  | Santomauro 2024 - Spain | nationally representative | modelling study | None specified | major depressive disorder | 2000 and 2021 | 1312800 (95%UI 2000) 2237000 (95%UI 2021) | >= 18 y | Proportion of persons with major depressive disorder receiving minimally adequate treatment by sex and location for 2000 and 2021 (95%UI 95% UIs): 23·3 (95%UI 17·4–31·7) and 23·8 (95%UI 17·9–33·3) |
|  | Stein 2021- Spain | nationally representative | cross sectional - WHO World Mental Health Survey Initiative | None specified | GAD (95%UI generalized anxiety disorder) | 2001-2002 | all participants (95%UI not only diagnosed)= 2121 | 18-96 y | Proportion of Respondents with lifetime GAD, who obtained treatment % (95%UI se)= 36·3 (95%UI 5·0) |
|  | Alonso 2018 - Spain | nationally representative | cross sectional - WHO World Mental Health Survey Initiative | None specified | anxiety (95%UI agoraphobia, generalized anxiety disorder, panic disorder, PTSD, social phobia, specific phobia, adult separation anxiety disorder)· | 2001-2002 | 5473 | 18-98 | Any treatment among those with disorder % (95%UI se); possibly adequate treatment % (95%UI se)= 29·5 (95%UI 2·6); 11·9 (95%UI 2·0) |
|  | Stein 2023 -Spain | nationally representative | Cross-sectional: World Mental Health Surveys report | None specified | PTSD | 2001-2002 | 5473 | 18-98 y | missing |
| Spain- Murcia | Alonso 2018 - Spain- Murcia | regionally representative | cross sectional - WHO World Mental Health Survey Initiative | None specified | anxiety (95%UI agoraphobia, generalized anxiety disorder, panic disorder, PTSD, social phobia, specific phobia, adult separation anxiety disorder)· | 2010-2012 | 2621 | 18-96 | Any treatment among those with disorder % (95%UI se); possibly adequate treatment % (95%UI se)= 45·1 (95%UI 3·1); 10·5 (95%UI 1·8) |
|  | de Vries 2021- Spain- Murcia | regionally representative samples | cross-sectional: World Mental Health Surveys report | None specified | specific phobia | 2010-2012 | all participants (95%UI not only diagnosed)= 2621 | 18-96 y | Respondents who obtained treatment for specific phobia %(95%UI se): 13·6 (95%UI 3·4) |
|  | Degenhard 2017 - Spain- Murcia | regionally representative | cross sectional - WHO World Mental Health Survey Initiative | None specified | Substance use disorders | 2010-2012 | 2621 | 18-96 y | Minimally adequate treatment among allthose with substanceuse disorders % = 35·3 |
|  | Fayyad 2017 - Spain- Murcia | regionally representative | cross sectional - WHO World Mental Health Survey Initiative | None specified | ADHD | 2010-2012 | 2621 | 18-96 y | Twelve-month treatment (95%UI any mental health treatment) among respondents with multiply imputed DSM-IV/CIDI adult attention-deficit hyperactivity disorder % (95%UI se)= 0·0 (95%UI 0·0) |
|  | Harris 2024 - Spain Murcia | regionally representative | cross-sectional: World Mental Health Surveys report | None specified | major depressive disorder, bipolar spectrum disorder, panic disorder/agoraphobia, generalized anxiety disorder, posttraumatic stress disorder, social phobia, specific phobia, and substance use disorders (95%UI alcohol and illicit drug abuse with or without dependence) | 2010-2012 | 7312 | 18-96 y | 12-month use of providers for mental health among those with any DSM-IV 12-month disorders: 40,9% (95%UI SE=3,0) |
|  | Kadzin 2023- Spain- Murcia | regionally representative | Cross-sectional: World Mental Health Surveys report | None specified | major depressive disorder, anxiety disorders | 2010-2012 | 2621 | 18-96 y | Prevalence of ADM use by MDD and anxiety disorder histories (95%UI 12-month MDD), % (95%UI SE)= 23·3 (95%UI 6·3) and 13·8 (95%UI 2·2) |
|  | Stein 2021- Spain- Murcia | regionally representative samples | cross sectional - WHO World Mental Health Survey Initiative | None specified | GAD (95%UI generalized anxiety disorder) | 2010-2012 | all participants (95%UI not only diagnosed)= 2621 | 18-96 y | Proportion of Respondents with lifetime GAD, who obtained treatment % (95%UI se)= 37·5 (95%UI 4·6) |
|  | Stein 2023 -Spain- Murcia | regionally representative | Cross-sectional: World Mental Health Surveys report | None specified | PTSD | 2010-2012 | 2621 | 18-96 y | among respondents with lifetime PTSD, % (95%UI SE) obtained treatment =19·1 (95%UI 3·7) |
| Sweden | ATLAS -Sweden | nationally representative | ATLAS | unclear | Psychosis | 2017 and 2020 | 10036391 | unclear | service coverage for psychosis 2017 and 2020 % = 49·38 and 59·74 |
|  | Öberg 2024 | Nationally representative survey | nationwide retrospective explorative study investigating medical records· | suicides | unclear | 2015 | 144 | Age, years at the time of suicide (95%UI Mean ± SD)= 58·1 ± 19; 32% female, occupied 40%· Table 1 | 49/105 individuals with any mental disorders had no contact in the previous 2 years with psychiatric services· 25/55 with mood disorders, 25/54 with neurotic, stress-related, somatoform, |
|  | Santomauro 2024 - Sweden | nationally representative | modelling study | None specified | major depressive disorder | 2000 and 2021 | 341600 (95%UI 2000) 436900 (95%UI 2021) | >= 18 y | Proportion of persons with major depressive disorder receiving minimally adequate treatment by sex and location for 2000 and 2021 (95%UI 95% UIs): 27·2 (95%UI 19·9–38·2) and 27·7 (95%UI 20·5–39·3) |
|  | Wallerblad 2012 | residing in the Stockholm County | longitudinal population-based | Swedish citizens | Depression and anxiety disorders | 1998-2010 | 2026 participants | median age 45, 58% female | Of those affected by depression and/or anxiety, 47·1% of the persons stated that they had been in contact with some type of health care facility within the last year due to psychological symptoms· Notes for details of type of care· |
| Switzerland | Mohler-Kuo 2016 | Nationally representative survey | cross-sectional household survey with computer-assisted telephone interview | General adult population + adolescents; Swiss residents from all three languages | eating disorders | April - October 2010 | 10,038 participants | 81·6% Swiss, 52% female, and 87·4% employed | Among those meeting the criteria for any ED, only 49·4% of males and 67·9% of females had ever sought professional help about their problems with eating or weight· |
|  | Santomauro 2024 - Switzerland | nationally representative | modelling study | None specified | major depressive disorder | 2000 and 2021 | 267500 (95%UI 2000) 359500 (95%UI 2021) | >= 18 y | Proportion of persons with major depressive disorder receiving minimally adequate treatment by sex and location for 2000 and 2021 (95%UI 95% UIs): 26·6 (95%UI 18·9–37·9) and 27·9 (95%UI 20·6–38·7) |
|  | Werlen 2020 | Nationally representative | cross-sectional | Young adults | Anxiety, depression, ADHD, risky substance use | 2018 | 3840 participants | average age 19·6 years, 49·5% female, 81·3% Swiss nationality | Among those with a CMD, only around half perceived lifetime need for care, and less than 20% reported currently utilizing mental health services· ADHD alone 9·4% (95%UI 4·1–14·8), Anxiety alone 11·7% (95%UI 5·7–17·6); Depression alone 15·9% (95%UI 11·3–20·4)· |
| Tajikistan | Santomauro 2024 - Tajikistan | nationally representative | modelling study | None specified | major depressive disorder | 2000 and 2021 | 86200 (95%UI 2000) 19500 (95%UI 2021) | >= 18 y | Proportion of persons with major depressive disorder receiving minimally adequate treatment by sex and location for 2000 and 2021 (95%UI 95% UIs): 8·8 (95%UI 6·2–12·6) and 9·8 (95%UI 7·0–14·1) |
| the Netherlands | ATLAS -Netherlands | nationally representative | ATLAS | unclear | Psychosis | 2017 and 2020 | 17097123 | unclear | service coverage for psychosis 2017 % = 23·40 |
|  | Alonso 2018 - the Netherlands | nationally representative | cross sectional - WHO World Mental Health Survey Initiative | None specified | anxiety (95%UI agoraphobia, generalized anxiety disorder, panic disorder, PTSD, social phobia, specific phobia, adult separation anxiety disorder)· | 2002-2003 | 2372 | 18-95 y | Any treatment among those with disorder % (95%UI se); possibly adequate treatment % (95%UI se)= 31·6 (95%UI 5·5); 16·1 (95%UI 3·5) |
|  | Boerema 2016 | three Municipal Health Services (95%UI GGD) across different regions in the Netherlands (95%UI Amsterdam, Zoetermeer/Leidschendam, Dordrecht/ Gorinchem) | cross-sectional study | responded to the survey | major depressive disorder, anxiety disorders | 2012 | 291 responded (95%UI 102 were diagnosed with depression) | The sample consisted of 55 females (95%UI 54 %) and 47 males (95%UI 46 %)· Respondents were 52 years of age on average (95%UI range 20–88)· 46 % of the participants reported co-morbidity with an anxiety disorder in the past six months | Of the participants who had received help for psychological problems in the past six months (95%UI n = 66), 26 % (95%UI n = 17) received help in general health care (95%UI general practitioner, social work, medical specialist), 15 % (95%UI n = 10) received help in specialized mental health care (95%UI psychiatrist, psychologist, clinic for alcohol or drugs abuse, mental health institution, psychiatrist in hospital)· The majority 59 % (95%UI n = 39) received help in both settings· |
|  | de Vries 2021- the Netherlands | nationally representative | cross-sectional: World Mental Health Surveys report | None specified | specific phobia | 2002-2003 | all participants (95%UI not only diagnosed)= 1094 | 18-95 y | Respondents who obtained treatment for specific phobia %(95%UI se): 23·0 (95%UI 4·8) |
|  | Degenhard 2017 - the Netherlands | nationally representative | cross sectional - WHO World Mental Health Survey Initiative | None specified | Substance use disorders | 2002-2003 | 2372 | 18-95 y | Minimally adequate treatment among allthose with substanceuse disorders % (95%UI se) = 4·2 (95%UI 0·9) |
|  | Fayyad 2017 - the Netherlands | nationally representative | cross sectional - WHO World Mental Health Survey Initiative | None specified | ADHD | 2002-2003 | 2372 | 18-95 y | Twelve-month treatment (95%UI any mental health treatment) among respondents with multiply imputed DSM-IV/CIDI adult attention-deficit hyperactivity disorder % (95%UI se)= 1·9 (95%UI 1·7) |
|  | Kadzin 2023- the Netherlands | nationally representative | Cross-sectional: World Mental Health Surveys report | None specified | major depressive disorder, anxiety disorders | 2002-2003 | 2372 | 18-95 y | Prevalence of ADM use by MDD and anxiety disorder histories (95%UI 12-month MDD), % (95%UI SE)= 29·9 (95%UI 6·3) and 18·0 (95%UI 3·9) |
|  | Santomauro 2024 - Netherlands | nationally representative | modelling study | None specified | major depressive disorder | 2000 and 2021 | 471500 (95%UI 2000) 627900 (95%UI 2021) | >= 18 y | Proportion of persons with major depressive disorder receiving minimally adequate treatment by sex and location for 2000 and 2021 (95%UI 95% UIs): 31·2 (95%UI 24·0–39·2) and 30·3 (95%UI 23·8–39·7) |
|  | Stein 2021- the Netherlands | nationally representative | cross sectional - WHO World Mental Health Survey Initiative | None specified | GAD (95%UI generalized anxiety disorder) | 2002-2003 | all participants (95%UI not only diagnosed)= 1094 | 18-95 y | Proportion of Respondents with lifetime GAD, who obtained treatment % (95%UI se)= 46·4 (95%UI 6·3) |
|  | Stein 2023 -the Netherlands | nationally representative | Cross-sectional: World Mental Health Surveys report | None specified | PTSD | 2002-2003 | 2372 | 18-95 y | missing |
|  | ten Have 2004 | Nationally representative | Netherlands Mental Health Survey (95%UI NEMESIS) | None specified | major or minor depression | February - December 1996 | 1572 participants | Mean age (95%UI sd) =40·6 (95%UI 11·1); Female 65·6% | In terms of service utilisation for mental health problems, 28% of the depressed respondents had used exclusively primary care services, 45% had used specialised mental health care·  27% had never received any professional help for their mental health problems |
|  | Tuithof 2016 | Nationally representative | prospective epidemiologic survey: Netherlands Mental Health Survey and Incidence Study-2 (95%UI NEMESIS-2) | None specified | mild to severe Alcohol use disorder (95%UI AUD) | 2007–2011 | 154 participants | mean age (95%UI sd) = 34·0 (95%UI 1·3); female 33·4% | 10% specialized treatment, 35% general mental health support·  55% untreated· Higher gap in mild AUD cases· |
| Turkey | Kilic 2024 | Ankara city | cross-sectional mixed-method study | 420 Syrian refugees living in Ankara city center | PTSD, depression | 2016 | 420 | mean age of the participants in the study was 35·4 (95%UI Range: 18-80, SD: 13·0)· 56·4% females and 84·7% married | 9·7% of those who felt they needed help contacted the menatl health service· |
|  | Santomauro 2024 - Turkey | nationally representative | Modelling study | >= 18 y | None specified | major depressive disorder | 2000 and 2021 | >= 18 y | Proportion of persons with major depressive disorder receiving minimally adequate treatment by sex and location for 2000 and 2021 (95% UIs): 4·6 (3·1–6·9) and 5·5 (3·8–7·9) |
| Turkmenistan | Santomauro 2024 - Turkmenistan | nationally representative | modelling study | None specified | major depressive disorder | 2000 and 2021 | 75400 (2000) 12100 (2021) | >= 18 y | Proportion of persons with major depressive disorder receiving minimally adequate treatment by sex and location for 2000 and 2021 (95%UI 95% UIs): 9·0 (95%UI 6·4–12·8) and 9·9 (95%UI 7·0–14·1) |
| Ukraine | Degenhard 2017 - Ukraine | nationally representative | cross sectional - WHO World Mental Health Survey Initiative | None specified | Substance use disorders | 2002 | 4725 | 18-91 y | Minimally adequate treatment among allthose with substanceuse disorders % (95%UI se) = 0·6 (95%UI 0·6) |
|  | Roberts 2017 |  | cross-sectional | internally displaced | PTSD, depression, anxiety | March - May 2016 | 2203 | 68% females, age range (95%UI n (95%UI %)): 18–30 480 (95%UI 21·8); 31–44 711 (95%UI 32·3); 45–59 522 (95%UI 23·6); 60–74 356 (95%UI 16·2) | Of the 703 respondents who reported having a mental health or emotional problem over the previous 12 months and who also screened positive with PTSD, depression, or anxiety, 520 respondents did not seek care (95%UI there were missing data for the remaining three respondents)· This equates to an overall treatment gap of 74% (95%UI 95% CI 70·99–77·55)· When broken down by condition, the treatment gap was 74% (95%UI 95% CI 70·47–77·60) for PTSD, 69% (95%UI 95% CI 64·31–74·05) for depression, and 68% (95%UI 95% CI 62·25–72·99) for anxiety· There were no statistically significant differences in the treatment gap between males and females |
|  | Santomauro 2024 - Ukraine | nationally representative | modelling study | None specified | major depressive disorder | 2000 and 2021 | 2205400 (95%UI 2000) 2093700 (95%UI 2021) | >= 18 y | Proportion of persons with major depressive disorder receiving minimally adequate treatment by sex and location for 2000 and 2021 (95%UI 95% UIs): 11·8 (95%UI 9·7–14·3) and 12·6 (95%UI 10·6–15·3) |
| Uzbekistan | ATLAS -Uzbekistan | nationally representative | ATLAS | unclear | Psychosis | 2017 and 2020 | 32981715 | unclear | service coverage for psychosis 2017 % = 56·50 |
|  | Santomauro 2024 - Uzbekistan | nationally representative | modelling study | None specified | major depressive disorder | 2000 and 2021 | 466900 (95%UI 2000) 88500 (95%UI 2021) |  | Proportion of persons with major depressive disorder receiving minimally adequate treatment by sex and location for 2000 and 2021 (95%UI 95% UIs): 9·4 (95%UI 6·6–13·3) and 10·6 (95%UI 7·6–15·0) |
| UK- Wales | Santomauro 2024 - Wales | nationally representative | modelling study | None specified | major depressive disorder | 2000 and 2021 | 108600 (95%UI 2000) 135700 (95%UI 2021) | >= 18 y | Proportion of persons with major depressive disorder receiving minimally adequate treatment by sex and location for 2000 and 2021 (95%UI 95% UIs): 27·9 (95%UI 20·6–38·7) and 25·0 (95%UI 18·3–35·0) |

# **References of included studies**

Aagaard, J., & Nielsen, J. A. (2004). Experience from the first ACT programme in Denmark. I. Baseline evaluation 1981-2000. *Nordic Journal of Psychiatry*, *58*(2), 165–169. https://doi.org/10.1080/08039480410005567

Alonso, J., Liu, Z., Evans-Lacko, S., Sadikova, E., Sampson, N., Chatterji, S., Abdulmalik, J., Aguilar-Gaxiola, S., Al-Hamzawi, A., Andrade, L. H., Bruffaerts, R., Cardoso, G., Cia, A., Florescu, S., de Girolamo, G., Gureje, O., Haro, J. M., He, Y., de Jonge, P., … Thornicroft, G. (2018). Treatment gap for anxiety disorders is global: Results of the World Mental Health Surveys in 21 countries. *Depression and Anxiety*, *35*(3), 195–208. https://doi.org/10.1002/da.22711

Ballester, L., Alayo, I., Vilagut, G., Almenara, J., Cebrià, A. I., Echeburúa, E., Gabilondo, A., Gili, M., Lagares, C., Piqueras, J. A., Roca, M., Soto-Sanz, V., Blasco, M. J., Castellví, P., Mortier, P., Bruffaerts, R., Auerbach, R. P., Nock, M. K., Kessler, R. C., & Jordi, A. (2020). Mental disorders in Spanish university students: Prevalence, age-of-onset, severe role impairment and mental health treatment. *Journal of Affective Disorders*, *273*, 604–613. https://doi.org/10.1016/j.jad.2020.04.050

Barbato, A., Vallarino, M., Rapisarda, F., Lora, A., Parabiaghi, A., D’Avanzo, B., & Lesage, A. (2016). Do people with bipolar disorders have access to psychosocial treatments? A survey in Italy. *International Journal of Social Psychiatry*, *62*(4), 334–344. https://doi.org/10.1177/0020764016631368

Boerema, A. M., Kleiboer, A., Beekman, A. T. F., van Zoonen, K., Dijkshoorn, H., & Cuijpers, P. (2016). Determinants of help-seeking behavior in depression: A cross-sectional study. *BMC Psychiatry*, *16*(1). https://doi.org/10.1186/s12888-016-0790-0

Brazinova, A., Hasto, J., Levav, I., & Pathare, S. (2019). Mental Health Care Gap: The Case of the Slovak Republic. *Administration and Policy in Mental Health and Mental Health Services Research*, *46*(6), 753–759. https://doi.org/10.1007/s10488-019-00952-z

Bruffaerts, R., Harris, M. G., Kazdin, A. E., Vigo, D. v, Sampson, N. A., Tat Chiu, W., Al-Hamzawi, A., Alonso, J., Altwaijri, Y. A., Andrade, L., Benjet, C., de Girolamo, G., Florescu, S., Maria Haro, J., Hu, C., Karam, A., Karam, E. G., Kovess-Masfety, V., Lee, S., … Williams, D. R. (n.d.). *Perceived Helpfulness of Treatment For Social Anxiety Disorder Findings From The WHO World Mental Health Surveys HHS Public Access*.

Cook, S., Kudryavtsev, A. v., Bobrova, N., Saburova, L., Denisova, D., Malyutina, S., Lewis, G., & Leon, D. A. (2020). Prevalence of symptoms, ever having received a diagnosis and treatment of depression and anxiety, and associations with health service use amongst the general population in two Russian cities. *BMC Psychiatry*, *20*(1). https://doi.org/10.1186/s12888-020-02938-w

Corrao, G., Monzio Compagnoni, M., Barbato, A., D’Avanzo, B., di Fiandra, T., Ferrara, L., Gaddini, A., Saponaro, A., Scondotto, S., Tozzi, V. D., Carle, F., Carbone, S., Chisholm, D. H., & Lora, A. (2022). From contact coverage to effective coverage of community care for patients with severe mental disorders: A real-world investigation from Italy. *Frontiers in Psychiatry*, *13*. https://doi.org/10.3389/fpsyt.2022.1014193

Dauriac Le-Masson, V., Mercuel, A., Guedj, M. J., Douay, C., Chauvin, P., & Laporte, A. (2020). Mental healthcare utilization among homeless people in the greater paris area. *International Journal of Environmental Research and Public Health*, *17*(21), 1–13. https://doi.org/10.3390/ijerph17218144

de Vries, Y. A., Harris, M. G., Vigo, D., Chiu, W. T., Sampson, N. A., Al-Hamzawi, A., Alonso, J., Andrade, L. H., Benjet, C., Bruffaerts, R., Bunting, B., Caldas de Almeida, J. M., de Girolamo, G., Florescu, S., Gureje, O., Haro, J. M., Hu, C., Karam, E. G., Kawakami, N., … de Jonge, P. (2021). Perceived helpfulness of treatment for specific phobia: Findings from the World Mental Health Surveys. *Journal of Affective Disorders*, *288*, 199–209. https://doi.org/10.1016/j.jad.2021.04.001

Degenhardt, L., Glantz, M., Evans-Lacko, S., Sadikova, E., Sampson, N., Thornicroft, G., Aguilar-Gaxiola, S., Al-Hamzawi, A., Alonso, J., Helena Andrade, L., Bruffaerts, R., Bunting, B., Bromet, E. J., Caldas de Almeida, J. M., de Girolamo, G., Florescu, S., Gureje, O., Maria Haro, J., Huang, Y., … Zaslavsky, A. M. (2017). Estimating treatment coverage for people with substance use disorders: an analysis of data from the World Mental Health Surveys. *World Psychiatry*, *16*(3), 299–307. https://doi.org/10.1002/wps.20457

Demyttenaere, K., Bonnewyn, A., Bruffaerts, R., de Graaf, R., Haro, J. M., & Alonso, J. (2008). Comorbid painful physical symptoms and anxiety: Prevalence, work loss and help-seeking. *Journal of Affective Disorders*, *109*(3), 264–272. https://doi.org/10.1016/j.jad.2007.12.231

Fayyad, J., Sampson, N. A., Hwang, I., Adamowski, T., Aguilar-Gaxiola, S., Al-Hamzawi, A., Andrade, L. H. S. G., Borges, G., de Girolamo, G., Florescu, S., Gureje, O., Haro, J. M., Hu, C., Karam, E. G., Lee, S., Navarro-Mateu, F., O’Neill, S., Pennell, B. E., Piazza, M., … Wojtyniak, B. (2017). The descriptive epidemiology of DSM-IV Adult ADHD in the World Health Organization World Mental Health Surveys. *ADHD Attention Deficit and Hyperactivity Disorders*, *9*(1), 47–65. https://doi.org/10.1007/s12402-016-0208-3

Font, H., Roelandt, J. L., Behal, H., Geoffroy, P. A., Pignon, B., Amad, A., Simioni, N., Vaiva, G., Thomas, P., Duhamel, A., Benradia, I., & Rolland, B. (2018). Prevalence and predictors of no lifetime utilization of mental health treatment among people with mental disorders in France: findings from the ‘Mental Health in General Population’ (MHGP) survey. *Social Psychiatry and Psychiatric Epidemiology*, *53*(6), 567–576. https://doi.org/10.1007/s00127-018-1507-0

Grigaitė, U., Azeredo-Lopes, S., Cardoso, G., Pedrosa, B., Aluh, D. O., Santos-Dias, M., Silva, M., Xavier, M., & Caldas-de-Almeida, J. M. (2024). Mental health conditions and utilisation of mental health services by survivors of physical intimate partner violence in Portugal: Results from the WHO world mental health survey. *Psychiatry Research*, *334*. https://doi.org/10.1016/j.psychres.2024.115801

Hämäläinen, J., Isometsä, E., Sihvo, S., Pirkola, S., & Kiviruusu, O. (2008). Use of health services for major depressive and anxiety disorders in Finland. *Depression and Anxiety*, *25*(1), 27–37. https://doi.org/10.1002/da.20256

Harris, M. G., Kazdin, A. E., Munthali, R. J., Vigo, D. v., Stein, D. J., Viana, M. C., Aguilar-Gaxiola, S., Al-Hamzawi, A., Alonso, J., Andrade, L. H., Bunting, B., Chardoul, S., Gureje, O., Hu, C., Hwang, I., Karam, E. G., Navarro-Mateu, F., Nishi, D., Orozco, R., … Kessler, R. C. (2024). Factors associated with satisfaction and perceived helpfulness of mental healthcare: a World Mental Health Surveys report. *International Journal of Mental Health Systems*, *18*(1). https://doi.org/10.1186/s13033-024-00629-7

*IAPT three-year report – The first million patients*. (2012). www.dh.gsi.gov.uk

Kagstrom, A., Alexova, A., Tuskova, E., Csajbók, Z., Schomerus, G., Formanek, T., Mladá, K., Winkler, P., & Cermakova, P. (2019). The treatment gap for mental disorders and associated factors in the Czech Republic. *European Psychiatry*, *59*, 37–43. https://doi.org/10.1016/j.eurpsy.2019.04.003

Kazdin, A. E., Wu, C. S., Hwang, I., Puac-Polanco, V., Sampson, N. A., Al-Hamzawi, A., Alonso, J., Andrade, L. H., Benjet, C., Caldas-De-Almeida, J. M., de Girolamo, G., de Jonge, P., Florescu, S., Gureje, O., Haro, J. M., Harris, M. G., Karam, E. G., Karam, G., Kovess-Masfety, V., … Atwoli, L. (2023). Antidepressant use in low- middle- and high-income countries: A World Mental Health Surveys report. *Psychological Medicine*, *53*(4), 1583–1591. https://doi.org/10.1017/S0033291721003160

KILIÇ, C., KAYA, E., KARADAĞ, Ö., & ÜNER, S. (2024). Barriers to Accessing Mental Health Services Among Syrian Refugees: A Mixed-Method Study. *Turk Psikiyatri Dergisi*, *35*(2), 87–94. https://doi.org/10.5080/u27044

Lora, A., Barbato, A., Cerati, G., Erlicher, A., & Percudani, M. (2012). The mental health system in Lombardy, Italy: Access to services and patterns of care. *Social Psychiatry and Psychiatric Epidemiology*, *47*(3), 447–454. https://doi.org/10.1007/s00127-011-0352-1

Mohler-Kuo, M., Schnyder, U., Dermota, P., Wei, W., & Milos, G. (2016). The prevalence, correlates, and help-seeking of eating disorders in Switzerland. *Psychological Medicine*, *46*(13), 2749–2758. https://doi.org/10.1017/S0033291716001136

Mortier, P., Vilagut, G., García-Mieres, H., Alayo, I., Ferrer, M., Amigo, F., Aragonès, E., Aragón-Peña, A., Asúnsolo del Barco, Á., Campos, M., Espuga, M., González-Pinto, A., Haro, J. M., López Fresneña, N., Martínez de Salázar, A. D., Molina, J. D., Ortí-Lucas, R. M., Parellada, M., Pelayo-Terán, J. M., … Alonso, J. (2024). Health service and psychotropic medication use for mental health conditions among healthcare workers active during the Spain Covid-19 Pandemic – A prospective cohort study using web-based surveys. *Psychiatry Research*, *334*. https://doi.org/10.1016/j.psychres.2024.115800

Murphy, A., Chikovani, I., Uchaneishvili, M., Makhashvili, N., & Roberts, B. (2018). Barriers to mental health care utilization among internally displaced persons in the republic of Georgia: A rapid appraisal study. *BMC Health Services Research*, *18*(1). https://doi.org/10.1186/s12913-018-3113-y

Öberg, N. P., Lindström, S. P., Bergqvist, E., Ehnvall, A., Sellin, T., Stefenson, A., Sunnqvist, C., Waern, M., & Westrin, Å. (2024). Last general practitioner consultation during the final month of life: a national medical record review of suicides in Sweden. *BMC Primary Care*, *25*(1). https://doi.org/10.1186/s12875-024-02498-y

Pitonák, M., Potočár, L., & Formánek, T. (2024). Mental health and help-seeking in Czech sexual minorities: A nationally representative cross-sectional study. *Epidemiology and Psychiatric Sciences*, *33*. https://doi.org/10.1017/S2045796024000210

Potočár, L., Mladá, K., Kučera, M., Mohr, P., Winkler, P., & Formánek, T. (2024). Population mental health, help-seeking and associated barriers following the COVID-19 pandemic: Analysis of repeated nationally representative cross-sectional surveys in Czechia. *Psychiatry Research*, *331*. https://doi.org/10.1016/j.psychres.2023.115641

Probst, C., Manthey, J., Martinez, A., & Rehm, J. (2015). Alcohol use disorder severity and reported reasons not to seek treatment: A cross-sectional study in European primary care practices. *Substance Abuse: Treatment, Prevention, and Policy*, *10*(1). https://doi.org/10.1186/s13011-015-0028-z

Reich, H., Niermann, H. C. M., Voss, C., Venz, J., Pieper, L., & Beesdo-Baum, K. (2024). Sociodemographic, psychological, and clinical characteristics associated with health service (non-)use for mental disorders in adolescents and young adults from the general population. *European Child and Adolescent Psychiatry*, *33*(2), 391–400. https://doi.org/10.1007/s00787-023-02146-3

Rens, E., Dom, G., Remmen, R., Michielsen, J., & van den Broeck, K. (2020). Unmet mental health needs in the general population: Perspectives of Belgian health and social care professionals. *International Journal for Equity in Health*, *19*(1). https://doi.org/10.1186/s12939-020-01287-0

Roberts, B., Makhashvili, N., Javakhishvili, J., Karachevskyy, A., Kharchenko, N., Shpiker, M., & Richardson, E. (2019). Mental health care utilisation among internally displaced persons in Ukraine: Results from a nation-wide survey. *Epidemiology and Psychiatric Sciences*, *28*(1), 100–111. https://doi.org/10.1017/S2045796017000385

Santomauro, D. F., Vos, T., Whiteford, H. A., Chisholm, D., Saxena, S., & Ferrari, A. J. (2024). Service coverage for major depressive disorder: estimated rates of minimally adequate treatment for 204 countries and territories in 2021. *The Lancet Psychiatry*, *11*(12), 1012–1021. https://doi.org/10.1016/S2215-0366(24)00317-1

Sebela, A., Byatt, & N., Formanek, & T., & Winkler, & P. (n.d.). *Prevalence of mental disorders and treatment gap among Czech females during paid maternity or parental leave*. https://doi.org/10.1007/s00737-020-01052-w/Published

Silva, M., Antunes, A., Azeredo-Lopes, S., Cardoso, G., Xavier, M., Saraceno, B., & Caldas-de-Almeida, J. M. (2022). Barriers to mental health services utilisation in Portugal–results from the National Mental Health Survey. *Journal of Mental Health*, *31*(4), 453–461. https://doi.org/10.1080/09638237.2020.1739249

Stein, D. J., Harris, M., Vigo, D., Tat Chiu, W., Sampson, N., Alonso, J., Altwaijri, Y., Bunting, B., Caldas-de-Almeida, J. M., Cía, A., Ciutan, M., Degenhardt, L., Gureje, O., Karam, A., Karam, E. G., Lee, S., Medina-Mora, M. E., Mneimneh, Z., Navarro-Mateu, F., … Wojtyniak, B. (2020). Perceived helpfulness of treatment for posttraumatic stress disorder: Findings from the World Mental Health Surveys. *Depression and Anxiety*, *37*(10), 972–994. https://doi.org/10.1002/da.23076

Stein, D. J., Kazdin, A. E., Munthali, R. J., Hwang, I., Harris, M. G., Alonso, J., Andrade, L. H., Bruffaerts, R., Cardoso, G., Chardoul, S., de Girolamo, G., Florescu, S., Gureje, O., Haro, J. M., Karam, A. N., Karam, E. G., Kovess-Masfety, V., Lee, S., Medina-Mora, M. E., … Wojtyniak, B. (2023). Determinants of effective treatment coverage for posttraumatic stress disorder: findings from the World Mental Health Surveys. *BMC Psychiatry*, *23*(1). https://doi.org/10.1186/s12888-023-04605-2

Stein, D. J., Kazdin, A. E., Ruscio, A. M., Chiu, W. T., Sampson, N. A., Ziobrowski, H. N., Aguilar-Gaxiola, S., Al-Hamzawi, A., Alonso, J., Altwaijri, Y., Bruffaerts, R., Bunting, B., de Girolamo, G., de Jonge, P., Degenhardt, L., Gureje, O., Haro, J. M., Harris, M. G., Karam, A., … Wojtyniak, B. (2021). Perceived helpfulness of treatment for generalized anxiety disorder: a World Mental Health Surveys report. *BMC Psychiatry*, *21*(1). https://doi.org/10.1186/s12888-021-03363-3

Stolzenburg, S., Freitag, S., Evans-Lacko, S., Speerforck, S., Schmidt, S., & Schomerus, G. (2019). Individuals with currently untreated mental illness: Causal beliefs and readiness to seek help. *Epidemiology and Psychiatric Sciences*, *28*(4), 446–457. https://doi.org/10.1017/S2045796017000828

ten Have, M., de Graaf, R., Vollebergh, W., & Beekman, A. (2004). What depressive symptoms are associated with the use of care services? Results from the Netherlands Mental Health Survey and Incidence Study (NEMESIS). *Journal of Affective Disorders*, *80*(2–3), 239–248. https://doi.org/10.1016/S0165-0327(03)00132-0

Tuithof, M., ten Have, M., van den Brink, W., Vollebergh, W., & de Graaf, R. (2016). Treatment Seeking for Alcohol Use Disorders: Treatment Gap or Adequate Self-Selection? *European Addiction Research*, *22*(5), 277–285. https://doi.org/10.1159/000446822

Wallerblad, A., Möller, J., & Forsell, Y. (2012). Care-Seeking Pattern among Persons with Depression and Anxiety: A Population-Based Study in Sweden. *International Journal of Family Medicine*, *2012*, 1–9. https://doi.org/10.1155/2012/895425

Werlen, L., Puhan, M. A., Landolt, M. A., & Mohler-Kuo, M. (2020). Mind the treatment gap: The prevalence of common mental disorder symptoms, risky substance use and service utilization among young Swiss adults. *BMC Public Health*, *20*(1). https://doi.org/10.1186/s12889-020-09577-6

World Health Organization. (2020). *WHO ATLAS 2020*.

.

**Table S2. Percentage of coverage for each country and each mental disorder**

| **Country** | **Any Mental disorder** | **Psychoses** | **Major depressive Didorder** | **Anxiety disorders** | **ADHD** | **SUD** | **PTSD** |
| --- | --- | --- | --- | --- | --- | --- | --- |
| **Albania** | NA | NA | 13·6 (95%UI 10·4–18·2)  [2021] | NA | NA | NA | NA |
| **Andorra** | NA | NA | 27·7 (95%UI 19·7–40·0)  [2021] | NA | NA | NA | NA |
| **Armenia** | NA | NA | 12·0 (95%UI 8·7–16·6)  [2021] | NA | NA | NA | NA |
| **Austria** | NA | NA | 26·7 (95%UI 19·5–37·5)  [2021] | NA | NA | NA | NA |
| **Azerbaijan** | NA | 45·12  [2017] | 11·0 (95%UI 7·9–15·9)  [2021] | NA | NA | NA | NA |
| **Belarus** | NA | 64·9  [2020] | 13·5 (95%UI 9·8–19·1)  [2021] | NA | NA | NA | NA |
| **Belgium** | NA | NA | 34·4 (95%UI 25·2–47·6)  [2021] | 35·7 (SE= 3·8)  [2002] | 0·0 (SE= 0·0)  [2002] | 6·8 (SE= 1·5)  [2002] | NA |
| **Bosnia and Herzegovina** | NA | 37·11  [2017] | 12·8 (95%UI 9·4–17·9)  [2021] | NA | NA | NA | NA |
| **Bulgaria** | 12·1 (SE= 4·3)  [2017] | NA | 9·5 (95%UI 6·9–13·0)  [2021] | 21·6 (SE= 3·7)  [2006] | NA | 2·4 (SE= 0·2)  [2006] | 10·3 (95%UI 2·8)  [2017] |
| **Croatia** | NA | 99·45  [2017] | 13·8 (95%UI 10·1–19·1)  [2021] | NA | NA | NA | NA |
| **Cyprus** | NA | NA | 26·8 (95%UI 19·0–38·7)  [2021] | NA | NA | NA | NA |
| **Czechia** | 22·66 (95%UI= 20·83 - 24·49)  [2022] | 94  [2017] | 14·1 (95%UI 10·2–19·6)  [2021] | 35·03 (95%UI= 31·95 - 38·12)  [2022] | NA | 13·61 (95%UI= 11·29 - 15·93)  [2022] | NA |
| **Denmark** | 79  [2000] | 72·88  [2017] | 26·0 (95%UI 19·1–36·5)  [2021] | NA | NA | NA | NA |
| **UK- England** | 64·5  [2012] | NA | 25·8 (95%UI 21·7–31·2)  [2021] | NA | NA | NA | NA |
| **Estonia** | NA | NA | 13·7 (95%UI 10·0–19·0)  [2021] | NA | NA | NA | NA |
| **Finland** | NA | 97·43  [2020] | 20·6 (95%UI 15·2–29·0)  [2021] | 28  [2001] |  | NA | NA |
| **France** | 53·5  [2003] | 81·59  [2020] | 29·0 (95%UI 21·3–40·7)  [2021] | 29·4 (SE= 4·3)  [2002] | 0·0 (SE= 0·0)  [2002] | 14·9 (SE= 3·8) | NA |
| **Georgia** | NA | NA | 11·4 (95%UI 8·3–16·0)  [2021] |  | NA | NA | NA |
| **Germany** | NA | NA | 35·0 (95%UI 26·0–48·0)  [2021] | 24·0 (SE= 3·7)  [2003] | 0·0 (SE= 0·0)  [2003] | 8·2 (SE= 3·0)  [2003] | NA |
| **Greece** | NA | NA | 25·6 (95%UI= 18·8–35·8)  [2021] | NA | NA | NA | NA |
| **Hungary** | NA | NA | 13·4 (95%UI= 9·8–18·7)  [2021] | NA | NA | NA | NA |
| **Iceland** | NA | NA | 26·6 (95%UI= 19·3–37·1)  [2021] | NA | NA | NA | NA |
| **Ireland** | NA | NA | 30·1 (95%UI= 21·3–42·9)  [2021] | NA | NA | NA | NA |
| **Israel** | NA | NA | 20·0 (95%UI 14·6–28·3)  [2021] | 41·2 (SE= 3·9)  [2004] | NA | 3·4 (SE= 1·4)  [2004] | 12·0 (95%UI 4·1)  [2004] |
| **Italy** | NA | 58·85  [2020] | 19·5 (95%UI 16·6–23·4)  [2021] | 29·7 (SE= 3·3)  [2002] | 0·0 (SE= 0·0)  [2002] | 4·1 (SE= 0·6)  [2002] | NA |
| **Kazakhstan** | NA | NA | 11·4 (95%UI 8·2–15·9)  [2021] | NA | NA | NA | NA |
| **Kyrgyzstan** | NA | NA | 10·6 (95%UI 7·5–15·0)  [2021] | NA | NA | NA | NA |
| **Latvia** | NA | NA | 12·9 (95%UI 9·4–17·8)  [2021] | NA | NA | NA | NA |
| **Lithuania** | NA | NA | 12·9 (95%UI 9·5–18·2)  [2021] | NA | NA | NA | NA |
| **Luxembourg** | NA | NA | 26·9 (95%UI 19·3–38·2)  [2021] | NA | NA | NA | NA |
| **Malta** | NA | NA | 26·3 (95%UI 19·6–36·4)  [2021] | NA | NA | NA | NA |
| **Monaco** | NA | 83·68  [2020] | 26·2 (95%UI 19·4–36·7)  [2021] | NA | NA | NA | NA |
| **Montenegro** | NA | NA | 13·5 (95%UI 9·8–18·8)  [2021] | NA | NA | NA | NA |
| **Netherlands** | NA | 23·4  [2017] | 30·3 (95%UI 23·8–39·7)  [2021] | 31·6 (SE= 5·5)  [2003] | 1·9 (SE= 1·7)  [2003] | 4·2 (SE= 0·9)  [2003] | NA |
| **North Macedonia** | NA | NA | 12·8 (95%UI 9·3–17·8)  [2021] | NA | NA | NA | NA |
| **UK- Northern Ireland** | 50·6 (SE= 2·6)  [2008] | NA | 26·1 (95%UI 18·7–37·4)  [2021] | NA | 0·6 (SE= 0·6)  [2007] | 7·1 (SE= 2·0)  [2008] | 39·2 (95%UI 4·3)  [2008] |
| **Norway** | NA | 48·68  [2017] | 26·7 (95%UI 22·5–32·3)  [2021] | NA | NA | NA | NA |
| **Poland** | 18·2 (SE= 1·7)  [2011] | NA | 12·6 (95%UI 10·6–15·2)  [2021] | NA | 5·8 (SE= 5·7)  [2011] | 6·2 (SE= 1·8)  [2011] | NA |
| **Portugal** | 36·9 (SE= 2·2)  [2009] | NA | 21·8 (95%UI 16·1–30·5)  [2021] | 32·0 (SE= 2·4)  [2009] | 0·0 (SE= 0·0)  [2009] | 10·3 (SE= 6·2)  [2009] | 28·7 (95%UI 4·4)  [2009] |
| **Republic of Moldova** | NA | NA | 12·3 (95%UI 8·9–17·2)  [2021] | NA | NA | NA | NA |
| **Romania** | 20·0 (SE= 2·6)  [2006] | NA | 17·0 (95%UI 12·4–24·0)  [2021] | 29·2 (SE= 4·1)  [2006] | NA | NA | 6·9 (95%UI 5·2)  [2006] |
| **Russian federation** | NA | NA | 12·8 (95%UI 10·8–15·6)  [2021] | 1·5  [2018] | NA | NA | NA |
| **San Marino** | NA | NA | 27·3 (95%UI 20·0–38·7)  [2021] | NA | NA | NA | NA |
| **UK- Scotland** | NA | NA | 25·7 (95%UI 18·6–36·8)  [2021] | NA | NA | NA | NA |
| **Serbia** | NA | NA | 12·8 (95%UI 9·4–17·7)  [2021] | NA | NA | NA | NA |
| **Slovakia** | NA | 100 (range=56-100)  [2015] | 13·4 (95%UI 9·9–19·0)  [2021] | 15 (range=7·3 -23·7)  [2015] | NA | 20 (range=11·9–100)  [2015] | NA |
| **Slovenia** | NA | 64·98  [2020] | 15·0 (95%UI 10·9–20·7)  [2021] | NA | NA | NA | NA |
| **Spain** | NA | NA | 23·8 (95%UI 17·9–33·3)  [2021] | 29·5 (SE= 2·6)  [2002] | 3·2 (SE= 3·4)  [2002] | 5·2 (SE= 1·2)  [2002] | NA |
| **Spain- Murcia** | 40·9 (SE= 3·0)  [2012] | NA | 23·3 (95%UI 6·3)  [2012] | 45·1 (SE= 3·1)  [2012] | 0·0 (SE= 0·0)  [2012] | 35·3  [2012] | 19·1 (95%UI 3·7)  [2012] |
| **Sweden** | NA | 59·74  [2020] | 27·7 (95%UI 20·5–39·3)  [2021] | 47·1  [2010] | NA | NA | NA |
| **Switzerland** | NA | NA | 27·9 (95%UI 20·6–38·7)  [2021] | 11·7 (SE= 5·7–17·6)  [2018] | 9·4 (range= 4·1–14·8)  [2018] | NA | NA |
| **Tajikistan** | NA | NA | 9·8 (95%UI 7·0–14·1)  [2021] | NA | NA | NA | NA |
| **Turkey** | NA | NA | 5·5 (3·8–7·9)  [2021] | NA | NA | NA | 9·7 |
| **Turkmenistan** | NA | NA | 9·9 (95%UI 7·0–14·1)  [2021] | NA | NA | NA | NA |
| **Ukraine** | NA | NA | 12·6 (95%UI 10·6–15·3)  [2021] | NA | NA | 0·6 (SE= 0·6)  [2002] | NA |
| **Uzbekistan** | NA | 56·50  [2017] | 10·6 (95%UI 7·6–15·0)  [2021] | NA | NA | NA | NA |
| **UK- Wales** | NA | NA | 25·0 (95%UI 18·3–35·0)  [2021] | NA | NA | NA | NA |

In purple= minimally adequate treatment from modelling studies; in orange= epidemiological surveys; in blue= administrative records

Legend: ADHD= attention deficit hyperactivity disorder, MDD= major depressive disorder, NA= Not available; PTSD= Posttraumatic stress disorder, SE= standard error, 95%UIs=Uncertainty intervals, SUD= substance use disorder,

**Table S3. Number of included studies per country and corresponding World Bank 2025 income classification and geographical region**

| Country | n of studies | Income Classification | Geographical region |
| --- | --- | --- | --- |
| Albania | 1 | UMIC | Southeastern Europe |
| Andorra | 1 | HIC | Western Europe |
| Armenia | 1 | UMIC | Eastern Europe |
| Austria | 1 | HIC | Western Europe |
| Azerbaijan | 2 | UMIC | Eastern Europe |
| Belarus | 2 | UMIC | Eastern Europe |
| Belgium | 9 | HIC | Western Europe |
| Belgium, France, Germany, Italy, The Netherlands and Spain | 1 | HIC | Western Europe |
| Bosnia and Herzegovina | 2 | UMIC | Southeastern Europe |
| Bulgaria | 9 | HIC | Southeastern Europe |
| Croatia | 2 | HIC | Central Europe |
| Cyprus | 1 | HIC | Southeastern Europe |
| Czech Republic | 3 | HIC | Western Europe |
| Czechia | 3 | HIC | Central Europe |
| Denmark | 3 | HIC | Western Europe |
| England | 2 | HIC | Western Europe |
| Estonia | 1 | HIC | Western Europe |
| European (Italy, Germany, Hungary, Latvia, Poland and Spain) | 1 | MIXED | Western Europe |
| Finland | 3 | HIC | Western Europe |
| France | 11 | HIC | Western Europe |
| Georgia | 2 | UMIC | Eastern Europe |
| Germany | 10 | HIC | Western Europe |
| Greece | 1 | HIC | Southeastern Europe |
| Hungary | 1 | HIC | Central Europe |
| Iceland | 1 | HIC | Western Europe |
| Ireland | 1 | HIC | Western Europe |
| Israel | 6 | HIC | Southeastern Europe |
| Italy | 12 | HIC | Western Europe |
| Kazakhstan | 1 | UMIC | Eastern Europe |
| Kyrgyzstan | 1 | LMIC | Eastern Europe |
| Latvia | 1 | HIC | Western Europe |
| Lithuania | 1 | HIC | Western Europe |
| Luxembourg | 1 | HIC | Western Europe |
| Malta | 1 | HIC | Western Europe |
| Monaco | 2 | HIC | Western Europe |
| Montenegro | 1 | UMIC | Southeastern Europe |
| Netherlands | 12 | HIC | Western Europe |
| North Macedonia | 1 | UMIC | Western Europe |
| UK-Northern Ireland | 7 | HIC | Western Europe |
| Norway | 3 | HIC | Western Europe |
| Poland | 6 | HIC | Central Europe |
| Portugal | 11 | HIC | Western Europe |
| Republic of Moldova | 1 | UMIC | Eastern Europe |
| Romania | 8 | HIC | Southeastern Europe |
| Russian Federation | 2 | HIC | Eastern Europe |
| San Marino | 1 | HIC | Western Europe |
| UK-Scotland | 1 | HIC | Western Europe |
| Serbia | 1 | UMIC | Southeastern Europe |
| Slovakia | 2 | HIC | Central Europe |
| Slovenia | 2 | HIC | Central Europe |
| Spain | 10 | HIC | Western Europe |
| Spain- Murcia | 8 | HIC | Western Europe |
| Sweden | 4 | HIC | Western Europe |
| Switzerland | 4 | HIC | Western Europe |
| Tajikistan | 1 | LMIC | Eastern Europe |
| Turkey | 2 | UMIC | Southeastern Europe |
| Turkmenistan | 1 | UMIC | Eastern Europe |
| Ukraine | 3 | UMIC | Eastern Europe |
| United Kingdom | 1 | HIC | Western Europe |
| Uzbekistan | 2 | LMIC | Eastern Europe |
| UK-Wales | 1 | HIC | Western Europe |

Legend: hic=High-income country, UMIC= upper-middle income country, LMIC=low-middle income country

# **Additional information on the main sources**

The WHO Mental Health Atlas Project is an ongoing global effort, first launched in 2001 and updated periodically, most recently in 2020. Its primary aim is to collect, compile, and disseminate comprehensive data on mental health resources worldwide, including information on mental health policies, plans, financing, service delivery, human resources, availability of medicines, and information systems. The Atlas serves as the principal tool for monitoring progress by WHO Member States towards the objectives and targets of the WHO Comprehensive Mental Health Action Plan, with a particular focus on service coverage and system preparedness for mental health care.^1^

The WHO World Mental Health (WMH) Survey Initiative is a series of nationally representative community surveys conducted in 28 countries. These surveys use a standardized diagnostic interview (the WHO Composite International Diagnostic Interview, CIDI) to assess the prevalence, burden, and unmet need for treatment of common mental disorders. The WMH surveys provide internationally comparable data on the epidemiology of mental disorders, their impact on role functioning, and treatment gaps, thereby informing mental health policy and planning at both national and global levels. ^2,3^

1 WHO Team. Mental Health Atlas 2020. *WHO Publication* 2021; : 1–136.

2 Kessler RC, Aguilar-Gaxiola S, Alonso J, Chatterji S, Lee S, Üstün TB. The WHO World Mental Health (WMH) Surveys. *Psychiatrie (Stuttg)* 2009; **6**: 5.

3 Kessler RC, Aguilar-Gaxiola S, Alonso J, *et al.* The global burden of mental disorders: An update from the WHO World Mental Health (WMH) surveys. *Epidemiol Psichiatr Soc* 2009; **18**: 23–33.

**Table S4. Risk of bias of included studies according to RoB-PrevMH**

| study | domain1  _representativeness of the sample frame | support1 | doman2  _Representativeness of the responders | support2 | domain3_ Measurement of the condition | support3 |
| --- | --- | --- | --- | --- | --- | --- |
| Aagaard 2004 | low | Denmark and 2 regions. The study population was based on data from the Danish psychiatric central register | low | The study population was based on data from the Danish psychiatric central register. Compared to the average in Denmark, SJ county has a smaller fraction aged 20/30 years. In the Tønder region, relatively more are employed with farming and unskilled work. In the Aabenraa region, relatively more are employed with transportation or as public servants. The distribution of gender and age groups was equal between the Tønder and Aabenraa region | low | Epidemiological analysis: prevalence study using the national Danish Psychiatric Central Register. Socio-demographic data, staffing and prescribed services in the county and the municipalities psychiatry/social psychiatry were obtained from yearbooks and from a questionnaire investigation to the local administrations. |
| Alonso 2018 | low | nationally representative | low | nationally representative | low | WHO World Mental Health Survey Initiative, Trained lay interviewers conducted face-to-face interviews with respondents with validated tools |
| ATLAS | low | nationally representative | low | nationally representative | low | service coverage |
| Ballester 2020 | low | Web-based surveys were administered between October 2014 and October 2015 in a convenience sample of five public universities from different Autonomous Regions of Spain: Balearic Islands (UIB), Basque Country (UPV-EHU), Andalusia (UCA), Valencian (UMH), and Catalonia (UPF). These universities represented around 8% of the total number of students in public universities of Spain in the year 2014-15, and their distribution in terms of gender, nationality and academic field was similar to that of the overall population of students in public universities of Spain. The sample was recruited in two stages. In the first stage, all eligible students (i.e., census sampling) were invited to participate. In a second stage, a random subsample of non-respondents to the first stage was contacted offering an economic incentive to complete the survey | high | We found somewhat unbalanced distributions of the sample with respect to available census information, with higher proportions in the UNIVERSAL sample of females (72.5% vs. 55.2%), foreign students (5.3% vs. 3.2%), and health sciences students (25.6% vs. 15.8%). | low | validated scales |
| Barbato 2016 | high | Lombardy region is not representative of Italy | low | responders were representative | low | Survey-based research on access to psychosocial treatments (service utilization were retrieved from the psychiatric database of Lombardy, Italy, covering a population of 9,743,000, for all adults who had at least one contact in 2009 with psychiatric services). diagnoses are made with validated scales and codes (ICD). |
| Boerema 2016 | low | three Municipal Health Services (GGD) across different regions in the Netherlands (Amsterdam, Zoetermeer/Leidschendam, Dordrecht/ Gorinchem) | high | 291/1191 responded | low | The survey includes questions about physical health, psychosocial health, life-style, environment and the K10, a screening questionnaire for psychological distress [30]. Subjects who completed the Health Monitor in 2012 and who scored high on the K10 were invited to participate. A clinical diagnostic interview (CIDI 2.1) was conducted to determine whether subjects met criteria for a current major depressive disorder |
| Brazinova 2019 | low | Analysis of mental health care gaps and unmet needs from National Health Information Center (NHIC), 2. the Statistical Office and 3. the Social Insurance Agency. | low | Analysis of mental health care gaps and unmet needs from National Health Information Center (NHIC), 2. the Statistical Office and 3. the Social Insurance Agency. | low | validated scales (ICD-10) |
| Bruffaerts 2022 | low | nationally representative | low | nationally representative | low | WHO World Mental Health Survey Initiative, Trained lay interviewers conducted face-to-face interviews with respondents with validated tools |
| Cook 2020 | low | Data from the Russian census 2010 shows that the age distribution of the two cities was similar to the National average but the proportion of people with higher education was higher in Novosibirsk compared to the Urban Russian population as a whole while in Arkhangelsk it was similar. | low | representative | low | validated scales (PHQ-9) |
| Corrao 2021 | low | data covered 37% of the entire population, sampling in representative regions. | low | authomated system to access databases | low | data from administrative records on admissions and diagnosis. |
| Dauriac-Le Masson 2020 | low | Greater Paris area among homeless people. People who had slept in a place not intended for huma habitation or taken by organization. Random sampling drom different housing services, day centers and hot meal distribution points. | low | 840/859 Invited | low | validated scales (ICD-10) |
| de Vries 2021 | low | nationally representative | low | nationally representative | low | WHO World Mental Health Survey Initiative, Trained lay interviewers conducted face-to-face interviews with respondents with validated tools |
| Degenhard 2017 | low | nationally representative | low | nationally representative | low | WHO World Mental Health Survey Initiative, Trained lay interviewers conducted face-to-face interviews with respondents with validated tools |
| Demyttenaere 2008 | low | random sample of non-institutionalised adults | unclear | unclear | low | validated scales (CIDI interviw of the WHO) |
| Fayyad 2017 | low | nationally representative | low | nationally representative | low | WHO World Mental Health Survey Initiative, Trained lay interviewers conducted face-to-face interviews with respondents with validated tools |
| Font 2018 | low | 47 different sites | low | 100% respondends | low | validated scales (MINI) and asked if used mental heatlh treatment |
| Grigaite˙2024 | low | The WMHSIP was representative of the Portuguese adult population. | low | nationally representative | low | WHO World Mental Health Survey Initiative, Trained lay interviewers conducted face-to-face interviews with respondents with validated tools |
| Hamalainen 2008 | low | The two-stage stratified clustersampling frame comprised 8028 adults age 30 years and over living in mainland Finland. Regionally stratified according to the five university hospital regions, each serving approximately one million inhabitants. | low | The total number of included interviews was thus 6005, which is 75% of the original sample. | low | validated scales (CIDI interviEw of the WHO) AND Questions about health service use for mental problems during the past year (yes/no) covered use of specialist-level mental health services (including municipal services of psychiatric outpatient clinics, mental health centers, psychiatric hospitals, and private psychiatrists) and primary health care services (including among others, municipal health centers and occupational health services). |
| Harris 2024 | low | nationally representative | low | nationally representative | low | WHO World Mental Health Survey Initiative, Trained lay interviewers conducted face-to-face interviews with respondents with validated tools |
| Improving Access to Psychological Therapies (IAPT) programme | low | large-scale initiative. The IAPT programme’s robust dataset is based on patient-reported outcomes, which are routinely collected and recorded at each clinical session. All services are expected to achieve – and for the most part have achieved – very high levels of data collection (in excess of 90% in most cases) | unclear | unclear | unclear | The IAPT programme’s robust dataset is based on patient-reported outcomes, which are routinely collected and recorded at each clinical session. All services are expected to achieve – and for the most part have achieved – very high levels of data collection (in excess of 90% in most cases). no further info. |
| Kadzin 2023 | low | nationally representative | low | nationally representative | low | WHO World Mental Health Survey Initiative, Trained lay interviewers conducted face-to-face interviews with respondents with validated tools |
| Kagstrom 2019 | low | a two-stage sampling was used in order to arrive at a sample of nationally representative community-dwelling adults. A random sample of 850 voting districts was selected in the first stage and a random starting address was chosen in the second one | low | in total, 5,531 households were initially contacted; however, nobody was reached at home in 1,112 households (20%), thus 4,419 visits took place. From them, 756 (17%) households decided not to participate in the survey, 280 (6%) target participants refused to be interviewed, and 38 (1%) were not found. In addition, 39 (1%) respondents aborted the interview, which resulted in 3,306 completed interviews (response rate 75%) | low | Centrally trained staff conducted face-to-face paper and pencil interviews (PAPI). The prevalence of mental disorders was assessed through the fifth version of Mini International Neuropsy- chiatric Interview (M.I.N.I.) |
| Kilic 2024 | low | In October-November 2016, 420 Syrian refugees over the age of 18 were interviewed face-to-face in two neighborhoods in Ankara where Syrian refugees are concentrated. At the time the data were collected, an estimated 88,000 Syrian refugees were living in Ankara, according to data from the Directorate of Migration Management (2016). Since there was no reliable sampling frame for refugees, we decided to visit the households in these neighborhoods and include all those who volunteered to participate in the study. The only exclusion criterion was the presence of any condition that impeded understanding or communication. | low | A sampling frame or a sample size could not be computed; we tried to reach as many refugees as possible during the two-month study period. 15 of the visited households who refused to participate were not included in the study. Contact was made with 431 people from 229 households who agreed to participate in the study. 420 participated. | low | validated scales |
| Lora 2012 | high | Lombardy region is not representative of Italy | low | all data available via informatic system | low | Data on mental health services in Lombardy are routinely collected by the regional psychiatric information system. It is a well developed and well performing mental health information system implemented at region-wide level. |
| Mohler-Kuo 2016 | low | In 2010, roughly 93% of Swiss households had at least one landline telephone, with either listed (78%) or unlisted (15%) telephone numbers. To minimize sampling bias, we also included households with mobile telephones only (5%). Only about 2% of the households in Switzerland have neither a landline nor mobile telephone and were therefore excluded from our survey. Two-stage sampling was used. First, 13 734 valid addresses were successfully contacted and 10 533 households agreed to a short interview about household structure (response rate 76.7%). In the second stage, every second person in the household who met the criteria of targeted age and gender, according to the sampling design for each region, was selected to participate in a personal telephone interview. | low | A total of 12 418 eligible persons were identified from household interviews, of whom 10 038 participated in the survey (response rate 80.8%). | low | validated scales |
| Mortier 2024 | unclear | Recruitment for the T1 survey consisted of healthcare representatives contacting all employed HCW in each participating healthcare centre using administrative email distribution lists (i.e., census sampling). 6 Autonomous Communities in Spain (i. e., Andalusia, the Basque Country, Castile and Leon, Catalonia, Madrid, and Valencia), and including all types of HCW | unclear | A total of n = 8996 HCW participated at T1, representing a weighted (adjusted by achieved sample size) response rate of 11.7 %. A total of n = 4809 T1 participants also participated at T2 (i.e., 53.5 %); n = 3919 of T2 participants participated at T3 (i.e., 81.5 %); and n = 3183 of T3 participants participated at T4 (i.e., 81.2 %). For all surveys, two reminder emails were sent within 2–4 weeks after the initial invitation | low | validated scales |
| Murphy 2018 | high | Rapid appraisal study on barriers to mental health care utilizationConflict-affected regions, Mtskheta-Mtianeti. Participants from the first two groups were identified by snowball sampling i.e. identifying initial key stakeholders to invite as participants, who then provides the name of a subsequent participants, and so on. Mental health care users were sampled from the Mtskheta-Mtianeti region, about 30 k north-west of Tbilisi and home to the Tserovani IDP settlement, the largest settlement of 2008 IDPs, | high | The final number of key informants was n = 28 | low | validated scales |
| Öberg 2024 | low | Retrospective investigation of health care utilization of individuals who died by suicide in Sweden in 2015”, where we examined medical records from the two years p receding suicide, including psychiatric care, primary care and somatic care, from all major healthcare providers in the public and private sectors, as well as from non-major health care providers (e.g. private GPs offices). | low | access to all data | high | recorded medical info on coverage, but no info on psychiatric diagnoses if not available |
| Pitonák 2024 | low | Three different data collection methodologies were employed: (1) household probability sampling and computer-assisted personal interviewing, (2) panel sampling and computer-assisted telephone interviewing and (3) panel sampling and computer-assisted online interviewing. For personal interviewing, a two-stage sampling method was employed. The procedure involved randomly selecting a sample of voting districts and a random starting address in each of these. he samples were representative of the Czech adult population in terms of age, sex, education and region of residence. | high | The dataset consists of 3063 (response rate [RR] = 58.62%), 3248 (RR = 29.75%) and 1000 (RR = 8.97%) respondents who completed the personal, online and telephone versions of the survey, respectively. T | low | validated scales |
| Potocar 2024 | low | random sample of participants was selected from a random group of voting districts in order to arrive at a representative sample of adults (aged 18 or more years, with no upper limit for age) for the Czech general population in terms of age, gender, education and region | low | The response rate was 75% and a total 3 306 adults participated in the study. | low | validated scales (Mini) and asked if used mental heatlh treatment |
| Probst 2015 | low | GPs and their patients were representatively sampled in nine regions from six European countries (Italy, Germany, Hungary, Latvia, Poland and Spain) using complete registers or local GP associations. In some countries, GPs were randomly sampled from registers while in others sampling was stratified according to pre-selected criteria (e.g. urbanity, size of GP practice). | high | 56.4 % of contacted GPs refused study participation. | low | validated scales (CIDI) |
| Reich 2023 | low | representative of the population | unclear | unclear | low | An updated version of the fully standardized computerassisted Munich-Composite International Diagnostic Interview (DIA-X/M-CIDI [21, 22]) was conducted face-to-face by trained clinical interviewers accompanied by tablet-based self-administered lists and questionnaires (DIA-X-5/D-CIDI [23]) |
| Rens 2022 | low | A sample of 5000 inhabitants aged 15 to 80 years was invited to participate in a mental health survey. The sample was randomly drawn from the national register and was stratified by gender, municipality, age and nationality (Belgian versus non-Belgian). Urban and rural zones | high | 24% response rate | high | self reported |
| Roberts 2017 | low | Time–location sampling was chosen as a probabilistic method to recruit hard-to-reach and migrant populations (Fisher Raymond et al. 2007; Tyldum & Johnston, 2014). The sampling procedure involved contacting IDPs in the places of gathering (e.g., hostels for IDPs, state services, volunteer organisations and NGOs, places of the distribution of humanitarian aid). The sampling framework consists of time–location units which represent the potential universe of places, days and times, where and when target group can be accessed. | low | A total of 2203 questionnaires were completed and the overall response rate was 89%. The 58% of interviews took place in regional cities, 40% in other cities and towns, and 2% in villages. | low | validated scales |
| Santomauro 2024 | low | nationally representative | low | nationally representative | low | WHO World Mental Health Survey Initiative, Trained lay interviewers conducted face-to-face interviews with respondents with validated tools |
| Sebela 2020 | low | Briefly, two-staged sampling was used, where a random sample of participants was selected from a random group of voting districts in order to arrive at a representative sample of adults (aged 18 or more years, with no upper limit for age) for the Czech general population in terms of age, gender, education and region | low | 75% response rate, and the final sample consisted of 3,306 persons aged | low | validated scales (MINI) and asked if used mental heatlh treatment |
| Silva 2020 | low | nationally representative | low | nationally representative | low | WHO World Mental Health Survey Initiative, Trained lay interviewers conducted face-to-face interviews with respondents with validated tools |
| Stein 2020 | low | nationally representative | low | nationally representative | low | WHO World Mental Health Survey Initiative, Trained lay interviewers conducted face-to-face interviews with respondents with validated tools |
| Stein 2021 | low | nationally representative | low | nationally representative | low | WHO World Mental Health Survey Initiative, Trained lay interviewers conducted face-to-face interviews with respondents with validated tools |
| Stein 2023 | low | nationally representative | low | nationally representative | low | WHO World Mental Health Survey Initiative, Trained lay interviewers conducted face-to-face interviews with respondents with validated tools |
| Stolzenburg 2019 | low | We thus invited persons with symptoms of depression via newspaper advertisements, social media posts and handing out flyers to participate in our study. We focused our adverts on symptoms of depression because it is one of the most common mental disorders in the general population (Alonso et al. 2004) and many of its symptoms can be easily described without any psychiatric terminology. To ensure that undiagnosed persons could relate to the adverts, we described symptoms of depression in plain language, without mentioning the diagnosis or referring to psychiatry or mental illness | high | In total, 266 participants were invited, of which 31 (12%) did not attend after two or three follow-up calls and attempts to reschedule the interview, resulting in 233 persons completing the interview. | low | validated scales |
| ten Have 2004 | low | First, a sample was drawn of 90 Dutch municipalities, stratified on the basis of urbanicity and adequately distributed over the 12 Dutch provinces. The second step was to draw a sample of private households from post office registers there. The number of households selected in each municipality was determined by the size of its population. The third step was to choose which individuals to interview. The selected households were sent a letter of introduction signed by the national minister of public health asking them to take part. Shortly thereafter, they were contacted by telephone by the interviewers. | unclear | The response rate was 69.7% | low | validated scale (DSMamd asked about service use |
| Tuithof 2016 | low | First, a sample was drawn of 90 Dutch municipalities, stratified on the basis of urbanicity and adequately distributed over the 12 Dutch provinces. The second step was to draw a sample of private households from post office registers there. The number of households selected in each municipality was determined by the size of its population. The third step was to choose which individuals to interview. The selected households were sent a letter of introduction signed by the national minister of public health asking them to take part. Shortly thereafter, they were contacted by telephone by the interviewers. | unclear | The response rate was 69.7% | low | validated scale (DSMamd asked about service use |
| Wallerblad 2012 | low | In 1998-1999, 19742 randomly selected Swedish citizens aged 20–64 years, residing in the Stockholm County, were invited to participate and 10441 persons | low | (response rate 53%) responded to the self-administrated questionnaire (baseline) that included questions on demographic and socioeconomic characteristics, somatic and psychiatric health, and use of drugs. Three years after they had answered the first questionnaire (baseline) those who answered were reassessed with another similar questionnaire including questions on health care seeking; 8700 persons participated (retention rate 83%). | low | validates scales and asked about service use |
| Werlen 2020 | low | In order to obtain answers from participants of both Swiss and Non-Swiss nationality from all states (cantons), we developed a sampling plan stratified by canton, sex, and Swiss nationality that oversampled Non-Swiss participants, for which we adjusted in our statistical analyses. Based on this plan, the Swiss Federal Statistical Office provided us a random sample of 9805 young adults legally residing in Switzerland born between 1996 and 2000 (17–21 years old on December 31, 2017) who were randomly selected from the population register within each stratum specified by the sampling plan. | low | 3840 participants (41.4%) completed the online survey. | low | validates scales and asked about service use |

**Figure S1. Summary of RoB-PrevMH**

**Table S5. Trends of mental health coverage for any mental disorder over the years for countries with more than one estimate.**

| **Country** | **Percentage (95%UIs)** | |
| --- | --- | --- |
| **Czech Republic** | 17·78 (14·85 - 20·71) | 22·66 (20·83 - 24·49) |
| year | 2017 | 2022 |
| **Greece** | 25·6 (18·8–36·5) | 25·6 (18·8–35·8) |
| year | 2000 | 2021 |
| **Hungary** | 12·5 (9·2–17·7) | 13·4 (9·8–18·7) |
| year | 2000 | 2021 |
| **Iceland** | 25·9 (18·2–37·1) | 26·6 (19·3–37·1) |
| year | 2000 | 2021 |
| **Ireland** | 26·7 (19·0–38·7) | 30·1 (21·3–42·9) |
| year | 2000 | 2021 |

In purple: minimally adequate treatment

**Table S6. Trends of service coverage for psychosis over the years for countries with more than one estimate.**

| **Country** | **Percentage** | |
| --- | --- | --- |
| **Belarus** | 55·9 | 64·9 |
| **year** | 2017 | 2020 |
| **Finland** | 73·63 | 97·43 |
| **year** | 2017 | 2020 |
| **France** | 63·16 | 81·59 |
| **year** | 2017 | 2020 |
| **Italy** | 81·51 | 58·85 |
| **year** | 2017 | 2020 |
| **Monaco** | 96·84 | 83·68 |
| **year** | 2017 | 2020 |
| **Slovenia** | 71·81 | 64·98 |
| **year** | 2017 | 2020 |
| **Sweden** | 49·38 | 59·74 |
| **year** | 2017 | 2020 |

**Table S7. Trends of mental health coverage for major depressive disorder over the years for countries with more than one estimate.**

| **Country** | **Percentage (95%UIs)** | |
| --- | --- | --- |
| **Albania** | 12·4 (9·5–17·0) | 13·6 (10·4–18·2) |
| **year** | 2000 | 2021 |
| **Andorra** | 27·2 (19·1–39·3) | 27·7 (19·7–40·0) |
| **year** | 2000 | 2021 |
| **Armenia** | 10·8 (7·9–15·0) | 12·0 (8·7–16·6) |
| **year** | 2000 | 2021 |
| **Austria** | 25·8 (18·6–36·4) | 26·7 (19·5–37·5) |
| **year** | 2000 | 2021 |
| **Azerbaijan** | 9·6 (6·8–13·6) | 11·0 (7·9–15·9) |
| **year** | 2000 | 2021 |
| **Belarus** | 11·8 (8·7–16·7) | 13·5 (9·8–19·1) |
| **year** | 2000 | 2021 |
| **Belgium** | 32·4 (24·0–44·1) | 34·4 (25·2–47·6) |
| **year** | 2000 | 2021 |
| **Bosnia and Herzegovina** | 11·7 (8·6–16·6) | 12·8 (9·4–17·9) |
| **year** | 2000 | 2021 |
| **Bulgaria** | 8·7 (6·4–11·6) | 9·5 (6·9–13·0) |
| **year** | 2000 | 2021 |
| **Croatia** | 12·8 (9·4–18·1) | 13·8 (10·1–19·1) |
| **year** | 2000 | 2021 |
| **Cyprus** | 22·0 (15·6–31·9) | 26·8 (19·0–38·7) |
| **year** | 2000 | 2021 |
| **Czechia** | 13·1 (9·6–18·8) | 14·1 (10·2–19·6) |
| **year** | 2000 | 2021 |
| **Denmark** | 24·8 (17·8–35·2) | 26·0 (19·1–36·5) |
| **year** | 2000 | 2021 |
| **England** | 24·5 (20·8–29·4) | 25·8 (21·7–31·2) |
| **year** | 2000 | 2021 |
| **Estonia** | 12·0 (8·7–16·8) | 13·7 (10·0–19·0) |
| **year** | 2000 | 2021 |
| **Finland** | 17·9 (14·8–22·2) | 20·6 (15·2–29·0) |
| **year** | 2000 | 2021 |
| **France** | 26·2 (19·0–37·0) | 29·0 (21·3–40·7) |
| **year** | 2000 | 2021 |
| **Georgia** | 11·0 (8·0–15·3) | 11·4 (8·3–16·0) |
| **year** | 2000 | 2021 |
| **Germany** | 32·9 (24·7–43·7) | 35·0 (26·0–48·0) |
| **year** | 2000 | 2021 |
| **Greece**  **year** | 25·6 (18·8–36·5)  2000 | 25·6 (18·8–35·8)  2021 |
| **Hungary**  **year** | 12·5 (9·2–17·7)  2000 | 13·4 (9·8–18·7)  2021 |
| **Iceland**  **year** | 25·9 (18·2–37·1)  2000 | 26·6 (19·3–37·1)  2021 |
| **Ireland**  **year** | 26·7 (19·0–38·7)  2000 | 26·7 (19·0–38·7)  2021 |
| **Israel** | 18·0 (12·9–25·3) | 20·0 (14·6–28·3) |
| **year** | 2000 | 2021 |
| **Italy** | 19·9 (17·0–23·9) | 19·5 (16·6–23·4) |
| **year** | 2000 | 2021 |
| **Kazakhstan** | 9·7 (7·0–13·5) | 11·4 (8·2–15·9) |
| **year** | 2000 | 2021 |
| **Kyrgyzstan** | 9·4 (6·7–13·1) | 10·6 (7·5–15·0) |
| **year** | 2000 | 2021 |
| **Latvia** | 11·8 (8·6–16·6) | 12·9 (9·4–17·8) |
| **year** | 2000 | 2021 |
| **Lithuania** | 12·2 (8·9–17·2) | 12·9 (9·5–18·2) |
| **year** | 2000 | 2021 |
| **Luxembourg** | 25·6 (18·3–37·0) | 26·9 (19·3–38·2) |
| **year** | 2000 | 2021 |
| **Malta** | 23·6 (17·2–34·0) | 26·3 (19·6–36·4) |
| **year** | 2000 | 2021 |
| **Monaco** | 26·0 (19·3–36·8) | 26·2 (19·4–36·7) |
| **year** | 2000 | 2021 |
| **Montenegro** | 12·4 (9·0–17·1) | 13·5 (9·8–18·8) |
| **year** | 2000 | 2021 |
| **Netherlands** | 31·2 (24·0–39·2) | 30·3 (23·8–39·7) |
| **year** | 2000 | 2021 |
| **North Macedonia** | 11·6 (8·5–16·5) | 12·8 (9·3–17·8) |
| **year** | 2000 | 2021 |
| **Northern Ireland** | 24·5 (17·4–34·9) | 26·1 (18·7–37·4) |
| **year** | 2000 | 2021 |
| **Norway** | 25·5 (21·3–31·0) | 26·7 (22·5–32·3) |
| **year** | 2000 | 2021 |
| **Poland** | 11·5 (9·6–14·1) | 12·6 (10·6–15·2) |
| **year** | 2000 | 2021 |
| **Portugal** | 19·7 (14·4–27·6) | 21·8 (16·1–30·5) |
| **year** | 2000 | 2021 |
| **Republic of Moldova** | 11·1 (8·1–15·5) | 12·3 (8·9–17·2) |
| **year** | 2000 | 2021 |
| **Romania** | 15·4 (11·4–21·5) | 17·0 (12·4–24·0) |
| **year** | 2000 | 2021 |
| **Russia** | 10·9 (9·1–13·2) | 12·8 (10·8–15·6) |
| **year** | 2000 | 2021 |
| **San Marino** | 27·5 (20·1–39·7) | 27·3 (20·0–38·7) |
| **year** | 2000 | 2021 |
| **Scotland** | 23·7 (17·3–34·1) | 25·7 (18·6–36·8) |
| **year** | 2000 | 2021 |
| **Serbia** | 11·7 (8·6–16·4) | 12·8 (9·4–17·7) |
| **year** | 2000 | 2021 |
| **Slovakia** | 12·1 (8·9–17·1) | 13·4 (9·9–19·0) |
| **year** | 2000 | 2021 |
| **Slovenia** | 13·2 (9·8–18·6) | 15·0 (10·9–20·7) |
| **year** | 2000 | 2021 |
| **Spain** | 23·3 (17·4–31·7) | 23·8 (17·9–33·3) |
| **year** | 2000 | 2021 |
| **Sweden** | 27·2 (19·9–38·2) | 27·7 (20·5–39·3) |
| **year** | 2000 | 2021 |
| **Switzerland** | 26·6 (18·9–37·9) | 27·9 (20·6–38·7) |
| **year** | 2000 | 2021 |
| **Tajikistan** | 8·8 (6·2–12·6) | 9·8 (7·0–14·1) |
| **year** | 2000 | 2021 |
| **Turkey**  **year** | 4·6 (3·1–6·9)  2000 | 5·5 (3·8–7·9)  2021 |
| **Turkmenistan** | 9·0 (6·4–12·8) | 9·9 (7·0–14·1) |
| **year** | 2000 | 2021 |
| **Ukraine** | 11·8 (9·7–14·3) | 12·6 (10·6–15·3) |
| **year** | 2000 | 2021 |
| **Uzbekistan** | 9·4 (6·6–13·3) | 10·6 (7·6–15·0) |
| **year** | 2000 | 2021 |
| **Wales** | 24·2 (17·7–34·4) | 25·0 (18·3–35·0) |
| **year** | 2000 | 2021 |

In purple: minimally adequate treatment
